# Understanding the Role of Growth Factors in Modulating Stem Cell Tenogenesis

**DOI:** 10.1371/journal.pone.0083734

**Published:** 2013-12-30

**Authors:** Ana I. Gonçalves, Márcia T. Rodrigues, Sang-Jin Lee, Anthony Atala, James J. Yoo, Rui L. Reis, Manuela E. Gomes

**Affiliations:** 1 3B’s Research Group, Department of Polymer Engineering, Univ. of Minho, Headquarters of the European Institute of Excellence on Tissue Engineering and Regenerative Medicine, Guimarães, Portugal; 2 ICVS/3B’s - PT Government Associate Laboratory, Braga/Guimarães, Portugal; 3 Wake Forest Institute for Regenerative Medicine, Wake Forest School of Medicine, Winston-Salem, North Carolina, United States of America; University of Minho, Portugal

## Abstract

Current treatments for tendon injuries often fail to fully restore joint biomechanics leading to the recurrence of symptoms, and thus resulting in a significant health problem with a relevant social impact worldwide. Cell-based approaches involving the use of stem cells might enable tailoring a successful tendon regeneration outcome. As growth factors (GFs) powerfully regulate the cell biological response, their exogenous addition can further stimulate stem cells into the tenogenic lineage, which might eventually depend on stem cells source. In the present study we investigate the tenogenic differentiation potential of human- amniotic fluid stem cells (hAFSCs) and adipose-derived stem cells (hASCs) with several GFs associated to tendon development and healing; namely, EGF, bFGF, PDGF-BB and TGF-β1. Stem cells response to biochemical stimuli was studied by screening of tendon-related genes (collagen type I, III, decorin, tenascin C and scleraxis) and proteins found in tendon extracellular matrix (ECM) (Collagen I, III, and Tenascin C). Despite the fact that GFs did not seem to influence the synthesis of tendon ECM proteins, EGF and bFGF influenced the expression of tendon-related genes in hAFSCs, while EGF and PDGF-BB stimulated the genetic expression in hASCs. Overall results on cellular alignment morphology, immunolocalization and PCR analysis indicated that both stem cell source can be biochemically induced towards tenogenic commitment, validating the potential of hASCs and hAFSCs for tendon regeneration strategies.

## Introduction

Tendons are highly prone to injury and their intrinsic hypocellularity and hypovascularity makes their natural healing extremely slow and inefficient when severely damaged. Surgical repair with grafts is common but unsuccessful in a long term basis as the biochemical and mechanical properties of healed tendon tissue never match those of intact tendon, ultimately resulting in the progression of degenerative diseases, such as osteoarthritis [1].

The regenerative mechanism underneath the unique organization of collagen fibers and resident cell alignment in between the fibers is still unknown. Thus, the limited ability of tendon to self-repair and the limitation of treatment regimens have hastened the motivation to develop stem cell-based strategies that explore the natural endogenous system of tissue regeneration.

Amniotic fluid stem cells (AFSCs) have shown to be highly proliferative, exhibiting high self-renewal capability and potential to differentiate into several lineages [2]. In addition, human AFSCs are easy to obtain, representing an almost unlimited stem cell source with immunosuppressive properties [3].

Adipose tissue is also a promising source of stem cells as adipose-derived stem cells (ASCs) have been explored for therapeutic applications, and may represent a potential choice for tendon repair and regeneration [4]. Tissue availability, easy and minimally invasive access to adipose sources place these cells in a unique position relative to other MSCs in the tissue engineering and regenerative medicine (TERM) field. Moreover, human ASCs (hASCs) isolation is a simple and relatively easy enzyme-based methodology, and evidences suggest an immune-privileged behavior [5].

We and others have demonstrated that under appropriate inductive conditions human AFSCs [2], [6], [7] and hASCs can be directed into several skeletal tissue-related lineages, such as bone [2], [6]–[8] and cartilage [2], [6], [8].

It is widely accepted that several different environmental factors contribute to the overall control of stem cell activity [9]. Growth factors (GFs) are potential agents to target specific tissue reactions because of their regulatory roles in cellular functions, including adhesion, proliferation, migration, matrix synthesis, and cell differentiation [10]. For instance, fibroblast- (FGF), platelet derived- (PDGF) and transforming- (TGF-β) growth factors are markedly upregulated throughout tendon repair mechanisms [11].

Since growth factors such as epidermal-(EGF), FGF, PDGF and TGF-β have been described to play a role in tendon development and tendon healing, they are to be investigated in this study.

EGF is a potent mitogen that participates in MSCs and fibroblast proliferation [12], [13], and is also involved in the initial phase of tendon healing. Besides MSCs proliferation, EGF treatment also preserves early progenitors within a MSC population [12], and increases the paracrine activity of stem cells.

bFGF was recently described to maintain an undifferentiated state of ligament stem cells (LSCs) [14]. Also, LSCs proliferate faster with bFGF treatment [12], [14]. FGF signaling is required for the early stages of differentiation in a number of lineages and is also an essential mediator of self-renewal in human stem cells [14]. Additionally, bFGF stimulates the production of collagenous and non-collagenous ECM [15], thus evidencing a role in proliferation and tendon commitment.

*In vitro* studies suggest that bFGF and PDGF not only stimulate tendon fibroblast proliferation but promote changes in the expression of matrix genes showing promise for improving tendon healing [16]. PDGF also plays a role in the migration and proliferation of the tenocytes, fibroblasts, and MSCs responsible for tissue homeostasis. Furthermore, PDGF modulates the synthesis of ECM [17] and supports the formation of a vascular network, which sustains biofunctional and physiological integrity [18] of the tissue. Its biological action highlights the PDGF potential to treat and enhance the biologic response of injured tendons and ligaments [19].

TGF-β has also been attracting attention in the tenogenic regenerative field as TGF-β participates in all three phases of tendon healing process: inflammation, proliferation and remodeling [20]. Moreover, TGF-β has been described to be involved in tendon formation [21], to induce tendon markers in mesenchymal cells [22] and to stimulate upregulation of gene expression and production of ECM in LSCs [14], playing a role in tendon cell fate. The three isoforms of TGF-β (TGF-β1, TGF-β2, TGF-β3) were shown to participate on collagen production and cell viability [23]. In particular, TGF-β1 increased the production of collagenous and non-collagenous extracellular matrix protein in LSCs [16].

Expanding and culturing cells while maintaining a tenogenic phenotype would be useful in producing a more efficient tendon bioengineered substitute. Although EGF, FGF, PDGF and TGF-β were described to contribute to tendon and ligament development and healing [1], [21], the exact nature of tendon regeneration remains unknown. Furthermore, the exogenous addition of GFs to the cellular microenvironment could provide a trigger to assist the differentiation of multipotent cells into a tenogenic lineage, and establish a biochemical link between cells and native tissue, thus participating in the process of restoring tendon functionality.

In this study we propose to assess the tenogenic potential of human amniotic fluid-derived stem cells (hAFSCs) and human adipose-derived stem cells (hASCs) in the presence of specific biochemical culture conditions that might be used in cell-based strategies for tendon repair.

We hypothesize that the exposure to the proper biochemical cues, that is, GFs that participate in tendon formation and ECM synthesis, would potentially stimulate tenogenic differentiation of stem cells. Furthermore, the inclusion of these GFs in the culture medium would enhance the expression of tendon- related markers and the synthesis of tendon-like ECM. The successful tenogenic differentiation of stem cells also outcomes for cell-laden scaffolding strategies towards assisting and/or improving regeneration *in locus*. An accelerated proliferation and remodeling process could improve gliding and strength enhancement at the injury site and simultaneously reduce the risk of fibrosis and tendon failure during the repair/regenerative process.

## Materials and Methods

### Stem Cell Isolation and Expansion

Human amniotic fluid stem cells (hAFSCs) were obtained from human amniotic fluid specimens collected during amniocentesis procedures. The amniotic fluid was obtained under an IRB protocol approved by Wake Forest School of Medicine. Back-up human amniocentesis cultures, that would otherwise be discarded, were harvested by trypsinization. Within the pool of hAFSCs the c-Kit (CD117) positive population was immunoselected with magnetic microspheres, whose protocol has been described in detail elsewhere [2]. hAFSCs were assessed for several markers by flow cytometry and showed to be negative for CD45, CD33 and CD 133, and positive for CD73, CD90, CD105, CD29 and CD44 [2]. Then, hAFSCs were expanded and cryopreserved. The basic amniotic fluid cell (BAFC) medium was composed by α-MEM (Invitrogen) plus 15% embryonic screened FBS (ES-FBS, Fisher Scientific), 1% glutamine (Sigma), 1% antibiotic/antimycotic (A/A) solution (Gibco), 18% Chang B (Izasa/C101) and 2% Chang C (Izasa/C108) at 37°C with 5% CO_2_ atmosphere.

Human ASCs were obtained from lipoaspirate samples of the abdominal region, under protocols previously established with Hospital da Prelada (Porto, Portugal) and with informed consent of the patients. The content of the written informed consent and related procedures were reviewed and approved by the Hospital Ethics Committee.

Cells were isolated from tissue samples and cultured as described before [22], and have been previously characterized by RT-PCR for CD44, STRO-1, CD105 and CD90 markers [22]. Briefly, the tissue was rinsed in phosphate-buffered saline (PBS, Sigma-Aldrich) containing 10% of an antibiotic-antimycotic solution (Gibco). The fat solution was immersed in a 0.05% collagenase type II (Sigma/C6885) solution for 45 minutes at 37°C under mild agitation. The digested tissue was centrifuged at 304 g for 10 minutes at 4°C, after which the supernatant was eliminated. Lysis buffer was used to dissolve the pellet followed by a centrifugation at 304 g for 5 minutes. Cells were expanded in basic medium composed of α-MEM (Invitrogen) supplemented with 10% FBS (Alfagene), and 1% A/A solution (Alfagene).

The data obtained from amniocentesis back-up cultures and from lipoaspirate samples was analyzed anonymously.

After reaching a sufficient cell number (approximately 380,000 cells),hAFSCs and hASCs were cultured in media conditioned with different growth factors known to participate in tendon healing mechanisms [1], as described in **Table 1**. The growth factor’s concentration of 10 ng/mL was selected with basis on previously published reports on tendon and ligament regeneration strategies [14], [24]–[26]. Thus, in this work we considered 10 ng/mL studied as the minimum concentration of growth factor to likely influence cellular differentiation.

**Table 1:** Description of the culture medium composition to induce the tenogenic potential of hAFSCs and hASCs.

*Medium*	*Description*
***A)***	**Basic medium:** α-MEM, FBS (10%), A/A (1%)
***B)***	**AFSCs expansion medium (BAFC):** α-MEM +15% ES-FBS, 1% glutamine, 1% antibiotic, 18% Chang B and 2% Chang C
***C)***	**hASCs:** basic medium A+glutamine (2 mM) and ascorbic acid (0.2 mM)
***D)***	**Basic medium A+**glutamine (2 mM)+ascorbic acid (0.2 mM)+EGF (10 ng/ml)
***E)***	**Basic medium A+**glutamine (2 mM)+ascorbic acid (0.2 mM)+bFGF (10 ng/ml)
***F)***	**Basic medium A+**glutamine (2 mM)+ascorbic acid (0.2 mM)+PDGF-BB (10 ng/ml)
***G)***	**Basic medium A+**glutamine (2 mM)+ascorbic acid (0.2 mM)+TGF-β1 (10 ng/ml)

***α-MEM*** *: Minimum Essential Medium Eagle - Alpha Modification;* ***FBS*** *: fetal bovine serum;* ***A/A*** *: antibiotic/antimicotic solution;* ***FBS-ES*** *: fetal bovine serum embryonic screened;* ***EGF*** *: endothelial growth factor (Peprotech/100-15);* ***bFGF*** *: basic-fibroblast growth factor (Peprotech/100-18B);* ***PDGF-BB*** *: platelet-derived growth factor (eBioscience/14-8501);* ***TGF-β1*** *: transforming growth factor-β1 (eBioscience/14-8348).*

The inclusion of ascorbic acid in the culture medium has been associated with an increased MSCs proliferation and human collagen synthesis, thus a positive and promising factor aiming at a successful tenogenic medium.

Tenogenic differentiation was weekly evaluated up to 28 days based on cell morphology and on the presence of Tenascin C, Collagen I and Collagen III proteins, as well as on PCR analysis for tendon-related markers (scleraxis, tenascin C, decorin, collagen type I and collagen type III), as described in detail below.

### Morphological Analysis

Human AFSCs and hASCs were monitored daily and photographs were obtained from live cells collected weekly using a phase contrast microscope (Axiovert 40 CFL, Zeiss) (Figure S1 and S2). Multiple regions within each sample well were observed and representative sections captured by a digital camera (PowerShot G11, Canon).

### Immunolocalization of ECM Proteins: Collagen I, Collagen III and Tenascin C

Samples from each culture condition were rinsed in PBS, fixed in a 10% buffered formalin solution (43.05-k01009, INOPAT) overnight and kept in PBS at 4°C until usage.

Collagen I (Rabbit polyclonal Anti-Collagen I, ab292, Abcam), Collagen III (Monoclonal Anti-Collagen, Type III, C7805, Sigma-Aldrich) and Tenascin C (Mouse monoclonal Anti-Tenascin C antibody [BC-24], ab6393, Abcam) expression was assessed on cells cultured onto tissue culture treated 6-well plates (Falcon). After cell permeabilization with 0.025% Triton-X100 (Sigma/X100)/PBS solution, the blocking step was performed using RTU Normal Horse Serum (RTU Vectastain Kit, PK-7200, Vector). Then, cells were incubated overnight with the primary antibodies above mentioned, diluted in antibody diluent with background reducing components from Dako (Dako) at 4°C.

The dilution ratio was optimized to 1∶3000, 1∶500, 1∶3000 for Tenascin C, Collagen I and Collagen III antibodies, respectively.

Afterwards, samples were rinsed in PBS, following inactivation of endogenous peroxidase activity with hydrogen peroxide solution (0.3% w/v, Panreac). The samples were incubated for 1 hour at room temperature with the respective fluorescent secondary antibody (rabbit anti-mouse Alexa Fluor 488/A11059, or donkey anti-mouse Alexa Fluor 594/A21203, Invitrogen; dilution 1∶200), considering the host species of the primary antibodies.

After the incubation with secondary antibodies, samples were rinsed in PBS and stained with 4,6-Diamidino-2-phenyindole, dilactate (DAPI, 5 µg/µl, D9564, Sigma) for 10 minutes. Negative controls assessed for immunofluorescence detection were incubated in Dako diluent in the absence of the primary antibody.

Finally, samples were incubated with a Phalloidin–Tetramethylrhodamine B isothiocyanate (Phalloidin) solution, which was prepared accordingly to manufacturer’s instructions (P1951, Sigma; dilution 1∶200).

All samples were observed under a microscope (Imager Z1m, Zeiss) and images were acquired using a digital camera (AxioCam MRm5). The total growth surface area (9.6 cm^2^) of each well was screened under the microscope. A minimum of 2 wells per sample, condition and endpoint were analyzed. Also, a minimum of 2 samples per independent experiment (n = 3) were investigated for protein detection by immunofluorescence.

### RNA Isolation and Gene Expression Analysis

Total RNA was extracted using TRI Reagent® RNA Isolation Reagent (T9424, Sigma) following the manufacture’s instruction. RNA was quantified on a Nanodrop® ND-1000 spectrophotometer (Thermo Scientific) and first-strand complementary DNA was synthesized from 1 µg of RNA of each sample (qScript™ cDNA Synthesis Kit, Quanta Biosciences) in a 20 µL reaction using a Mastercycler® ep realplex gradient S machine (Eppendorf). Reverse transcription followed by the polymerase chain reaction (RT-PCR) was the technique selected to analyze mRNA expression derived from cells cultured in different media. RT-PCR was performed to assess the gene expression of typical markers for tenogenic differentiation, namely collagen type I, collagen type III, tenascin C, decorin and scleraxis. The transcript expression of target genes was analyzed and normalized to the expression of endogenous housekeeping gene GAPDH (glyceraldehyde-3-phosphate dehydrogenase) (n = 3). The primers were designed with Primer 3 software (**Table 2**) and synthesized by MWG Biotech.

**Table 2:** Primers used for quantitative RT-PCR analysis.

Target gene	Gene Abbreviation	Forward Primer	Reverse Primer	Accession number
**GAPDH**	GAPDH	tgtaccaccaactgcttagc	ggcatggactgtggtcatgag	MN 002046.4
**collagen, type I,** **alpha 1**	COL1A1	agccagcagatcgagaacat	acacaggtctcaccggtttc	NM_000088.3
**collagen, type III,** **alpha 1**	COL3A1	gggaacatcctccttcaaca	gcagggaacaacttgatggt	NM_000090.3
**tenascin C**	TNC	gttaacgccctgactgtggt	ccacaatggcagatccttct	NM_002160.3
**decorin**	DCN	gccattgtcaacagcagaga	cgagtggtccagtgttctga	NM_001920.3
**scleraxis homolog A**	SCXA	tgccttgcagcctcttactt	ctcccagtggagtgtggagt	BK_000280.1

A 2^−ΔΔCt^ method was used to evaluate the relative expression level for each target gene. ΔCt values were obtained by the difference between the Ct values of target genes and the GAPDH gene. These values were then normalized by subtracting the ΔCt value of the calibrator sample, their respective Ct value in basic medium condition, to obtain ΔΔCt values. Results are represented as relative gene expression in comparison to calibrator sample that is equal to 1.

### Statistical Analysis

All quantitative results are expressed as the mean ± standard deviation.

Two-Way ANOVA followed by Bonferroni’s Multiple Comparison test were assessed to determine whether differences between sample groups were significant. Differences were considered significant when the p value was <0.05.

## Results and Discussion

### Morphological Analysis and Cytoskeletal Arrangement of Tenogenic Induced Cells

**Figure 1:**
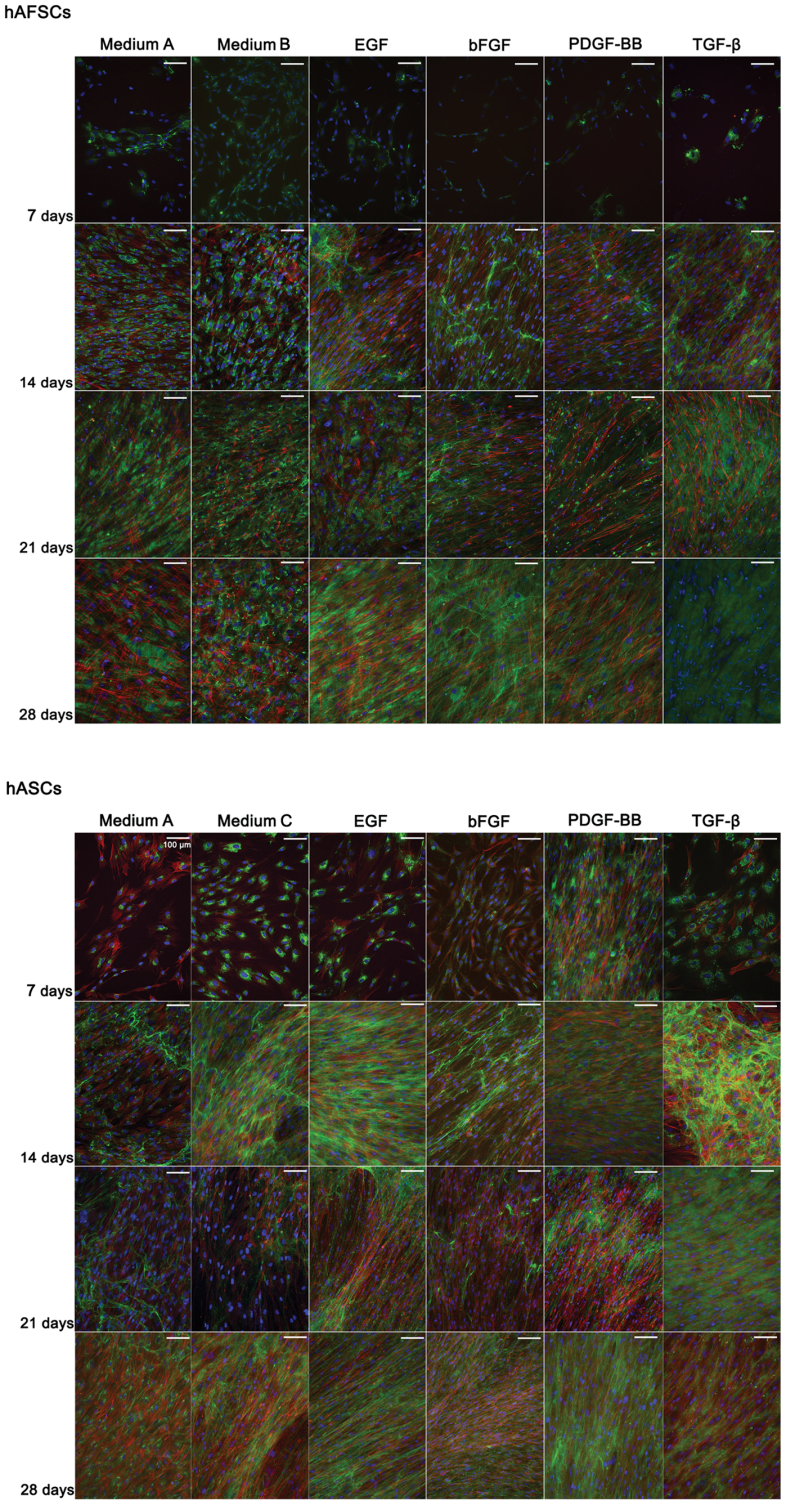
Collagen I immunolocation in hAFSCs and hASCs cultured up to 28 days in different supplemented media. DAPI (blue)and phalloidin-conjugate (red) stain cell nucleus and cytoskeleton, respectively. Collagen I is stained in green and represents the major tendon ECM protein. Scale bar represents 100 µm. Magnification: 200 x.

The typical morphology of tendon cells corresponds to a spindle–like shape longitudinally oriented to tendon axis [21]. Since the morphologic features are important for achieving a functional tissue, we investigated stem cell morphology when cultured in the different conditioned media. To reveal the cytoskeleton organization in response to the various GFs supplemented media, cells were stained with phalloidin (**Figure 1**).

### Differential Cytoskeleton Alignment in Distinct Stem Cell Sources

Overall, both hAFSCs and hASCs developed an extensive network of actin fibers by 14 days in culture. Nevertheless, cell alignment was more evident in hAFSCs than in hASCs cultures. By 21 days, both hAFSCs and hASCs evidenced an alignment pattern in culture medium with PDGF-BB and TGF-β1. This pattern was also observed in basic medium (A) and in medium with bFGF in hAFSCs, but slightly faded in hASCs cultured in basic medium (C). After 28 days in culture, hASCs alignment could be detected in all culturing media, although in medium with PDGF-BB the phalloidin stain was less intensive. For hAFSCs the evidence of aligned distribution was only observed in medium supplemented with EGF and PDGF-BB.

Interestingly, hAFSCs and hASCs evidenced an aligned distribution in basic medium by 14 days and 28 days in culture, respectively, indicating that these GFs are important but not essential in achieving an aligned morphology of these cells.

Phalloidin selectively binds cell actin filaments and the actin cytoskeleton has been suggested to have a relevant participation in the alignment and organization of the collagenous ECM in embryonic tendon [27]. Moreover, the longitudinal organization of actin fibers within cell rows (observed in Figure S1 and Figure S2) is a promising feature for sensing the tensile loads naturally exerted by the muscle to the bone, transmitted to the tendon tissue. Since actin fibers and cytoskeletal tension are often associated, the synthesis of cytoplasmic mechanical fibers (resultant from cytoskeletal rearrangement to meet the extracellular environmental conditions) will ultimately be translated into biochemical signals, which would trigger cell differentiation mechanisms.

### Immunolocalization of ECM Proteins: Collagen I, Collagen III and Tenascin C

The resident cells of mechanically functional tissues are often responsible for the production and maintenance of the ECM, including the collagen fibers. Collagen type I, Collagen type III and Tenascin C are present and play a role in the ECM of native tendon tissues. Thus, the immunolocalization of these proteins was assessed as a tool to characterize the tendon-like matrix synthesized by stem cells and stimulated into the tenogenic lineage.

### Stem Cells Develop a Collagen I Rich Extracellular Matrix in Different Supplement Media

During tendon development, collagen fibrillogenesis generates a tendon-specific ECM that determines the functional intrinsic mechanic properties of the tissue through cellular deposition of parallel arrays of collagen fibrils.

The immunofluorescence analysis revealed that both hAFSCs and hASCs developed a Collagen I-rich matrix in a timeline sequence (Figure 1). The synthesis of collagen fibrils occurred first as an intracellular step with assembly and secretion of procollagen [28]. By 7 days in culture, Collagen I fluorescence signal was concentrated around the nuclei, which likely corresponds to the synthesized pro-collagen chains. Despite variations in the fluorescence signal, it occurred in both hAFSCs and hASCs and in all conditioned media. Beyond 14 days, Collagen I was detected outside hASCs in basic medium (C) and in media supplemented with EGF, bFGF and PDGF-BB. Only in GFs supplemented media the extracellular collagen synthesized by hAFSCs was observed, and seemed to have an aligned fibrillar-like shape (Figure 1). This process can be associated to the extracellular step of collagen matrix production, where the pro-collagen is converted into collagen and subsequent incorporation into stable cross-linked collagen fibrils [28].

Extracellular collagen showed two distinct distribution patterns, corresponding to a either aligned or randomly oriented matrix network (Figure 1). The aligned pattern of Collagen I in hASCs was widely observed in supplemented culture media with the exception of medium with TGF-β1 after 14 days in culture. In basic media (A and C) the collagen matrix was more randomly dispersed. In hAFSCs the collagen distribution was more randomly oriented after 21 days in culture, despite the saturation of collagen outside the cells.

Overall, Collagen I was detected in both cell types and in all conditioned media throughout the experimental timeline and the collagenous matrix seemed to increase throughout the time in culture.

### Collagen III

Besides Collagen I, Collagen III has also an important role in fibril formation. Collagen III copolymerizes with Collagen I, and despite its low amount in the ECM, it provides tissue elasticity. Although the immunolocalization of Collagen III was assessed in this study, in general its fluorescence signal was very mild (data was not shown).

### Tenascin C

Tenascin C is an ECM protein highly regulated by the tissue microenvironment [29]. Although rarely present in most adult tissues, Tenascin C is upregulated in embryonic and developing tissues, or in tissues experiencing a fast rate of growth, and influences cell adhesion and migration.

**Figure 2:**
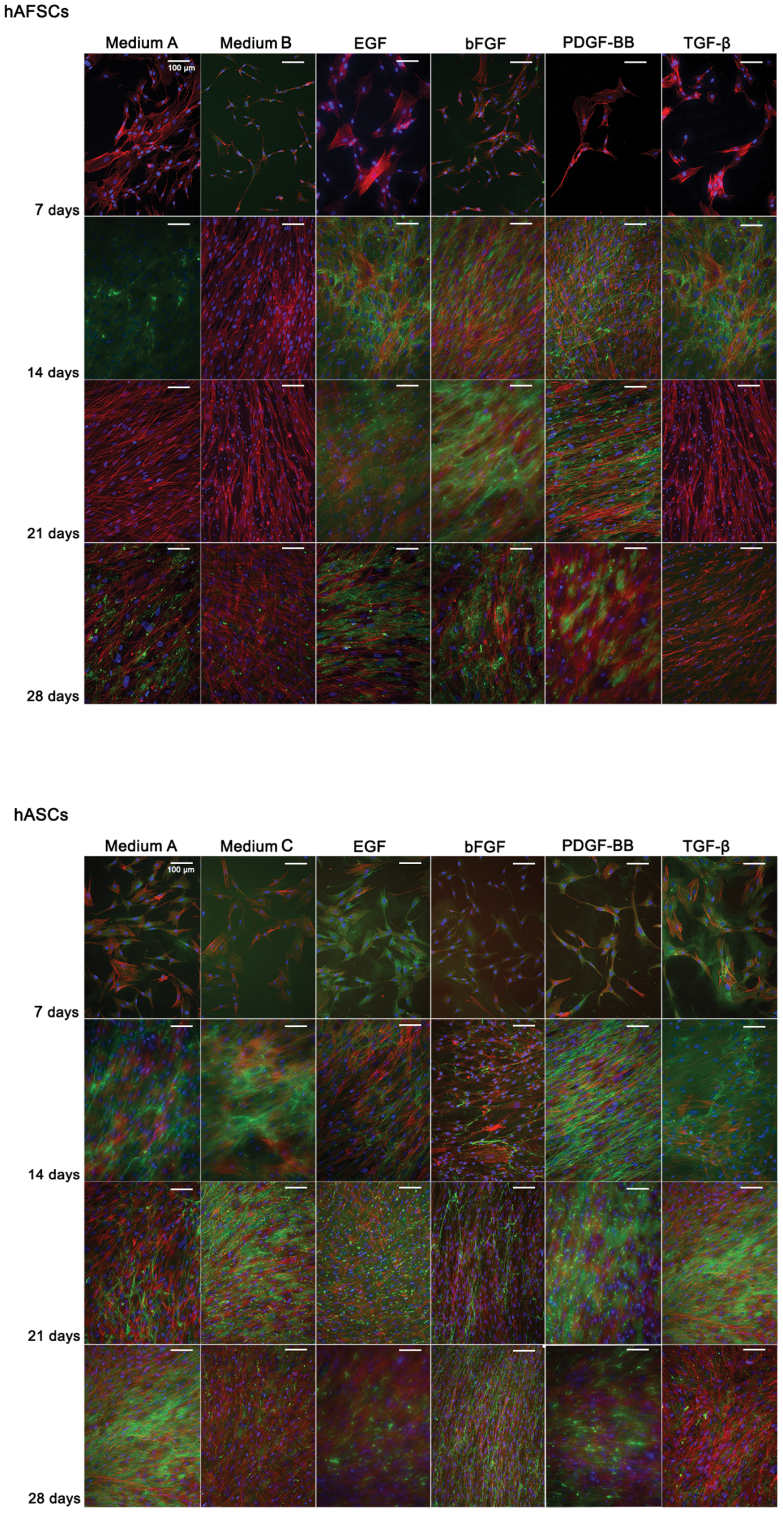
Tenascin C immunolocation in hAFSCs and hASCs cultured up to 28 days in different supplemented media. DAPI (blue)and phalloidin-conjugate (red) stain cell nucleus and cytoskeleton, respectively. Tenascin C is stained in green and represents a tendon ECM protein. Scale bar represents 100 µm. Magnification: 200 x.

The fluorescent signal of Tenascin C was neglectable in hAFSCs by 7 days in culture (Figure 2). After 2 weeks, the expression increased in all culture conditions except in the presence of bFGF. After 21 days, Tenascin C was not detected in basic media (A and B) or in TGF-β1 supplemented medium. Thus, medium supplemented with EGF, bFGF or PDGF-BB seemed to influence Tenascin C production in hAFSCs for longer periods of culture.

Similarly, Tenascin C was residually detected in hASCs after 7 days in the conditioned cultures. Nevertheless, the protein detection tended to increase with the time in culture up to 21 days, being the strongest signal detected in EGF, PDGF-BB and TGF-β1 culture media.

The expression of Tenascin C did not seem to be associated to the alignment of cells *per se*. Another supportive data for this statement relied on the fact that the Tenascin C protein network is continuously synthesized, as observed by an increased fluorescence signal in later time points, especially in hAFSCs. Despite the fact that cells were proliferating, especially in longer culturing times, the confluence of the cells on did not show to arrest proliferation or detaching from the culture plate’s surface, as it is commonly reported for other cell types.

### Real Time RT-PCR

The immunolocalization procedures for ECM detection were complemented with RT-PCR analysis of tendon-related markers in order to enhance information and eventually establish a timeline event for tenogenic differentiation on a molecular biology and protein basis.

### Decorin

Decorin is a proteoglycan that regulates tendon structure by stabilizing and aligning collagen fibrils [30].

**Figure 3:**
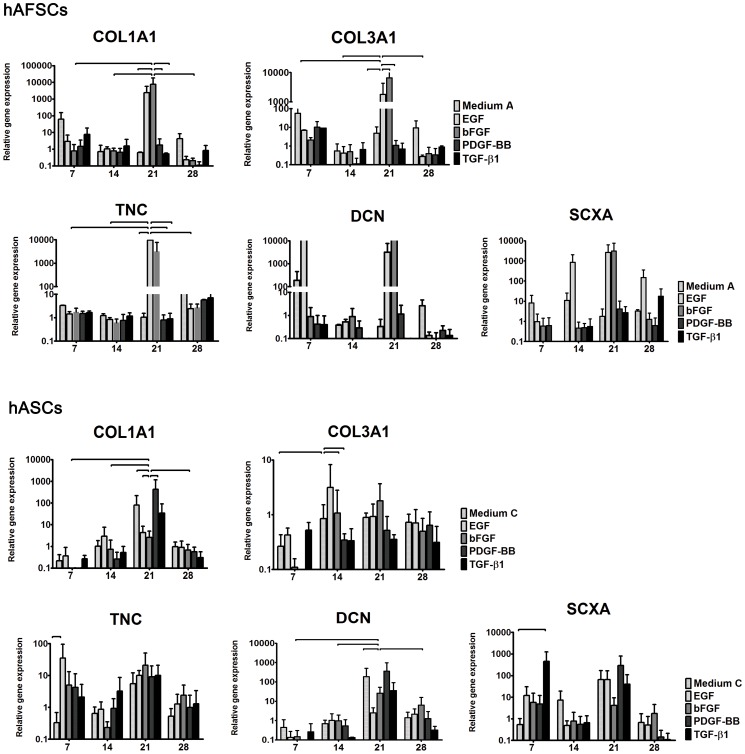
Real time RT-PCR analysis. Expression of tenascin C (TNC), decorin (DCN), collagen type I (COL1A1), collagen type III (COL3A1) and scleraxis (SCXA) genes in hAFSCs and hASCs cultured in different supplemented media. The x axis represents the culture time, namely 7, 14, 21 and 28 days. The relative gene expression is represented in the y axis. Horizontal lines represent differences statistically significant for p<0.05.

The decorin expression in hAFSCs was evident as early as 7 days in basic (A) and in EGF supplemented medium (**Figure 3**). By 14 days, decorin values were similar to the GAPDH’s, and increased again by 21 days in EGF and bFGF media. Only in basic medium (A) hAFSCs expressed decorin after 4 weeks in culture.

Conversely, decorin expression of hASCs only increased after 21 days in culture (Figure 3). The highest values were detected in PDGF-BB and basic (C) media, followed by TGF-β1, bFGF and EGF, respectively. Then, decorin expression decreased after 28 days for all studied media yet with increased values when compared to the 14 day time point.

The influence of EGF medium can be explained by decorin involvement in signal transduction through the EGF receptor. Moreover, supplemented PDGF-BB is likely to participate in the ECM synthesis by increasing decorin and collagen type I expression in hASCs. PDGF-BB has been described to modulate both fibroblast proliferation and ECM synthesis [18]. Although TGF-β1 is known to interact with decorin, the functional significance of this interaction is still unclear [31].

### Collagen Type I and Type III

The major fibrillar component of tendon is type I collagen [21], and the synthesis of collagen type I is the crucial step in determination of the tensile strength of tendons [32].

In hASCs, collagen type I has an increased expression around 21 days in culture (Figure 3). The highest values were found for cells cultured in PDGF-BB, basic (C) and TGF-β1 media, respectively. Furthermore, collagen type I expression in hAFSCs reached a peak by 21 days in EGF or bFGF media, although some expression was detectable as early as 7 days in basic (A) and in TGF-β1 conditioned media.

In both cell types, collagen type I expression correlated with the gene expression of decorin. Furthermore, decorin seemed to be upregulated due to growth factor supplementation, indicating a catabolic state to collagenous matrix production. These results confirm the mediator role of decorin in the process of collagen fibrilogenesis and its involvement in the development of a tendon-like fibril profile.

Besides collagen type I, collagen type III is essential for normal fibrilogenesis and its regulation despite its smaller amounts in tendon tissues.

In our study, the collagen type III expression showed high values at the first time point in all media (Figure 3). The highest levels were observed in basic medium (A), followed by culture medium supplemented with PDGF-BB, TGF-β1, EGF and bFGF. Collagen type III relative expression decreased by 14 days, increasing again by 21 days for hAFSCs cultured in basic (A), EGF and bFGF media. Although these values were maintained in basic medium (A) at 28 days, they decreased in all other culture conditions. Conversely, collagen type III expression of hASCs maintained a basic level during the entire experiment, and was not particularly influenced by the cell culturing conditions.

### Tenascin C

The expression of tenascin C was maintained low in hAFSCs up to 21 days in culture (Figure 3). Then, a pick in the expression level was observed in media supplemented with EGF and bFGF. Tenascin C relative expression in hASCs was high by 7 days in culture, registering the highest values found in EGF supplemented medium. The exception was made for cells cultured in basic medium (B). After 2 weeks in culture, the expression decreased for all but TGF-β1 culture medium, and increased to the highest experimental values by 21 days in all conditioned media.

Overall, tenascin C was highly expressed after 21 days in culture, being more homogeneously expressed in hASCs. On the other hand, hAFSCs showed a peak of tenascin C only in EGF and bFGF supplemented media.

### Scleraxis

Scleraxis is a transcription factor expressed in the progenitors and tendon tissue cells. In our study, scleraxis was upregulated by 7 days in basic (A) and EGF media and tended to increase in EGF supplemented medium by 14 and 21 days in hAFSCs. A peak was reached in bFGF medium by 21 days. Our results in hAFSCs are justified by Reed and Johnson work on adipose and umbilical cord blood stem cells [26]. These studies indicate that FGF signal is necessary and sufficient for scleraxis expression of a tendon progenitor cell population.

In hASCs, the scleraxis genetic expression was increased at day 7, especially in TGF-β1 medium, and overall culture media by day 21. These results are also supported by the literature since TGF-β signaling is a potent inducer of scleraxis in cultured cells [3].

Human AFSCs and hASCs expressed all tendon-associated genes studied, with increased expression values by 21 days in culture.

The expression of the genetic markers had a dissimilar pattern in hAFSCs and hASCs, indicating that these stem cell populations respond differently to the different GFs. The expression of tendon-related genes of hAFSCs seemed to be mainly influenced by EGF and bFGF media while hASCs were more influenced by EGF and PDGF-BB. Interesting is the fact that EGF participated in stimulating the genetic expression of both hAFSCs and hASCs but in a distinctive way; EGF influenced the expression of tenascin C in hAFSCs and collagen type III expression in hASCs. Thus, the genetic expression towards a tenogenic lineage commitment may be influenced by stem cell origin, as cell origin may condition the stem cell response to local biochemical environment, i.e. the media composition.

## General Discussion

Stem cell differentiation in general and tenogenic differentiation in particular, are modulated by several biomolecules, including growth factors presented in a highly defined and tunable micro-environment. These biomolecules are expected to establish cell-to-cell contact and interact with intracellular signaling molecules as a responsive behavior to the extracellular milieu. The communication is likely to result in gene expression regulation, and ultimately on the synthesis of proteins to promote cell mechanisms that conduct to cell adjustments towards the external stimuli provided.

Since tendon associated markers are also found in other tissues and cells, a combination of these markers may provide some insight into the *in vitro* tenogenesis or tenogenic differentiation. The presence of tendon-related ECM proteins namely Tenascin C, Collagen I and Collagen III, described to have a key role in healthy functional tendons, were detected in both stem cells.

The growth factors (GFs) were selected based on recent publications, which associated these factors with tendon development and healing mechanisms [17]. The main goal of the present study was to understand how these GFs influence the tenogenic differentiation of the two stem cell sources, so as to eventually help defining an appropriate cell culture medium for inducing cells into tenogenic lineage. Although the GFs studied clearly influenced the stem cell response in terms of ECM production, none of these GFs evidenced a distinctive action in the synthesis of tendon-related proteins. Interestingly, the fluorescence signals for Tenascin C and Collagen I protein are detected in culture conditions where the genetic expression is low. We hypothesize that this effect may occur due to the timeline translational determinants that take place intracellularly from the upregulation of the interest gene to the protein translation, and transport to the extracellular matrix being produced. These results emphasize the complex dynamic of the GFs in cell processes and environmental interactions that take place in promoting cell differentiation *in vitro*.

Both hAFSCs and hASCs showed a longitudinal organization of actin fibers within cell rows as well as alignment of collagen fibers, suggesting that these are important morphological features to investigate for a deeper understanding of the unique orientation of the collagen fibrils in tendons.

Despite variations in the genetic expression levels of tendon-associated markers, the GFs studied do not seem to be essential for the biochemical stimulation of *in vitro* tenogenesis, as Tenascin C and Collagen I were also observed in basic medium. It is likely that stem cells studied may respond more promptly to mechanical stimulation rather than to biochemical signals provided by the culture medium.

Although pronounced conclusions were not achieved, the study demonstrates that some growth factors have a greater effect in a particular cell source than another, despite the fact that all growth factors studied are associated to tendon healing mechanisms.

Since tendon is a mechanoresponsive tissue, appropriate mechanical loads at physiological levels are usually beneficial to tendons in terms of enhancing the mechanical properties of the tendon [33], and achieving a healthy and functional tissue. Tendon tissues require specific micro-environments according to their function and correspondent anatomical regions. Therefore we speculate that tenogenic differentiation of hASCs and hAFSCs cultured in basal medium could be enhanced or accelerated in the presence of an external mechanical stimulus.

The selection of the stem cell source seems to be relevant in designing a tendon regeneration strategy. Despite the fact that both hAFSCs and hASCs did follow a tenogenic pathway, the cell responses to exogenous GFs stimulation were distinct, especially the ones found at a molecular biology level. These results indicate that the interactions occurring between these cells and the biochemical milieu are complex and intrincated and may be dependent on stem cell origin. These data point out the importance of understanding the cellular and molecular events induced by factors regulating stem cell proliferation and the consequences on their cellular influence.

In summary: results on cellular alignment morphology, immunolocalization and PCR analysis indicated that both stem cells can be biochemically induced towards tenogenic features. The stimulation into tenogenic lineage was demonstrated by the elongated and aligned cell morphology, the presence of a Tenascin C and Collagen I-rich ECM and the expression of gene markers typically associated to tendon tissues. Despite variations found between hAFSCs and hASCs responses to the GFs supplemented to the culture medium, the results further validate the potential of using adipose tissue and amniotic fluid as stem cell sources for tendon tissue engineering.

### Conclusion

The concept of stem cell inclusion in tendon-related strategies increases the number of cells locally, stimulating tissue regeneration. Furthermore, the cellular commitment towards the tenogenic lineage will simultaneously supply the local milieu with growth factors and cytokines that could be important and lineage-oriented for tendon regeneration.

Despite the relevance already described in tendon healing mechanism, the growth factors evaluated in our study did not emphasize a particular outcome on the tenogenic process of hAFSCs and hASCs considering the synthesis of tenogenic ECM proteins. Nevertheless, EGF and bFGF as well as EGF and PDGF-BB do have a molecular influence on the tendon-related genetic expression of hAFSCs and hASCs, respectively, although their role in the process of tenogenic differentiation of stem cells is still unveiled.

The fact that stem cells harvested and expanded from different sources showed a distinct behavior to the GFs supplemented in the culture medium may indicate that the origin of these cells may also have an effect on the process of differentiation. Thus, the selection of a source of stem cells should be considered as a potential contribute for injury/defect-directed approaches in the regenerative medicine field.

These results also suggest that the tenogenic differentiation follow complex and elaborated pathways that may depend on multiple growth factors at precise chronological points and/or a combination of multifactorial stimuli from the highly specific and delicate tendon natural environment.

## Supporting Information

Figure S1 **Microscopic observation of hAFSCs cultured up to 28 days in different supplemented media.** Human AFSCs photographs were obtained from live cells collected weekly using a phase contrast microscope. Magnification: 100 x.
(TIF)

Figure S2 **Microscopic observation of hASCs cultured up to 28 days in different supplemented media.** Human ASCs photographs were obtained from live cells collected weekly using a phase contrast microscope. Magnification: 100 x.
(TIF)
