# Correction: Understanding the Role of Growth Factors in Modulating Stem Cell Tenogenesis

**DOI:** 10.1371/journal.pone.0303106

**Published:** 2024-05-01

**Authors:** Ana I. Gonçalves, Márcia T. Rodrigues, Sang-Jin Lee, Anthony Atala, James J. Yoo, Rui L. Reis, Manuela E. Gomes

Errors were made during the preparation of Fig 2 which resulted in the erroneous duplication of the following panels:

- hAFSC 14 days EGF (incorrect panel) and hAFSC 14 days TGF-β (correct panel)
- hAFSC 21 days medium B (incorrect panel) and hAFSC 21 days TGF-β (correct panel)
- hASC 21 days TGF-β (correct panel) and hASC 28 days medium A (incorrect panel)

With this correction, the authors provide a corrected version of Fig 2 as well as the original underlying representative images for all panels (S1 File below). The authors stated that most of the underlying data for this article was deleted in accordance with institutional policy. However, parts of the data for Fig 1, Fig 3, S1 Fig, and S2 Fig were retained on personal backup storage and are available on reasonable request. The authors apologize for the errors in the published article.

In reviewing this matter, the *PLOS ONE* Editors noted a potential conflict of interest between the authors and the original Academic Editor who handled the peer review of this article. Consequently, this article was subject to post-publication re-review by an independent member of the *PLOS ONE* Editorial Board, who verified the article and its findings. The *PLOS ONE* Editors apologize that this conflict was not identified prior to the article’s publication.

**Fig 2:**
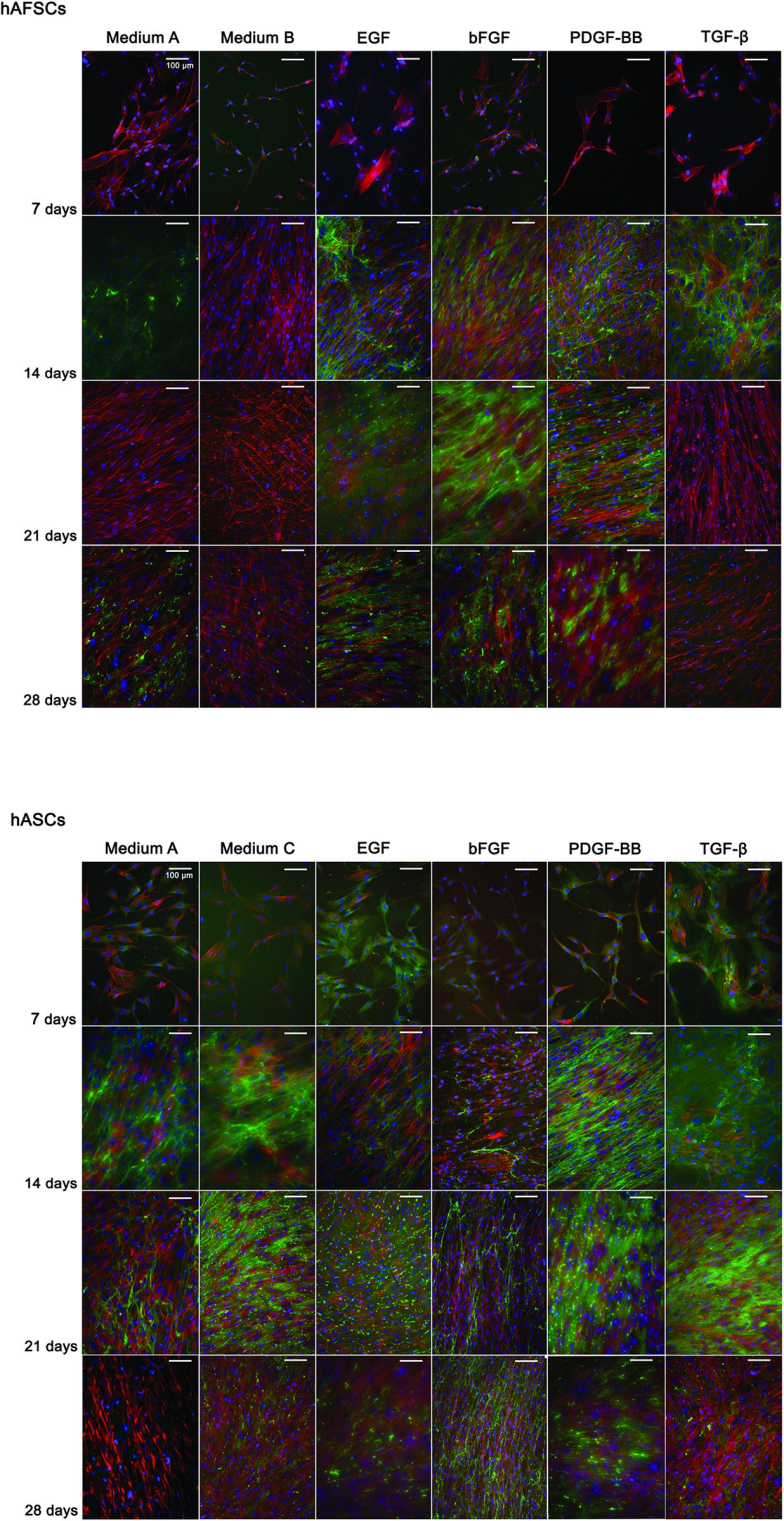
Tenascin C immunolocation in hAFSCs and hASCs cultured up to 28 days in different supplemented media. DAPI (blue)and phalloidin-conjugate (red) stain cell nucleus and cytoskeleton, respectively. Tenascin C is stained in green and represents a tendon ECM protein. Scale bar represents 100 µm. Magnification: 200 x.

## Supporting information

S1 File Fig 2 original underlying images. (ZIP)
