# Correction: Correction: Understanding the Role of Growth Factors in Modulating Stem Cell Tenogenesis

**DOI:** 10.1371/journal.pone.0304645

**Published:** 2024-05-24

**Authors:** The PLOS ONE Staff

One of the underlying images in S1 File is incorrectly labeled. An updated S1 File with the correctly labeled underlying image is provided here.

The authors and the publisher apologize for the error.

## Supporting information

S1 File Fig 2 original underlying images. (ZIP)
