# Supplementary figures and images for: Understanding the Role of Growth Factors in Modulating Stem Cell Tenogenesis
Source: PLoS One. 2013 Dec 30;8(12):e83734. doi: 10.1371/journal.pone.0083734 (PMC3875481; doi:10.1371/journal.pone.0083734)

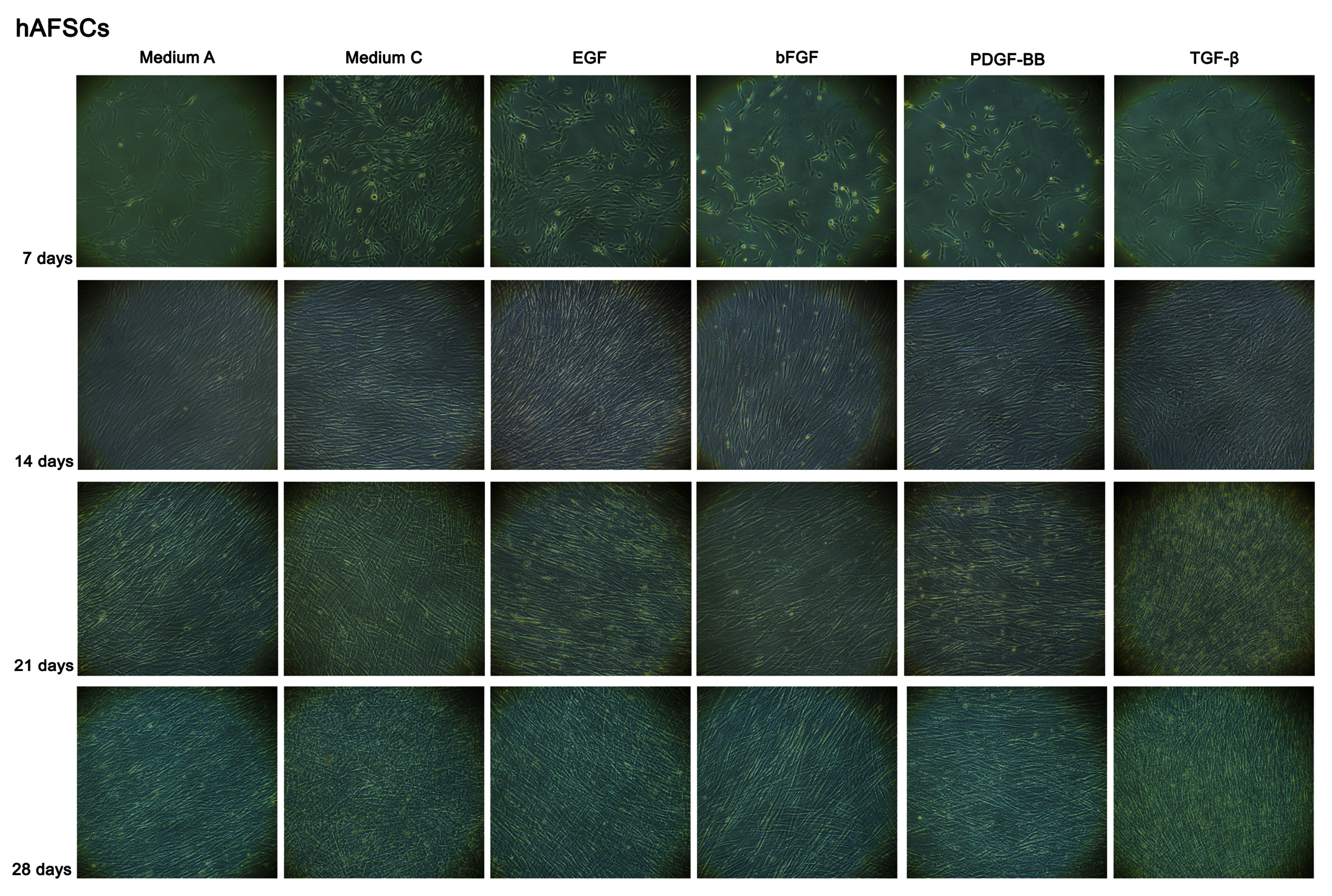

Supplement: Figure S1 — Microscopic observation of hAFSCs cultured up to 28 days in different supplemented media. Human AFSCs photographs were obtained from live cells collected weekly using a phase contrast microscope. Magnification: 100 x. (TIF) [file pone.0083734.s001.tif]

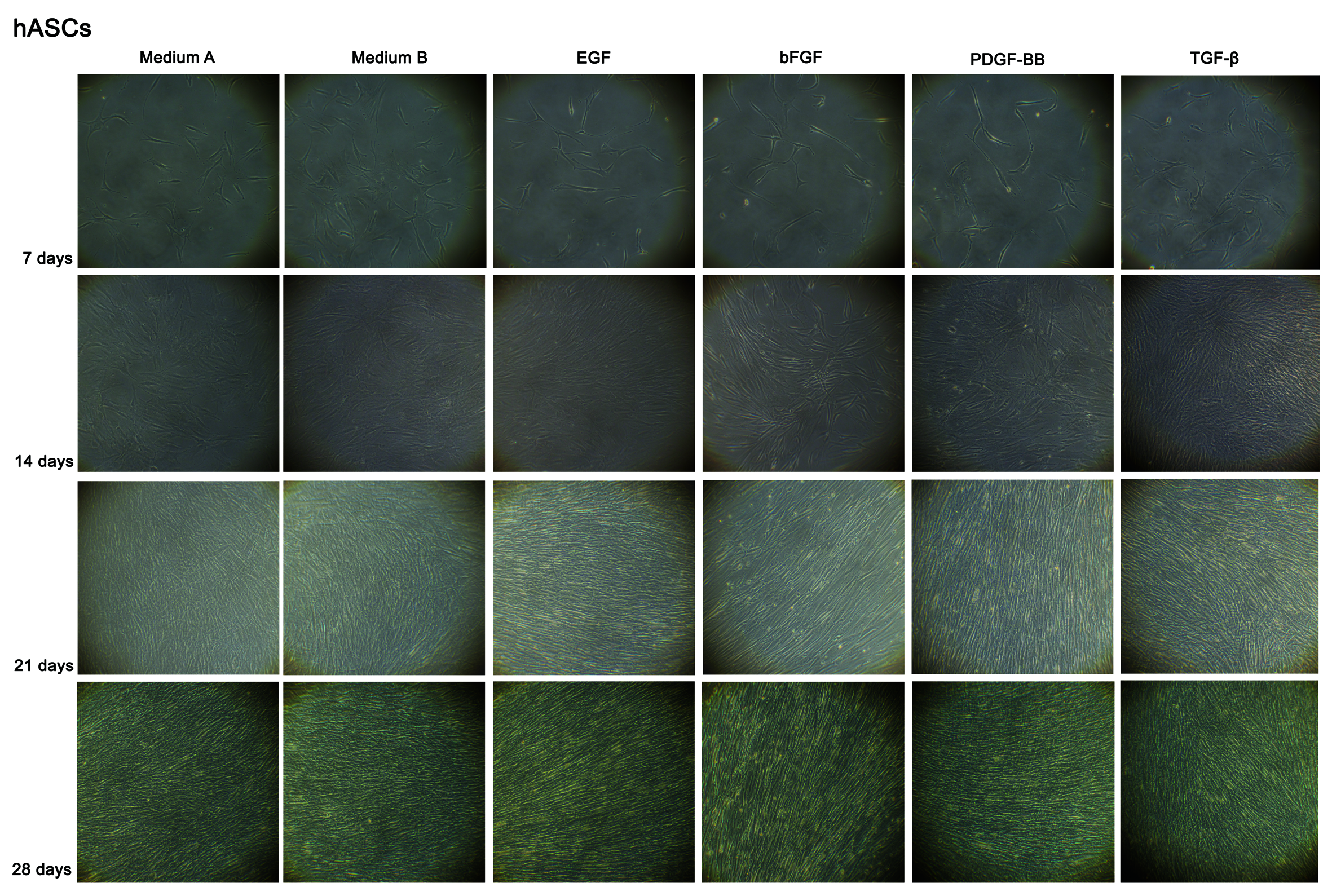

Supplement: Figure S2 — Microscopic observation of hASCs cultured up to 28 days in different supplemented media. Human ASCs photographs were obtained from live cells collected weekly using a phase contrast microscope. Magnification: 100 x. (TIF) [file pone.0083734.s002.tif]
