# Supplementary figures and images for: Correction: Understanding the Role of Growth Factors in Modulating Stem Cell Tenogenesis
Source: PLoS One. 2024 May 1;19(5):e0303106. doi: 10.1371/journal.pone.0303106 (PMC11062529; doi:10.1371/journal.pone.0303106)

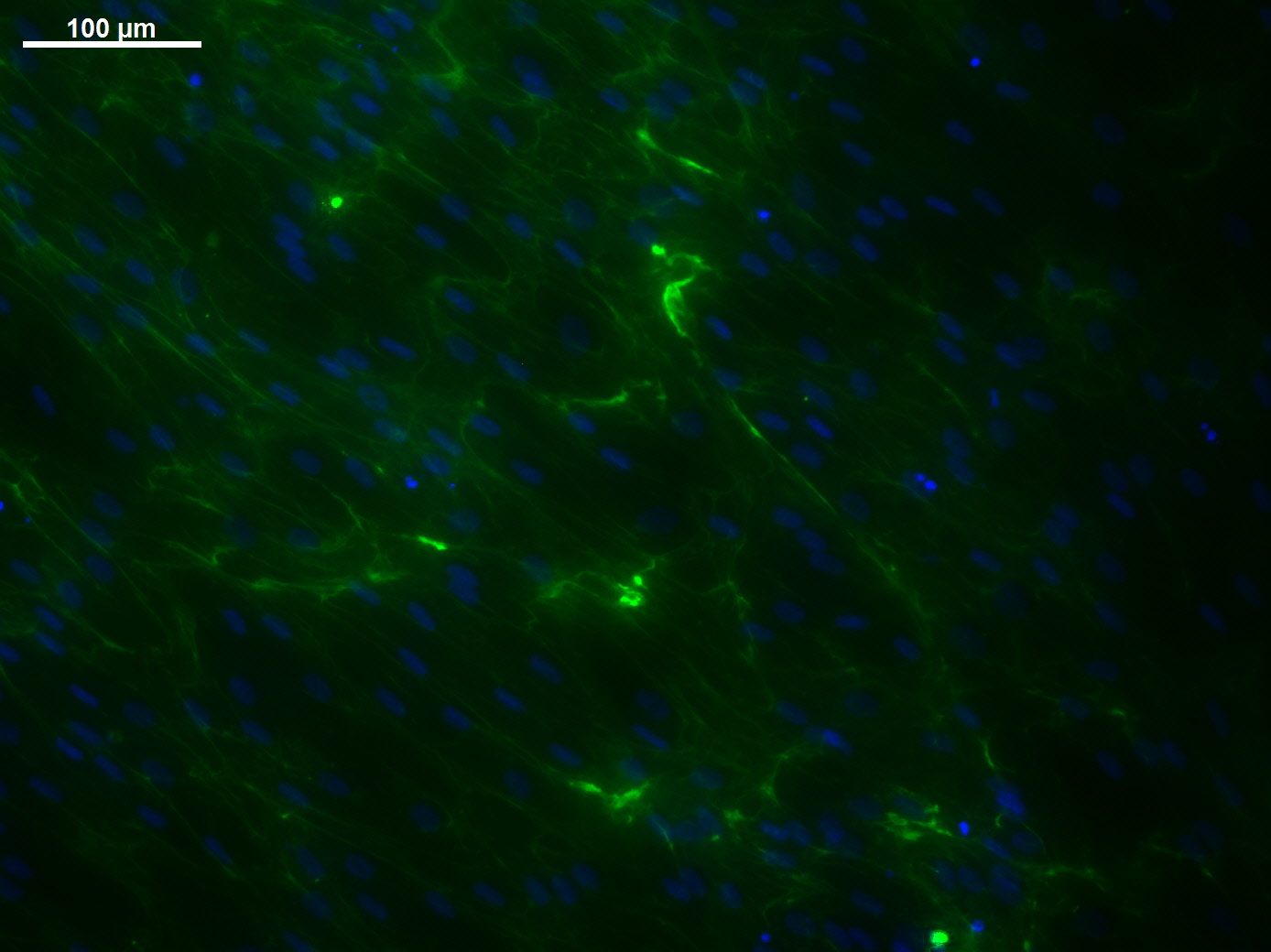

Supplement: S1 File — (ZIP) [file pone.0303106.s001.zip › S1 File/Figure2_TNC_a enviar/AFSCs_TNC_Fig2/14d_m1.JPG]

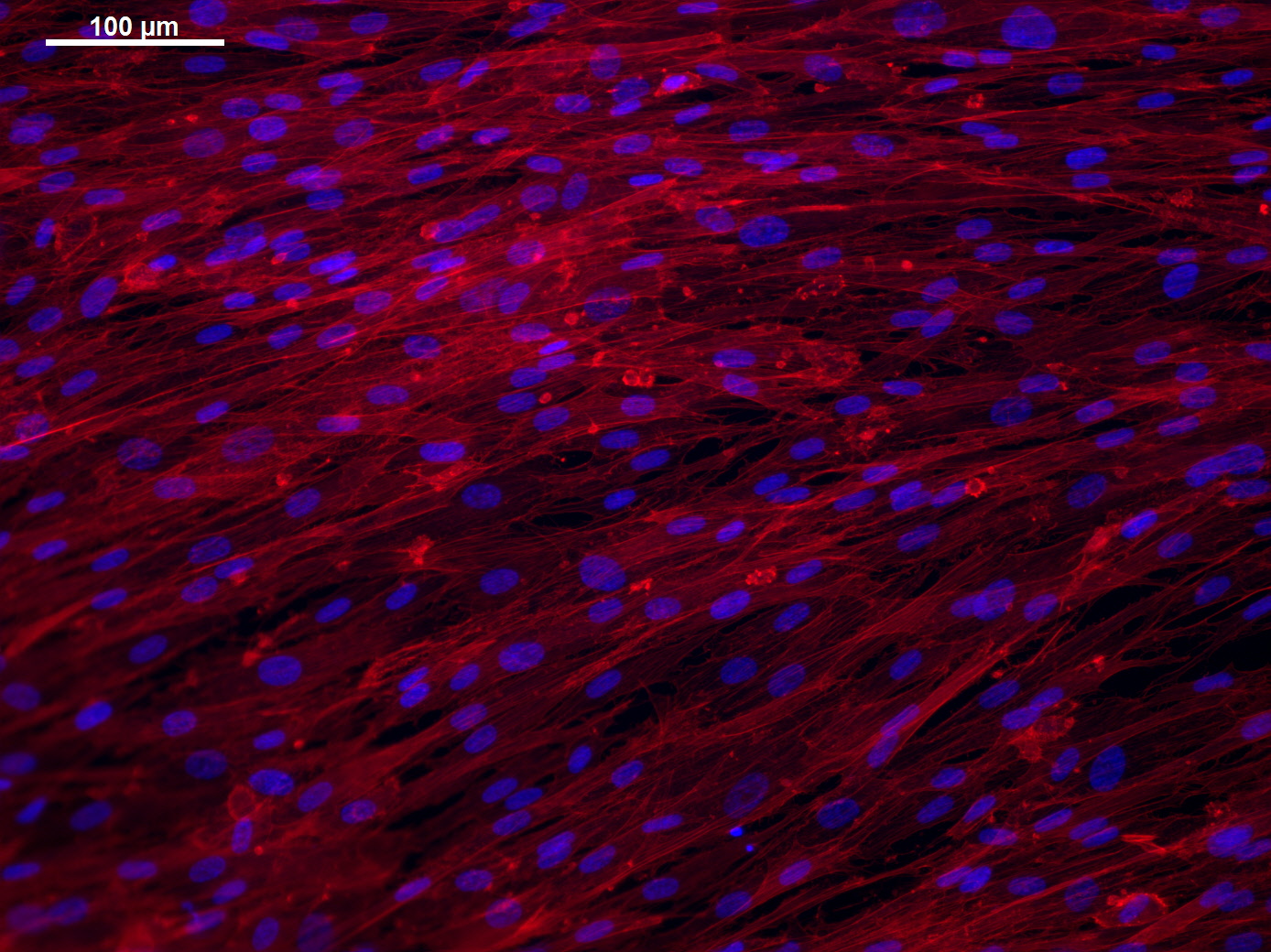

Supplement: S1 File — (ZIP) [file pone.0303106.s001.zip › S1 File/Figure2_TNC_a enviar/AFSCs_TNC_Fig2/14d_m2.JPG]

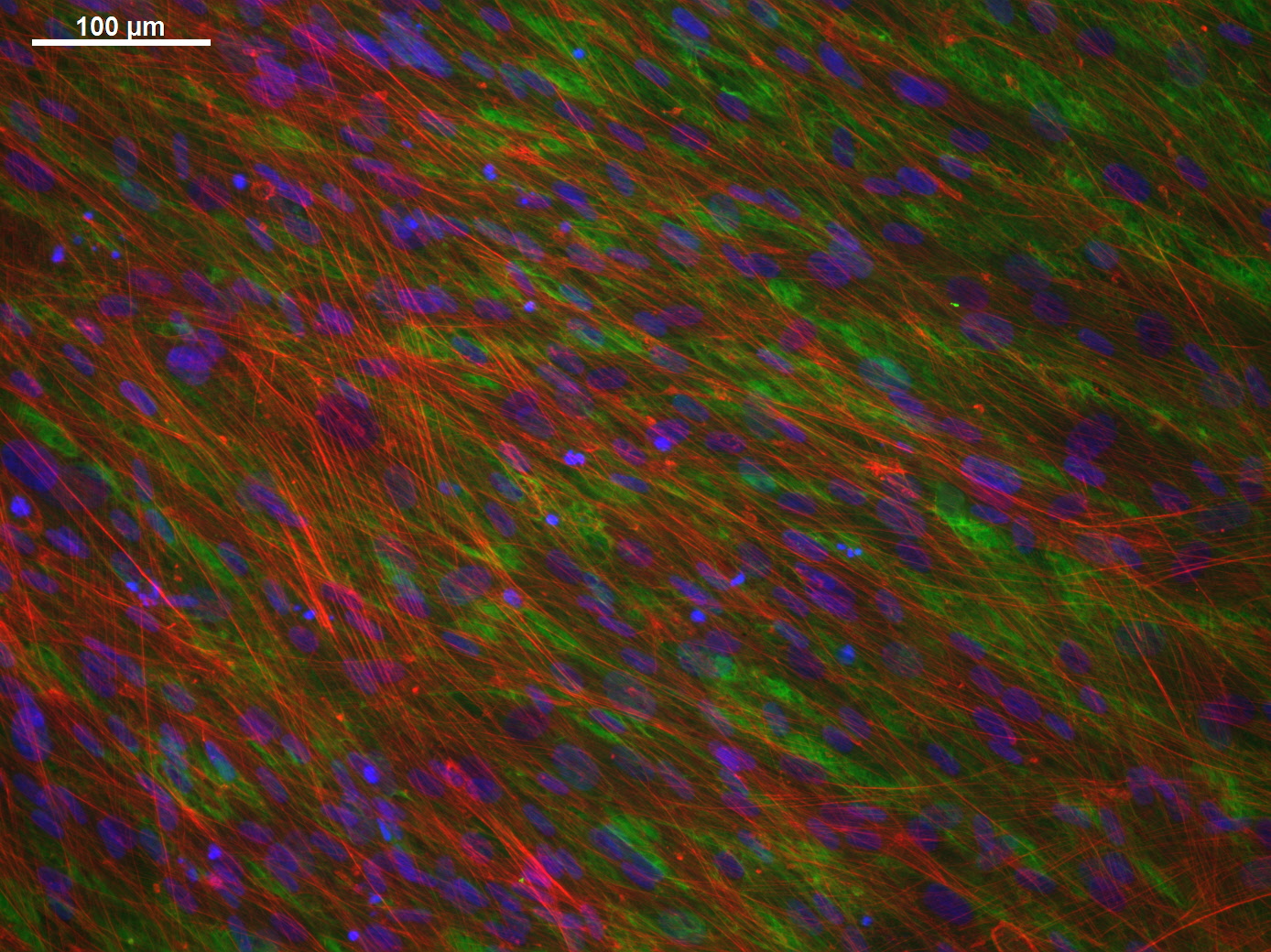

Supplement: S1 File — (ZIP) [file pone.0303106.s001.zip › S1 File/Figure2_TNC_a enviar/AFSCs_TNC_Fig2/14d_m3.JPG]

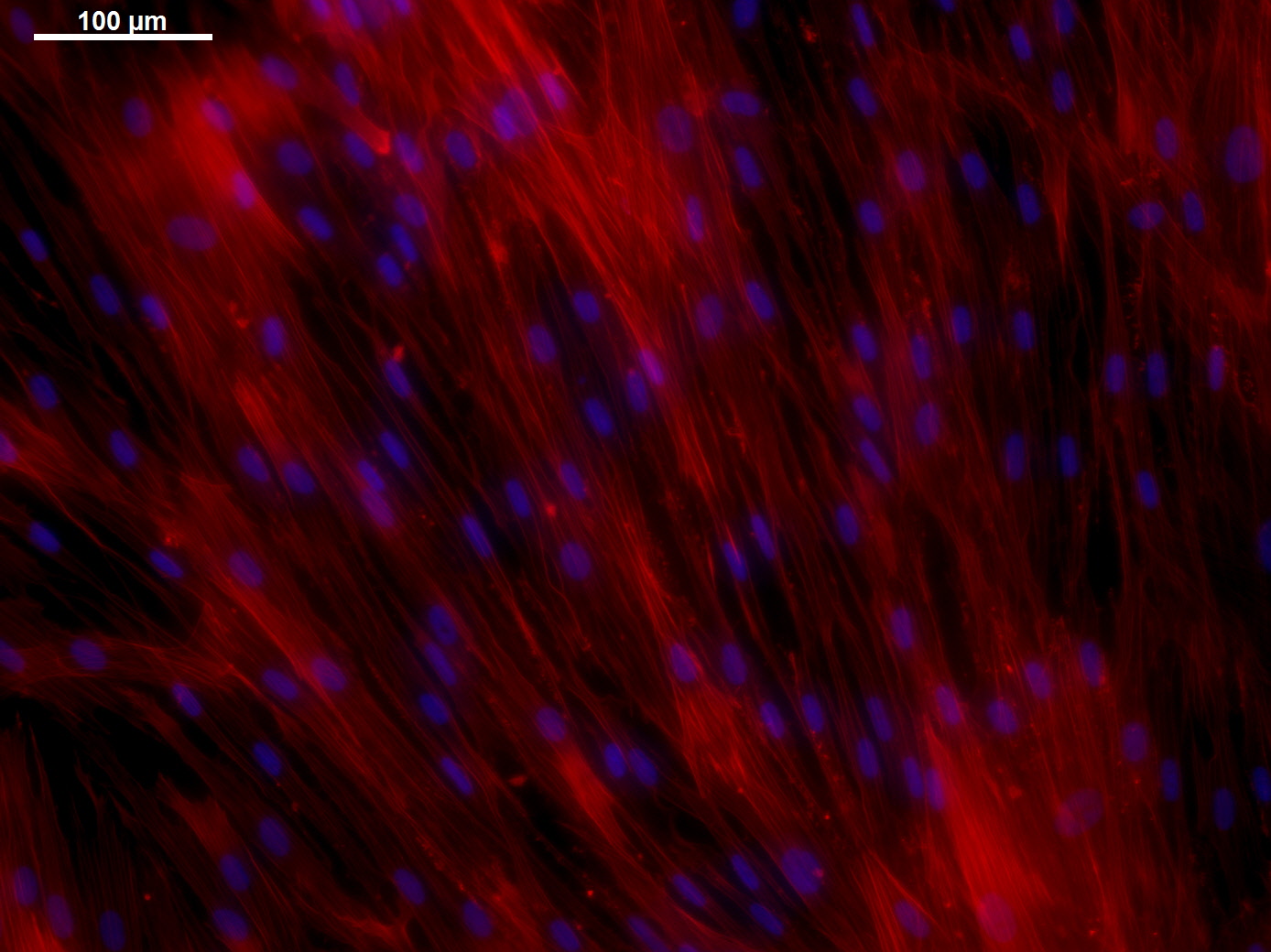

Supplement: S1 File — (ZIP) [file pone.0303106.s001.zip › S1 File/Figure2_TNC_a enviar/AFSCs_TNC_Fig2/14d_m4.JPG]

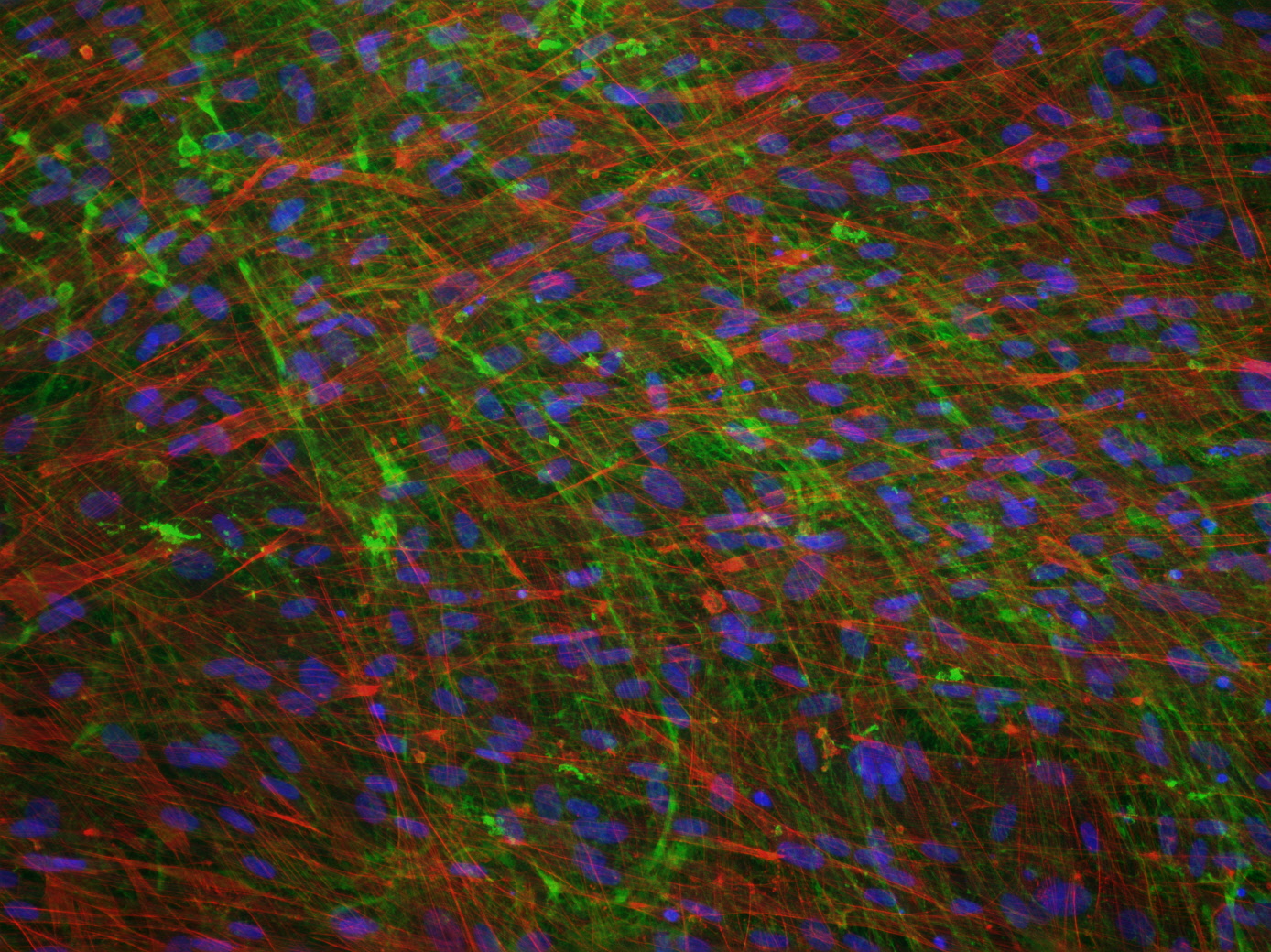

Supplement: S1 File — (ZIP) [file pone.0303106.s001.zip › S1 File/Figure2_TNC_a enviar/AFSCs_TNC_Fig2/14d_m5.JPG]

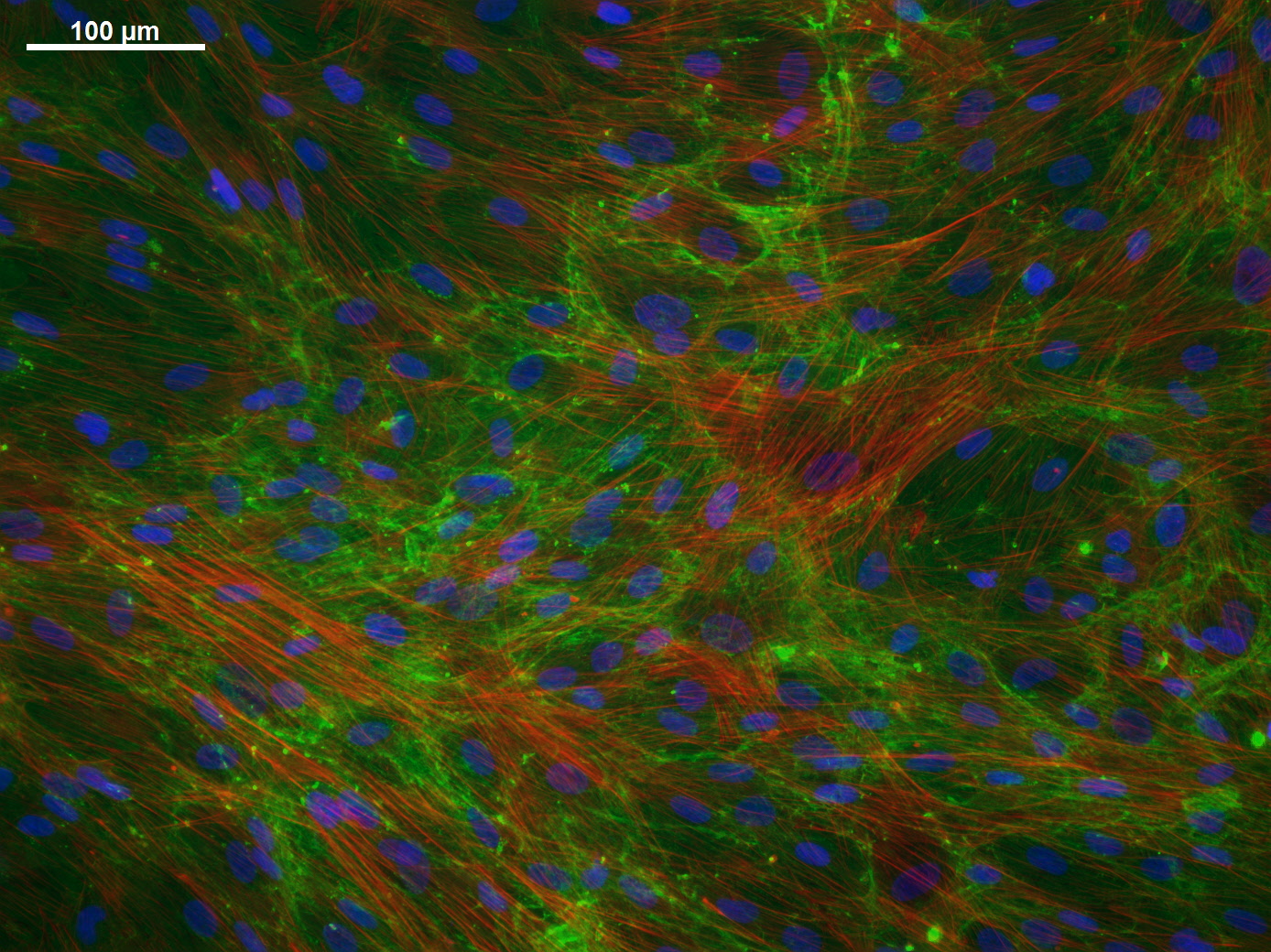

Supplement: S1 File — (ZIP) [file pone.0303106.s001.zip › S1 File/Figure2_TNC_a enviar/AFSCs_TNC_Fig2/14d_m6.JPG]

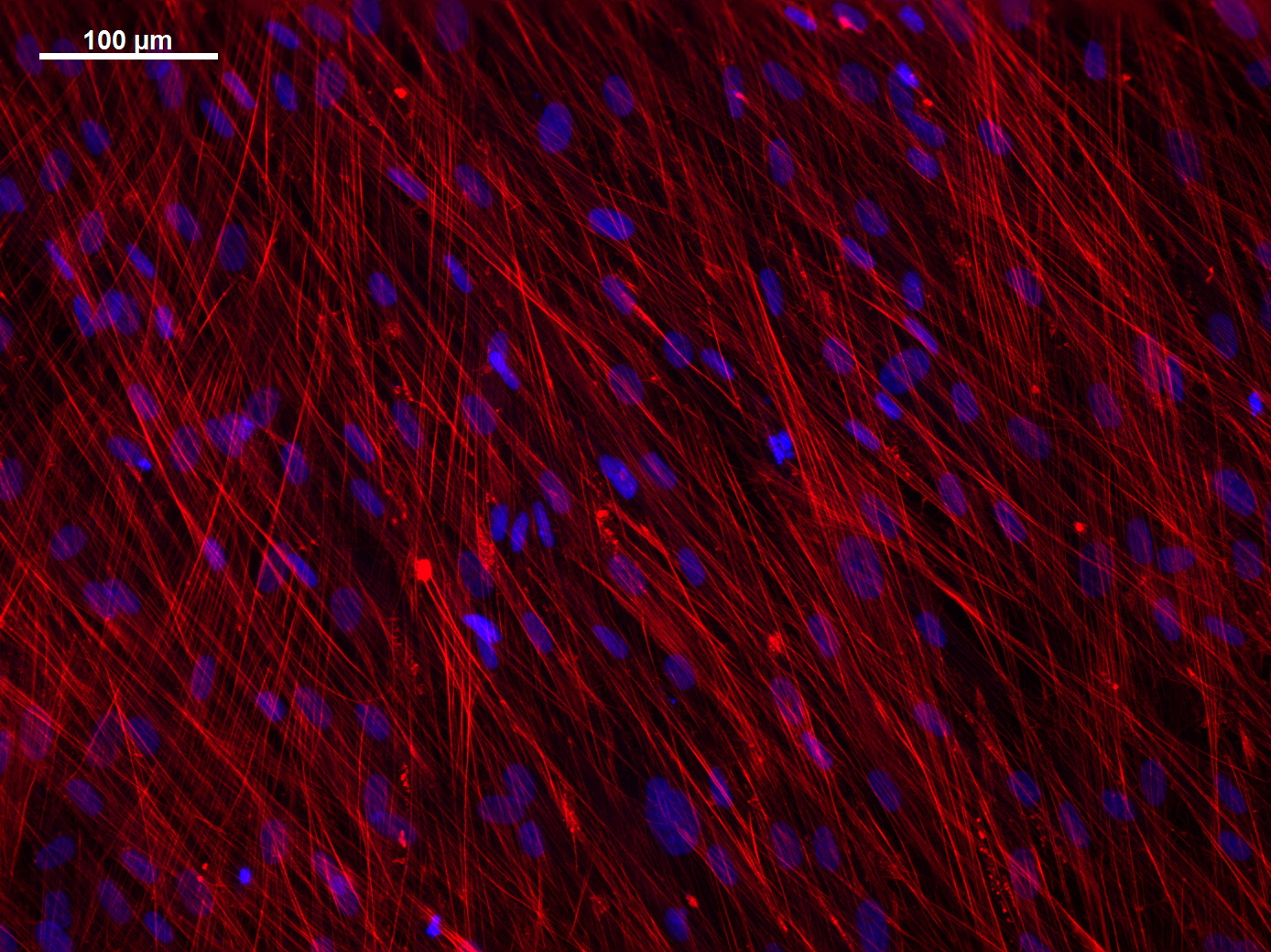

Supplement: S1 File — (ZIP) [file pone.0303106.s001.zip › S1 File/Figure2_TNC_a enviar/AFSCs_TNC_Fig2/21d_m1.JPG]

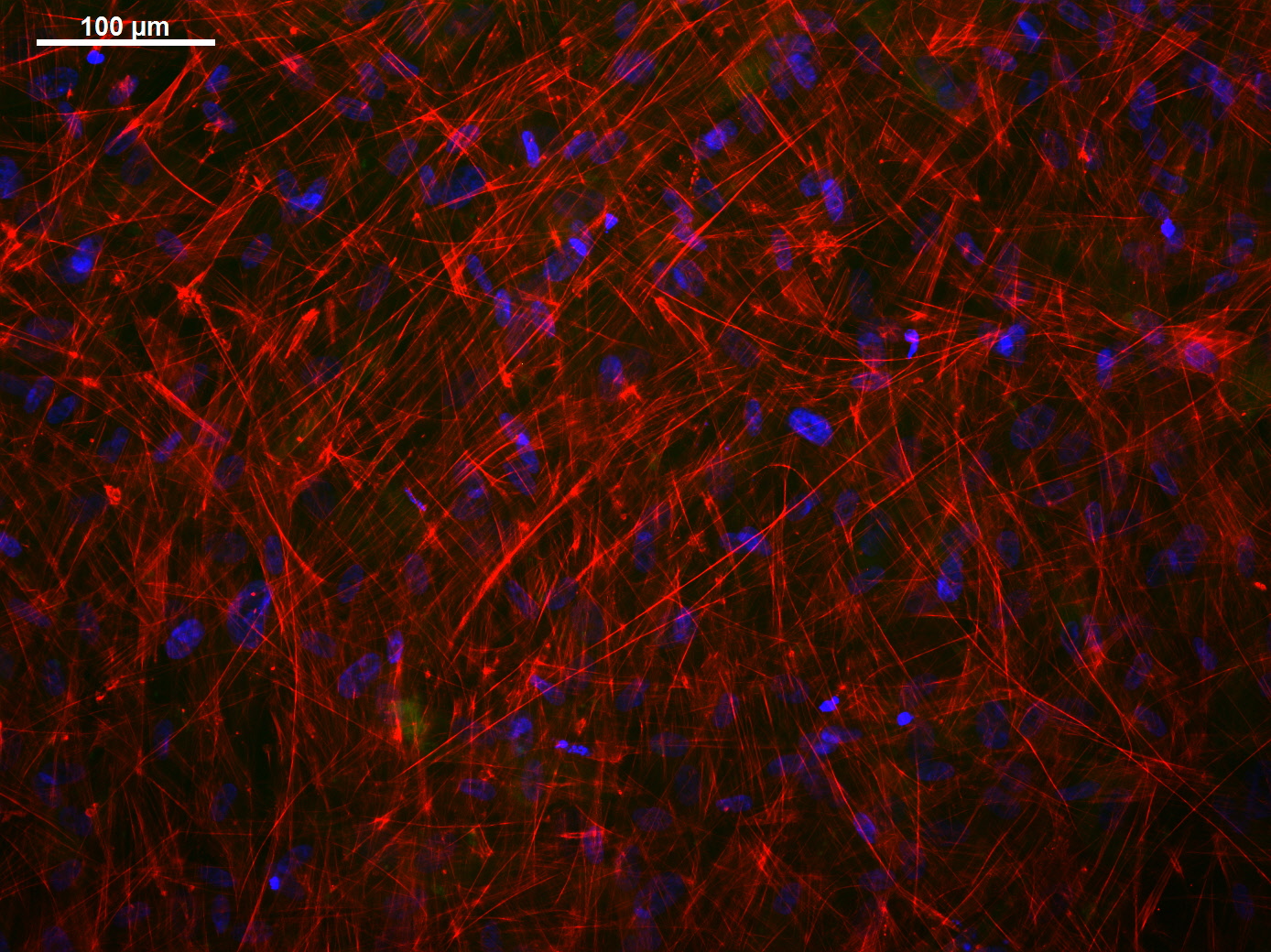

Supplement: S1 File — (ZIP) [file pone.0303106.s001.zip › S1 File/Figure2_TNC_a enviar/AFSCs_TNC_Fig2/21d_m2.JPG]

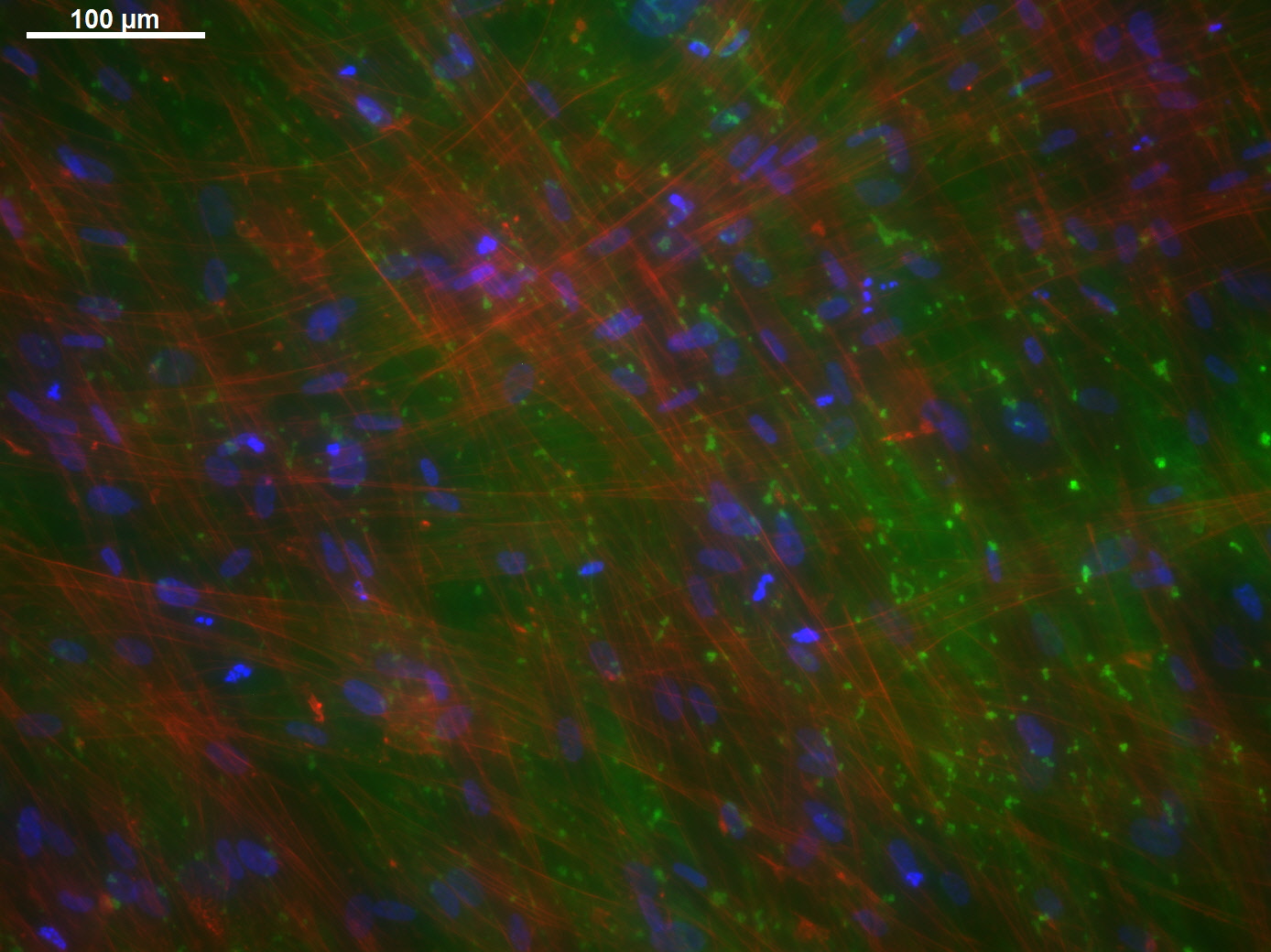

Supplement: S1 File — (ZIP) [file pone.0303106.s001.zip › S1 File/Figure2_TNC_a enviar/AFSCs_TNC_Fig2/21d_m3.JPG]

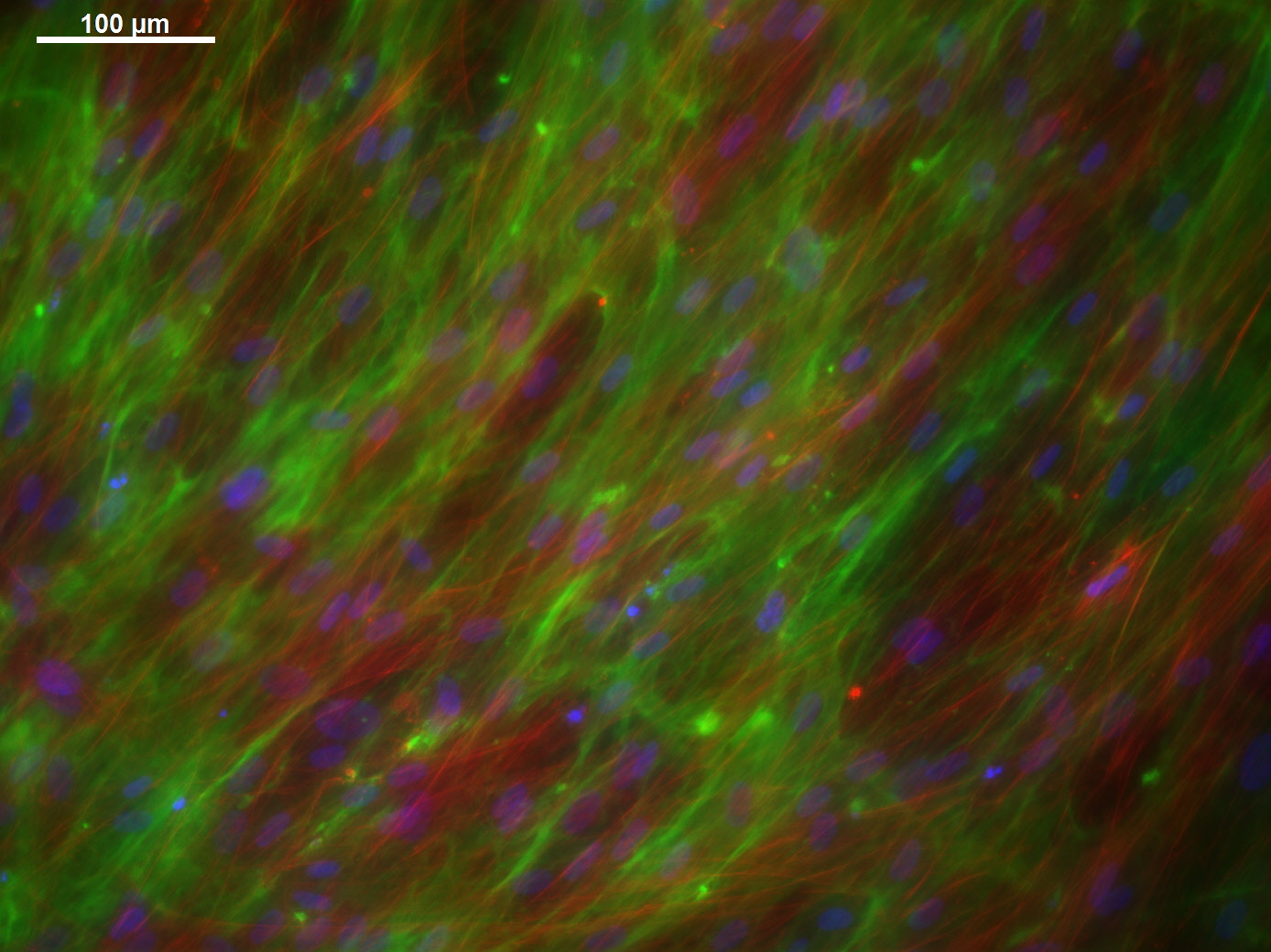

Supplement: S1 File — (ZIP) [file pone.0303106.s001.zip › S1 File/Figure2_TNC_a enviar/AFSCs_TNC_Fig2/21d_m4.JPG]

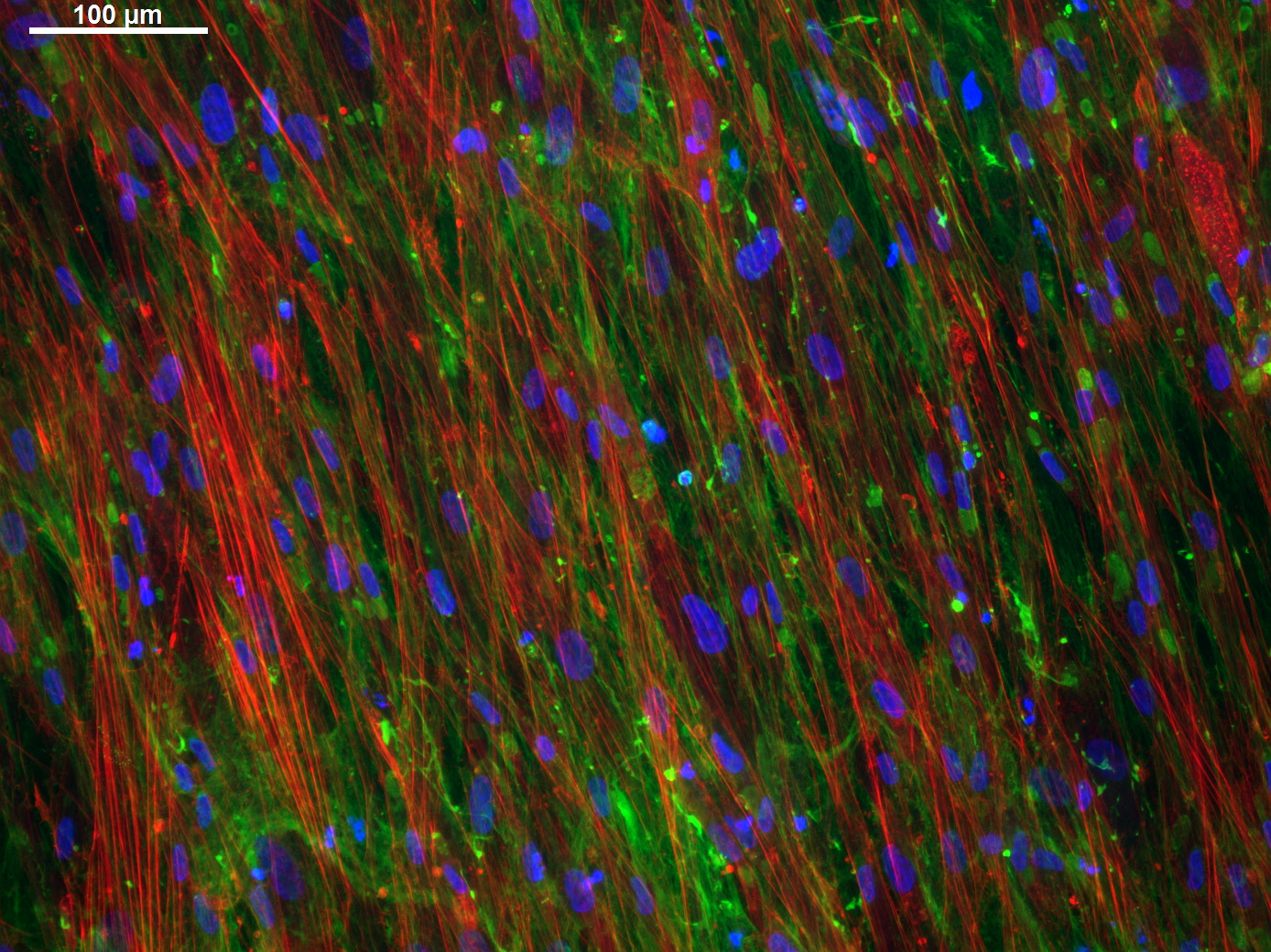

Supplement: S1 File — (ZIP) [file pone.0303106.s001.zip › S1 File/Figure2_TNC_a enviar/AFSCs_TNC_Fig2/21d_m5.JPG]

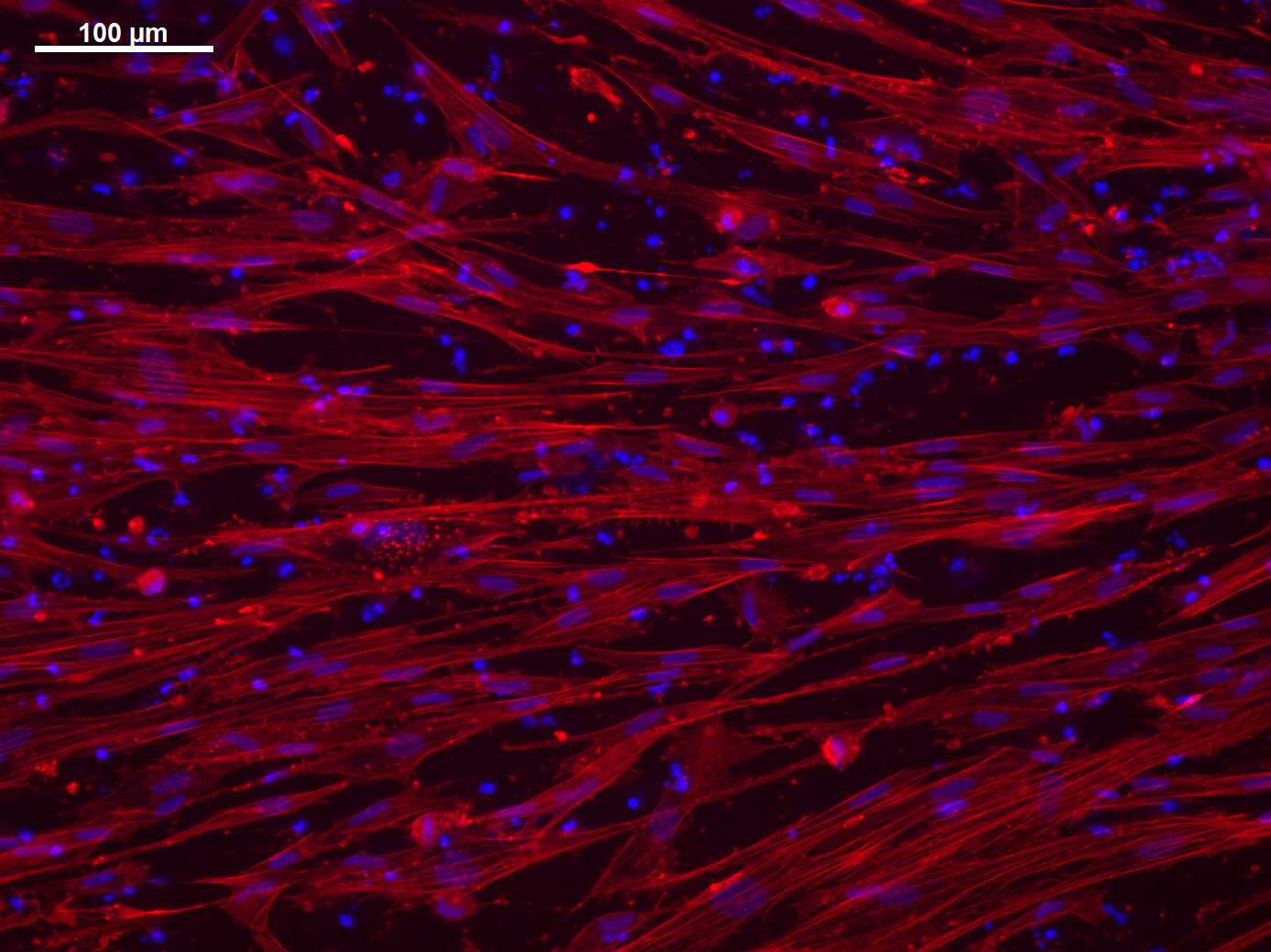

Supplement: S1 File — (ZIP) [file pone.0303106.s001.zip › S1 File/Figure2_TNC_a enviar/AFSCs_TNC_Fig2/21d_m6.JPG]

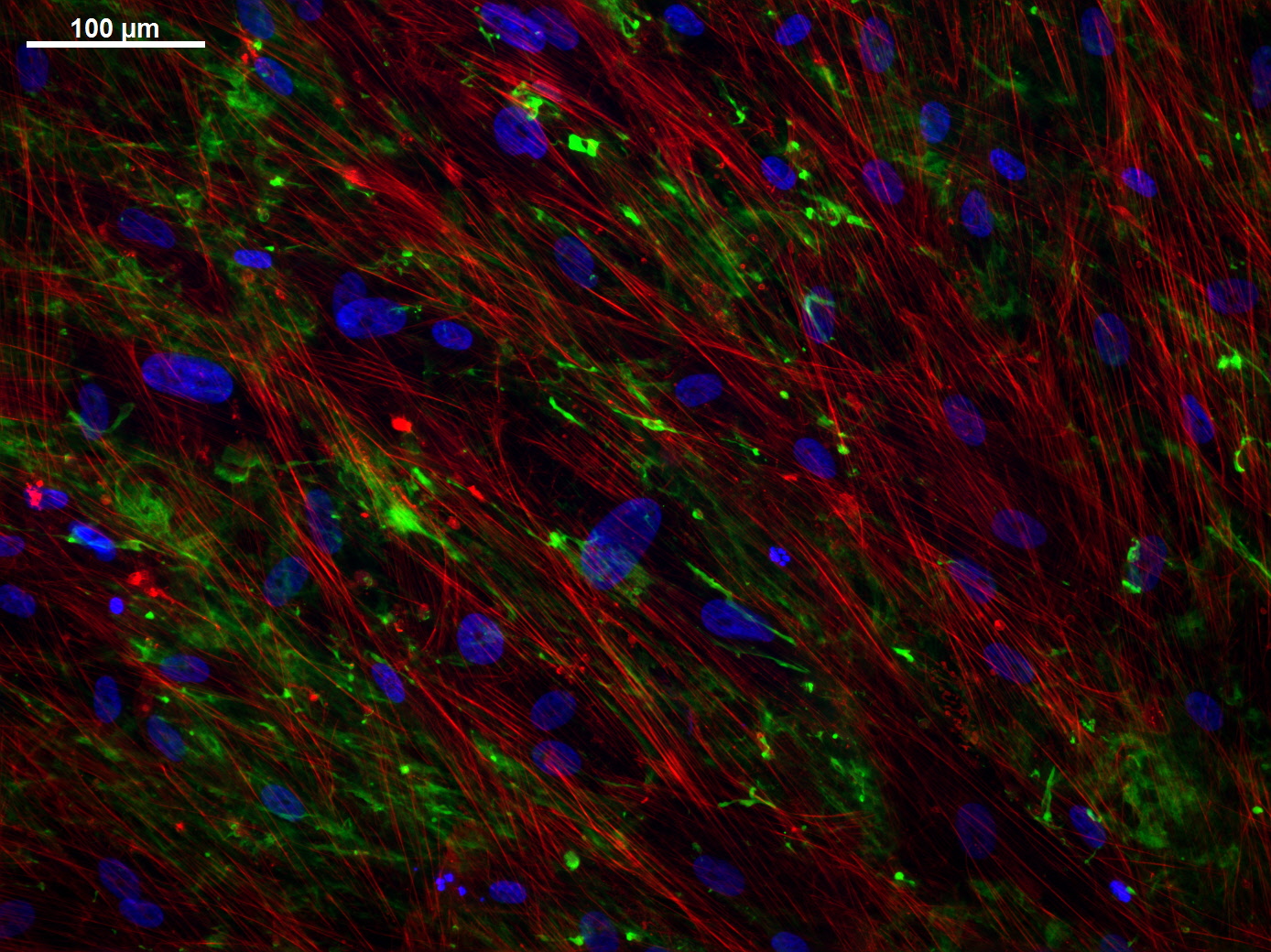

Supplement: S1 File — (ZIP) [file pone.0303106.s001.zip › S1 File/Figure2_TNC_a enviar/AFSCs_TNC_Fig2/28d_m1.JPG]

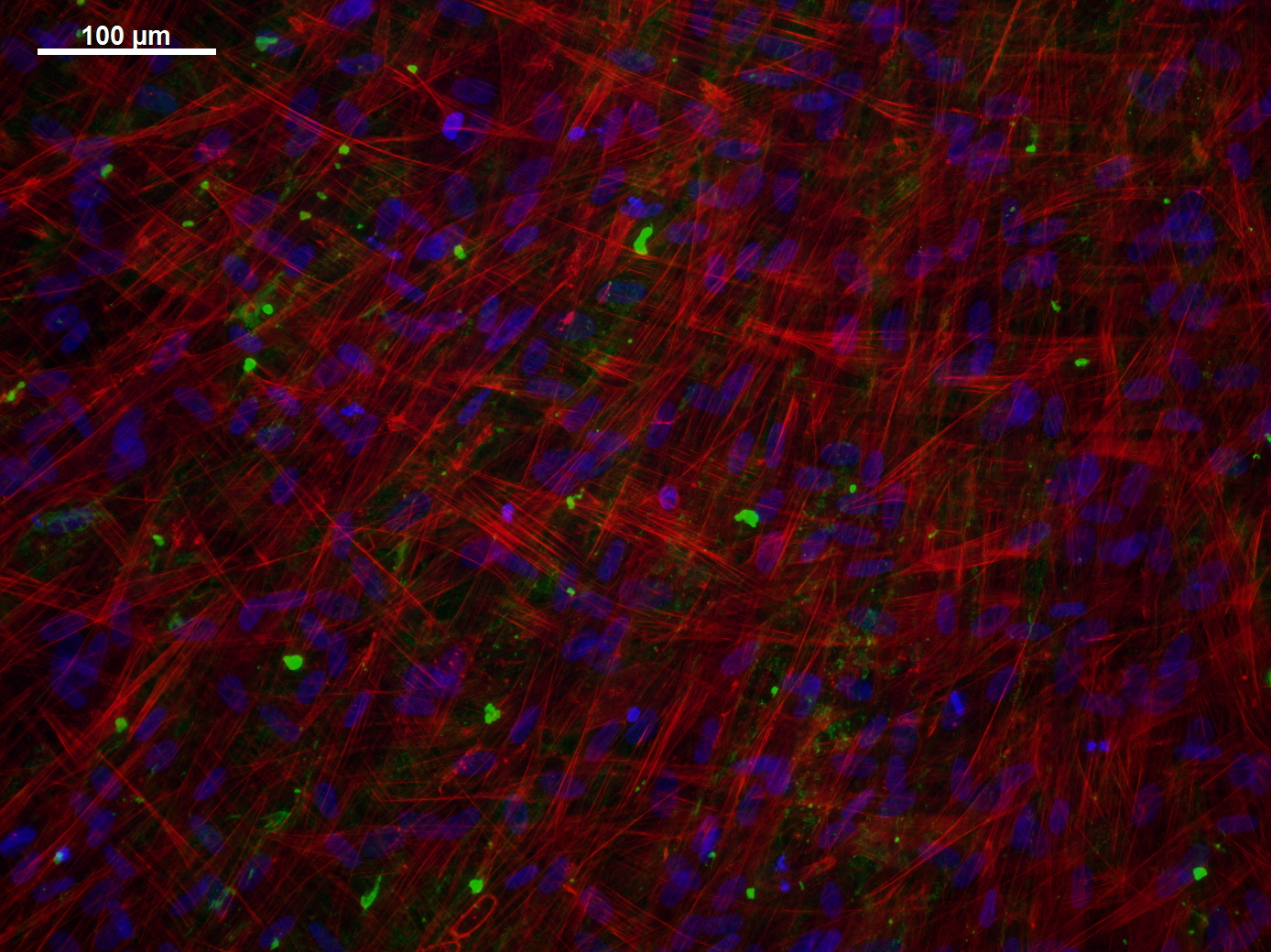

Supplement: S1 File — (ZIP) [file pone.0303106.s001.zip › S1 File/Figure2_TNC_a enviar/AFSCs_TNC_Fig2/28d_m2.JPG]

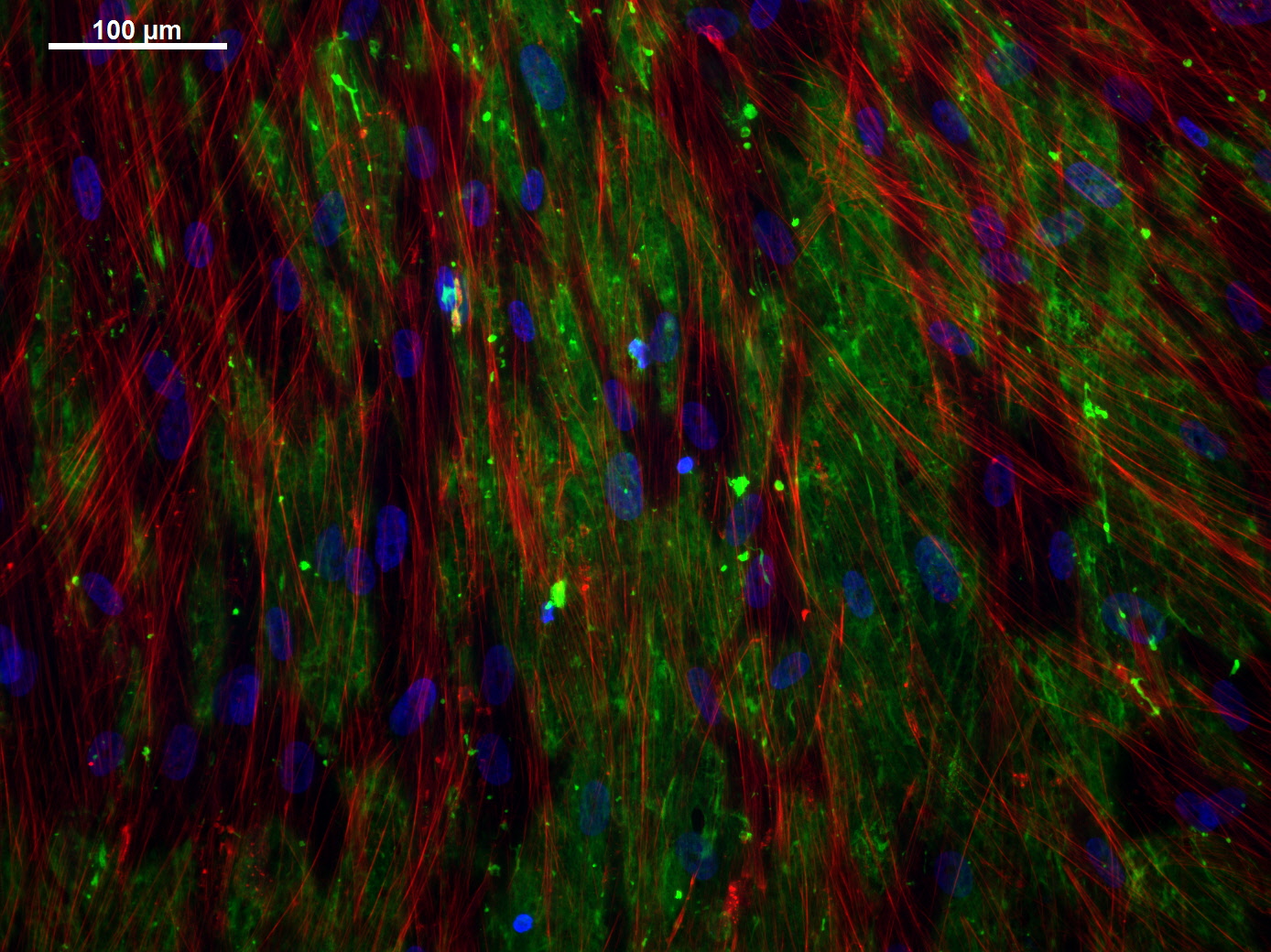

Supplement: S1 File — (ZIP) [file pone.0303106.s001.zip › S1 File/Figure2_TNC_a enviar/AFSCs_TNC_Fig2/28d_m3.JPG]

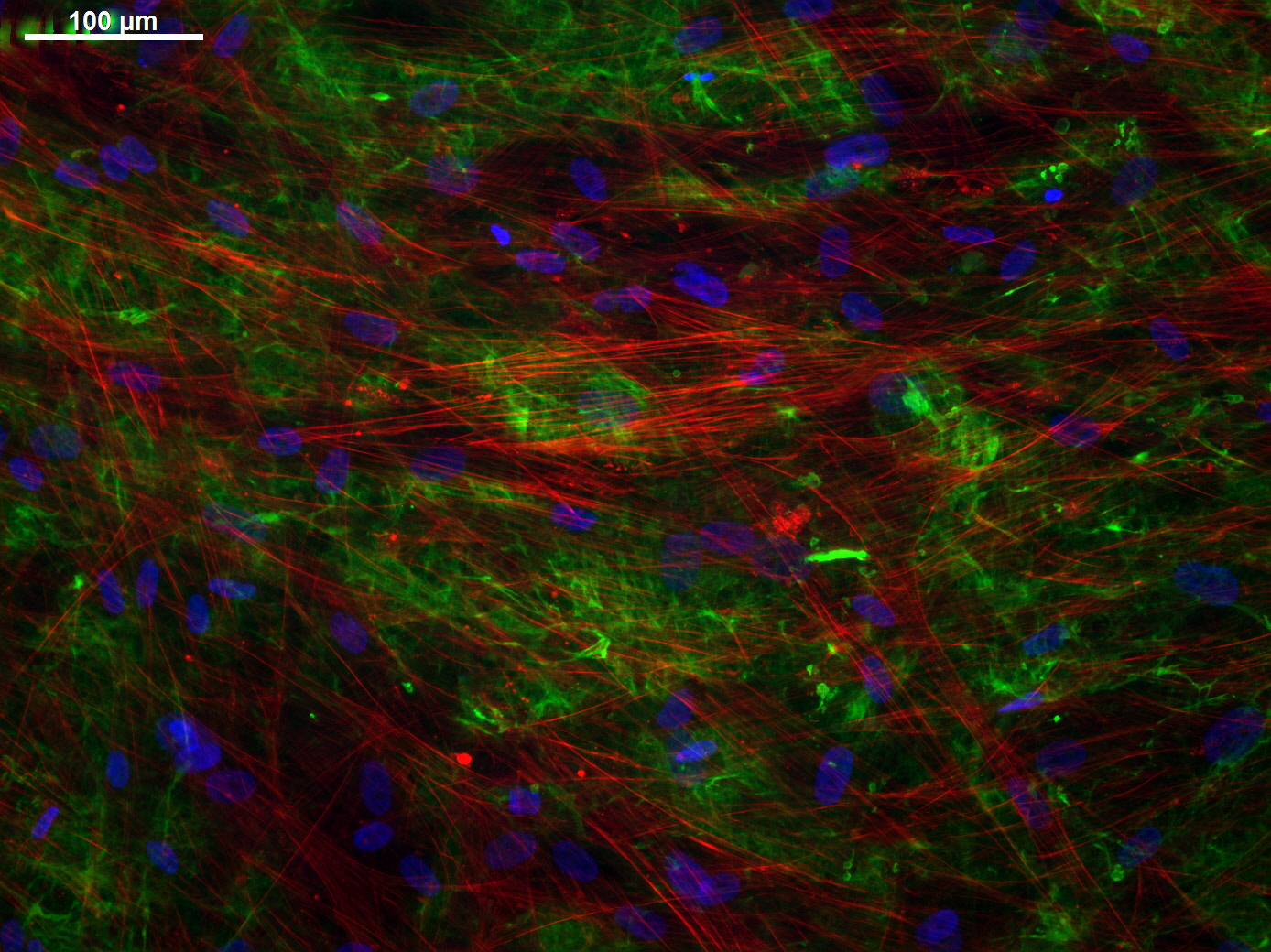

Supplement: S1 File — (ZIP) [file pone.0303106.s001.zip › S1 File/Figure2_TNC_a enviar/AFSCs_TNC_Fig2/28d_m4.JPG]

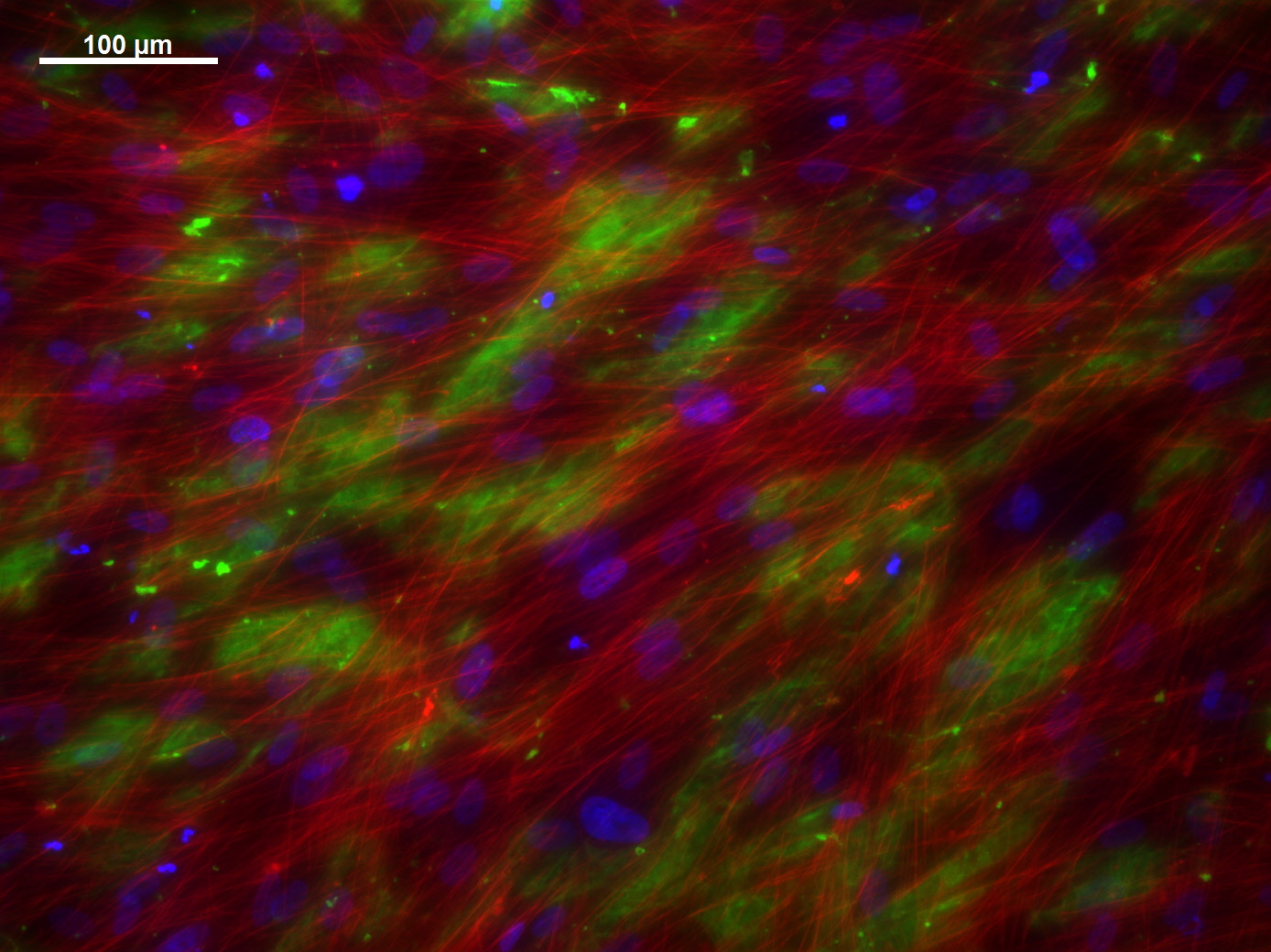

Supplement: S1 File — (ZIP) [file pone.0303106.s001.zip › S1 File/Figure2_TNC_a enviar/AFSCs_TNC_Fig2/28d_m5.JPG]

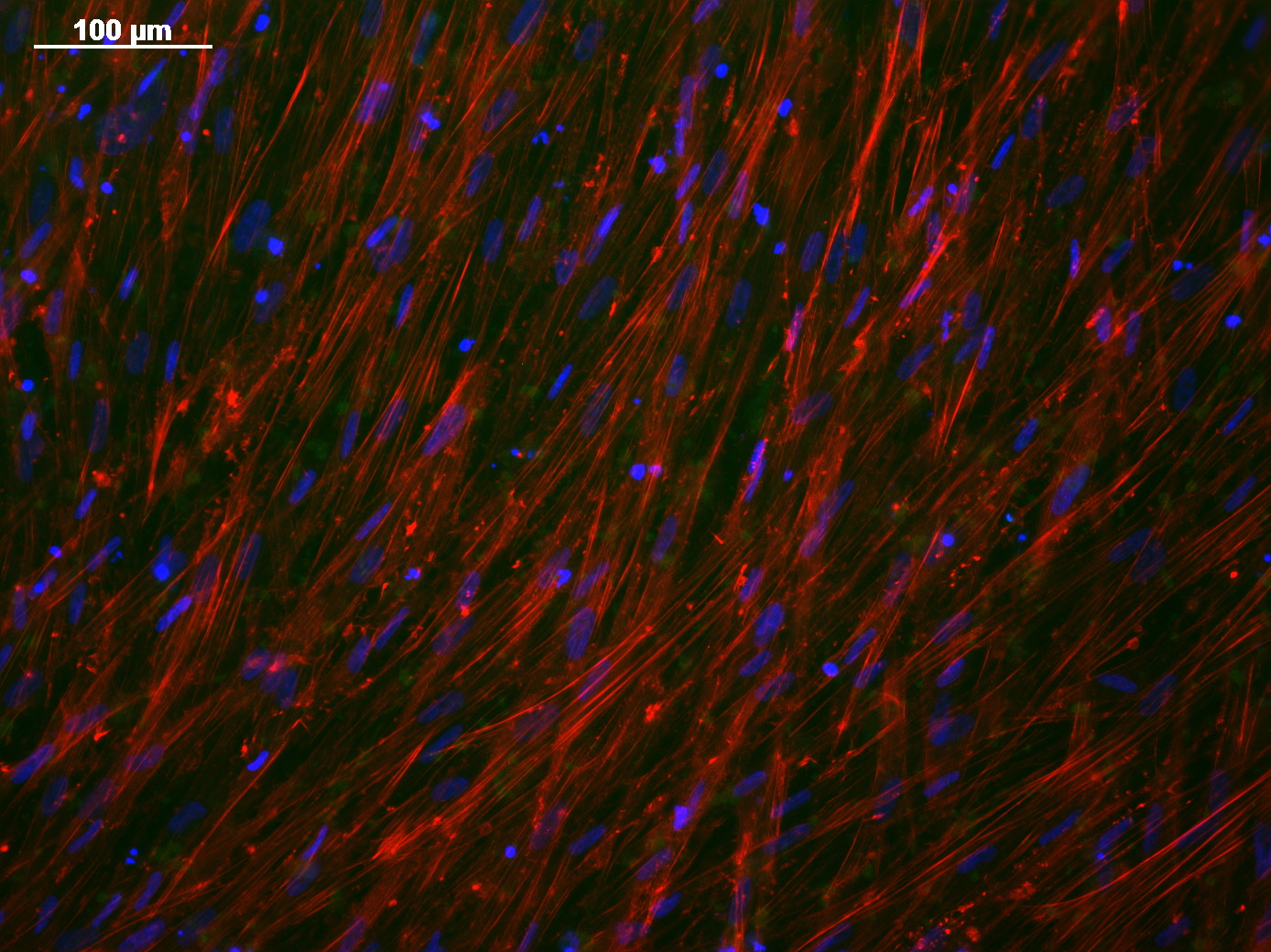

Supplement: S1 File — (ZIP) [file pone.0303106.s001.zip › S1 File/Figure2_TNC_a enviar/AFSCs_TNC_Fig2/28d_m6.jpg]

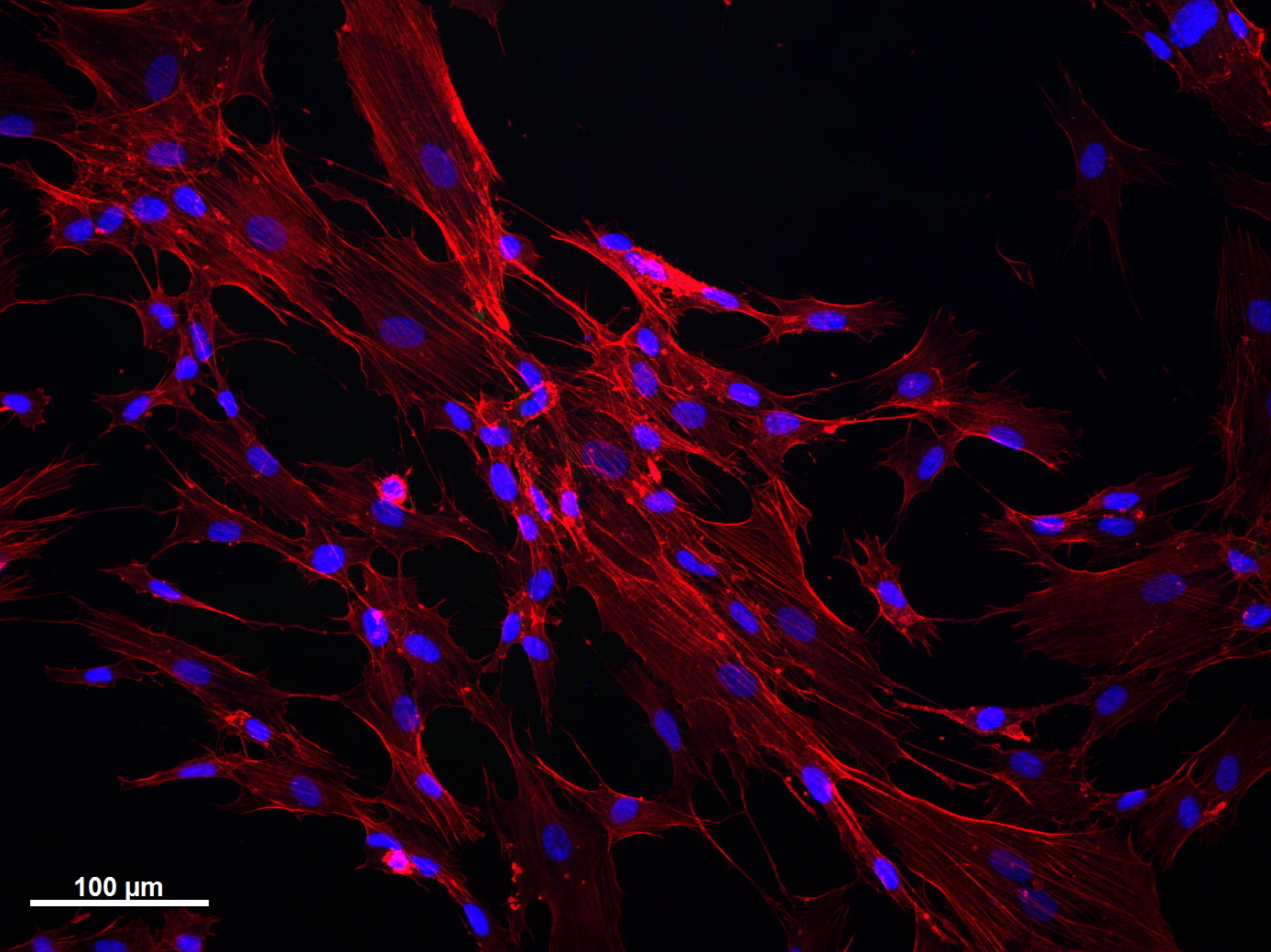

Supplement: S1 File — (ZIP) [file pone.0303106.s001.zip › S1 File/Figure2_TNC_a enviar/AFSCs_TNC_Fig2/7d_m1.JPG]

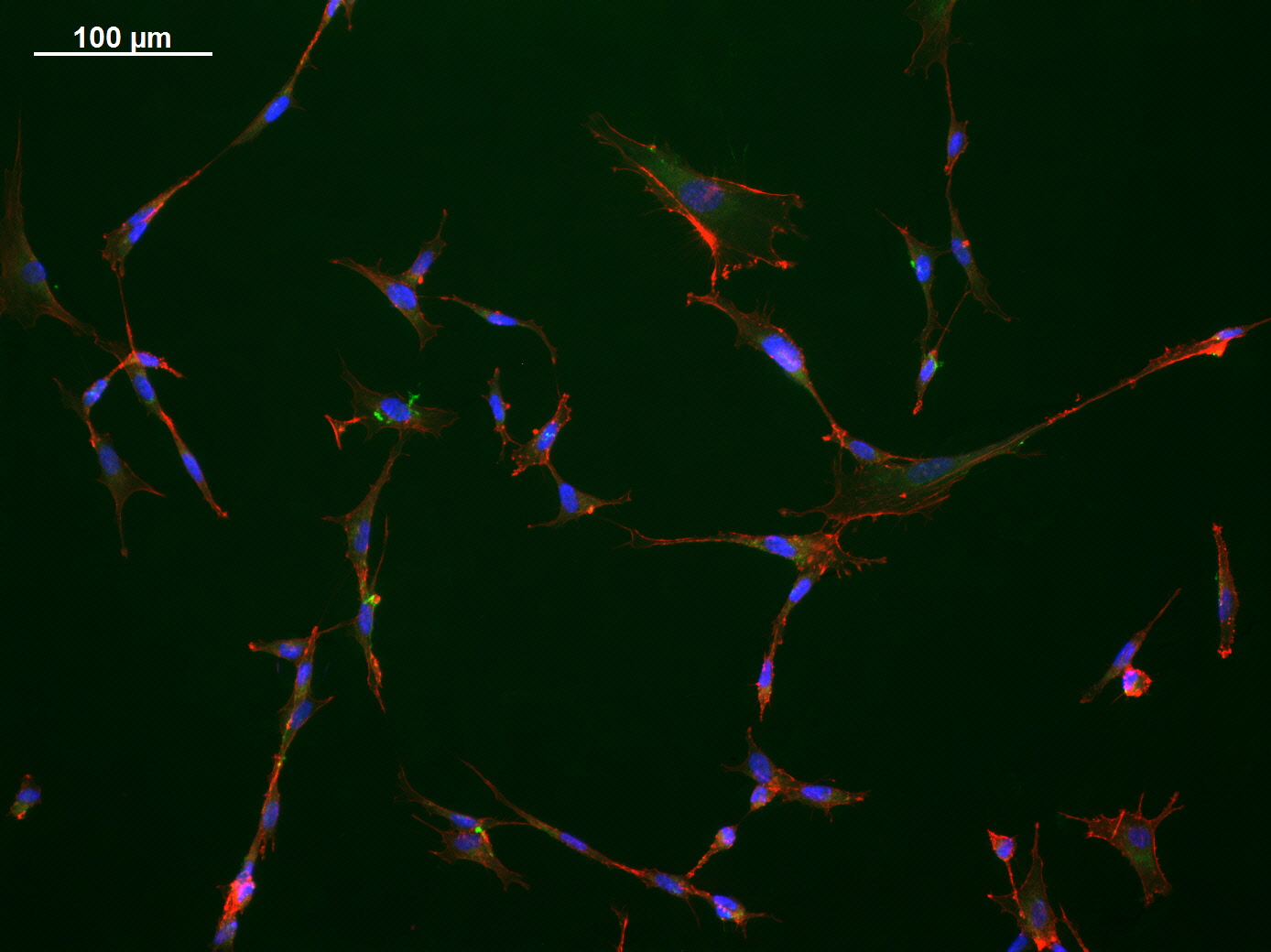

Supplement: S1 File — (ZIP) [file pone.0303106.s001.zip › S1 File/Figure2_TNC_a enviar/AFSCs_TNC_Fig2/7d_m2.JPG]

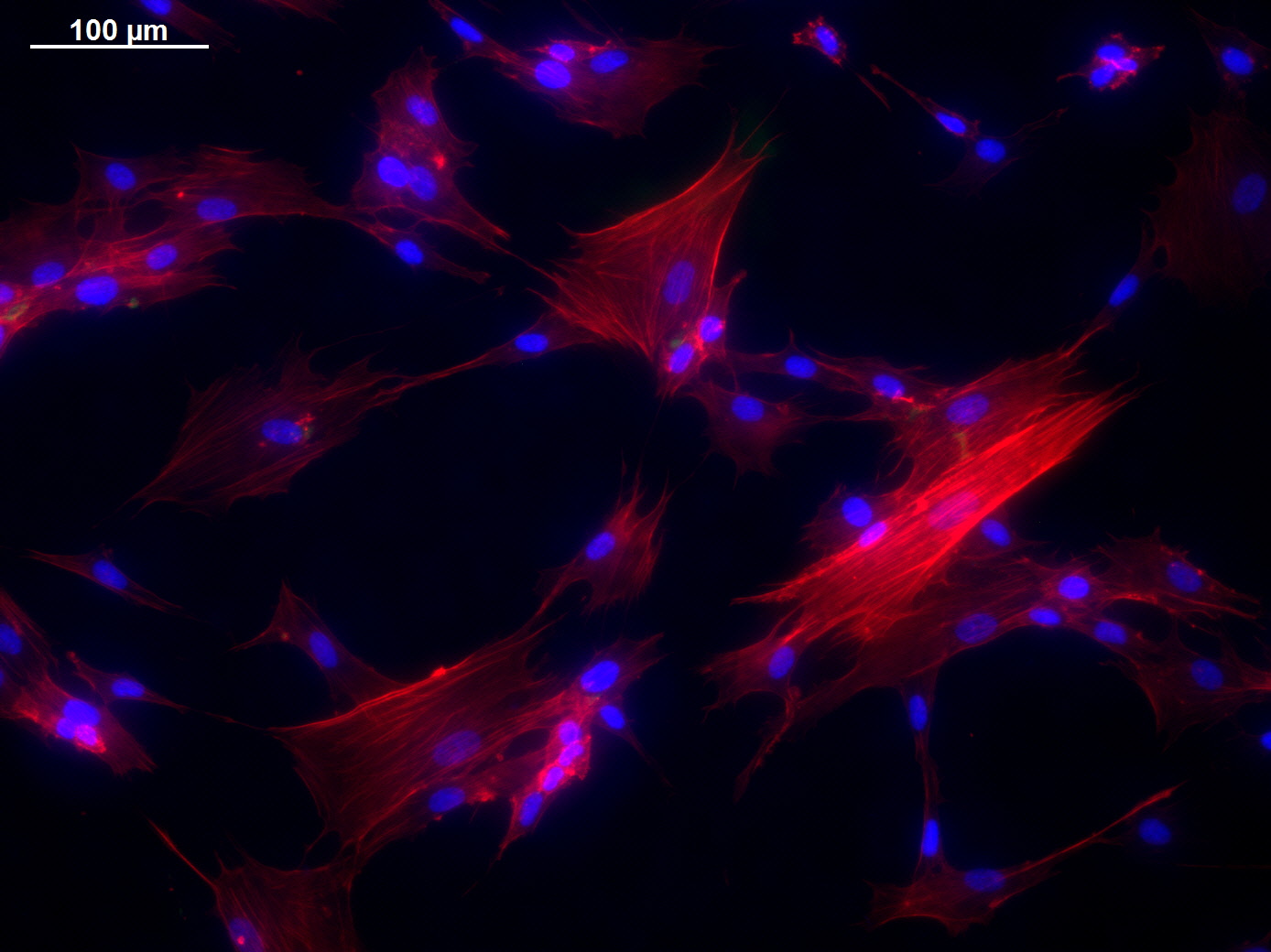

Supplement: S1 File — (ZIP) [file pone.0303106.s001.zip › S1 File/Figure2_TNC_a enviar/AFSCs_TNC_Fig2/7d_m3.JPG]

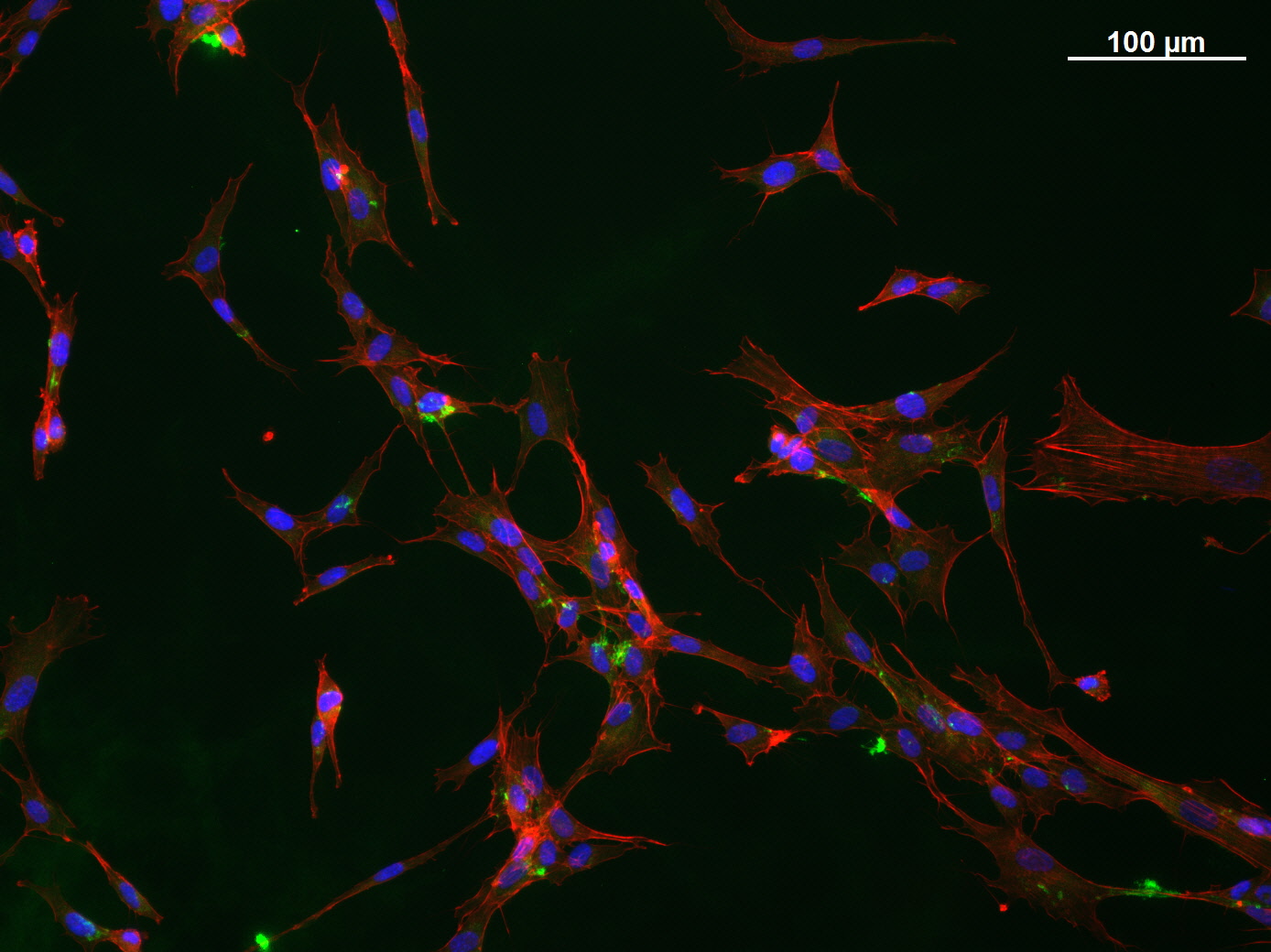

Supplement: S1 File — (ZIP) [file pone.0303106.s001.zip › S1 File/Figure2_TNC_a enviar/AFSCs_TNC_Fig2/7d_m4.JPG]

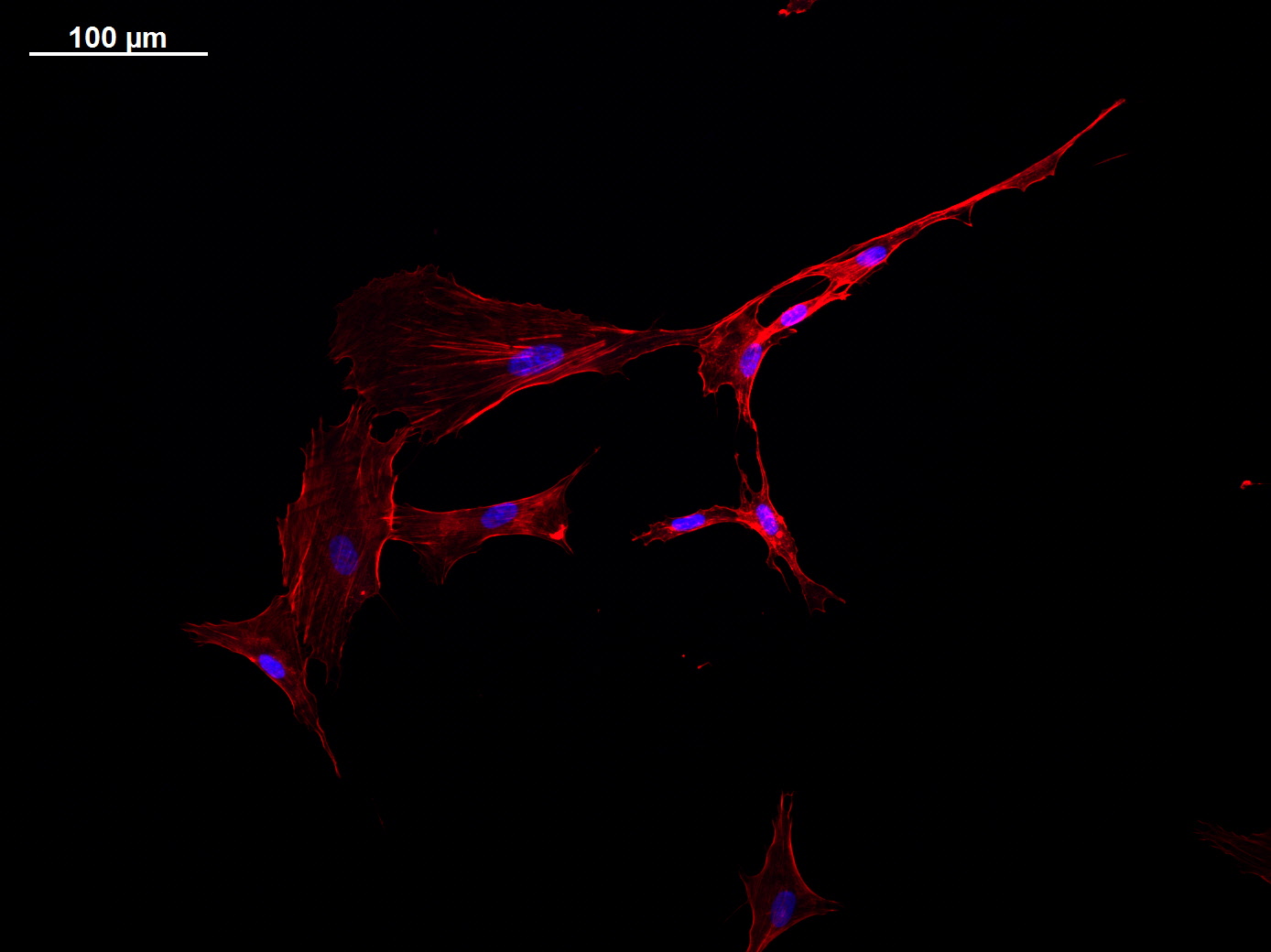

Supplement: S1 File — (ZIP) [file pone.0303106.s001.zip › S1 File/Figure2_TNC_a enviar/AFSCs_TNC_Fig2/7d_m5.JPG]

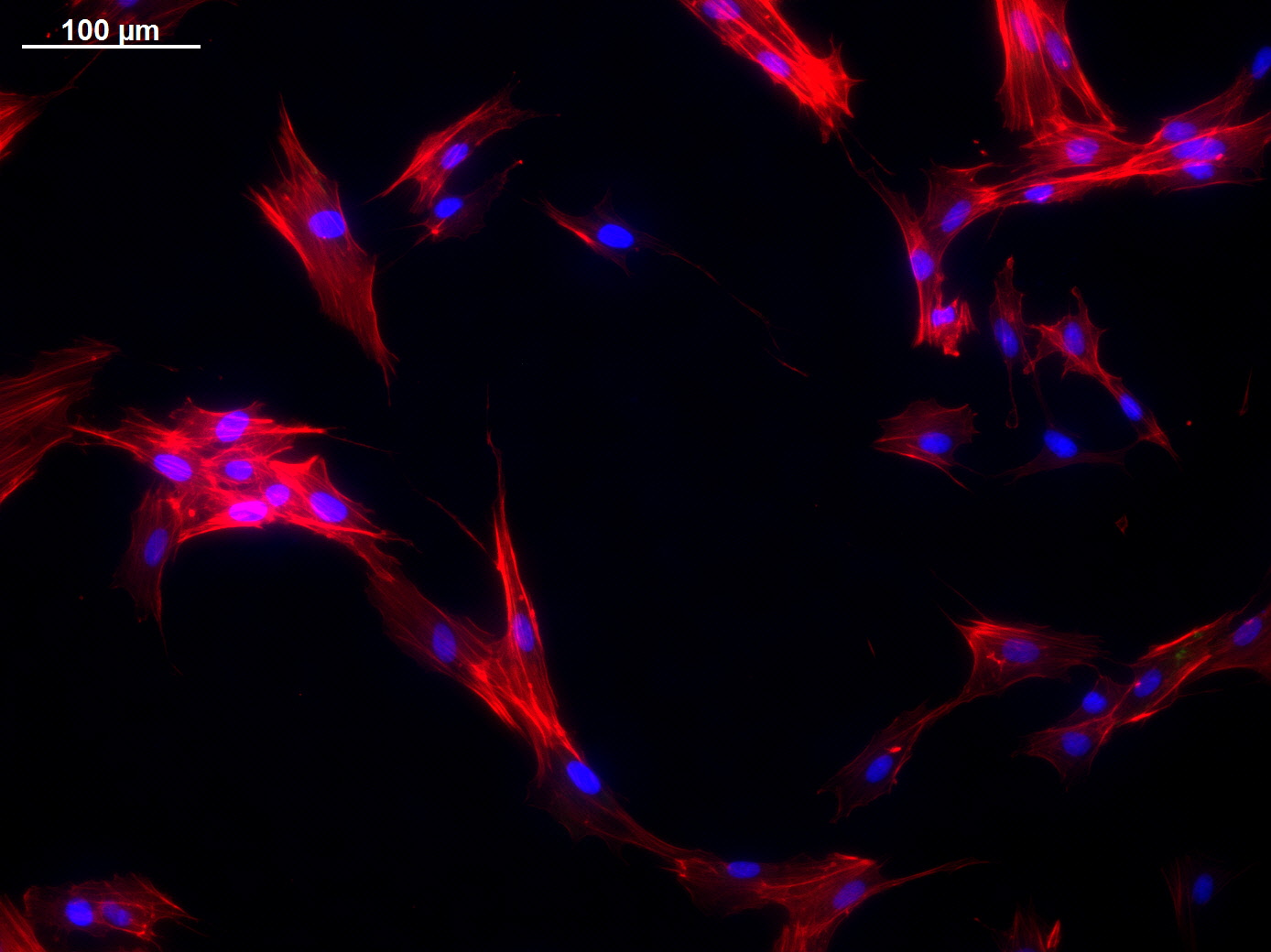

Supplement: S1 File — (ZIP) [file pone.0303106.s001.zip › S1 File/Figure2_TNC_a enviar/AFSCs_TNC_Fig2/7d_m6.JPG]

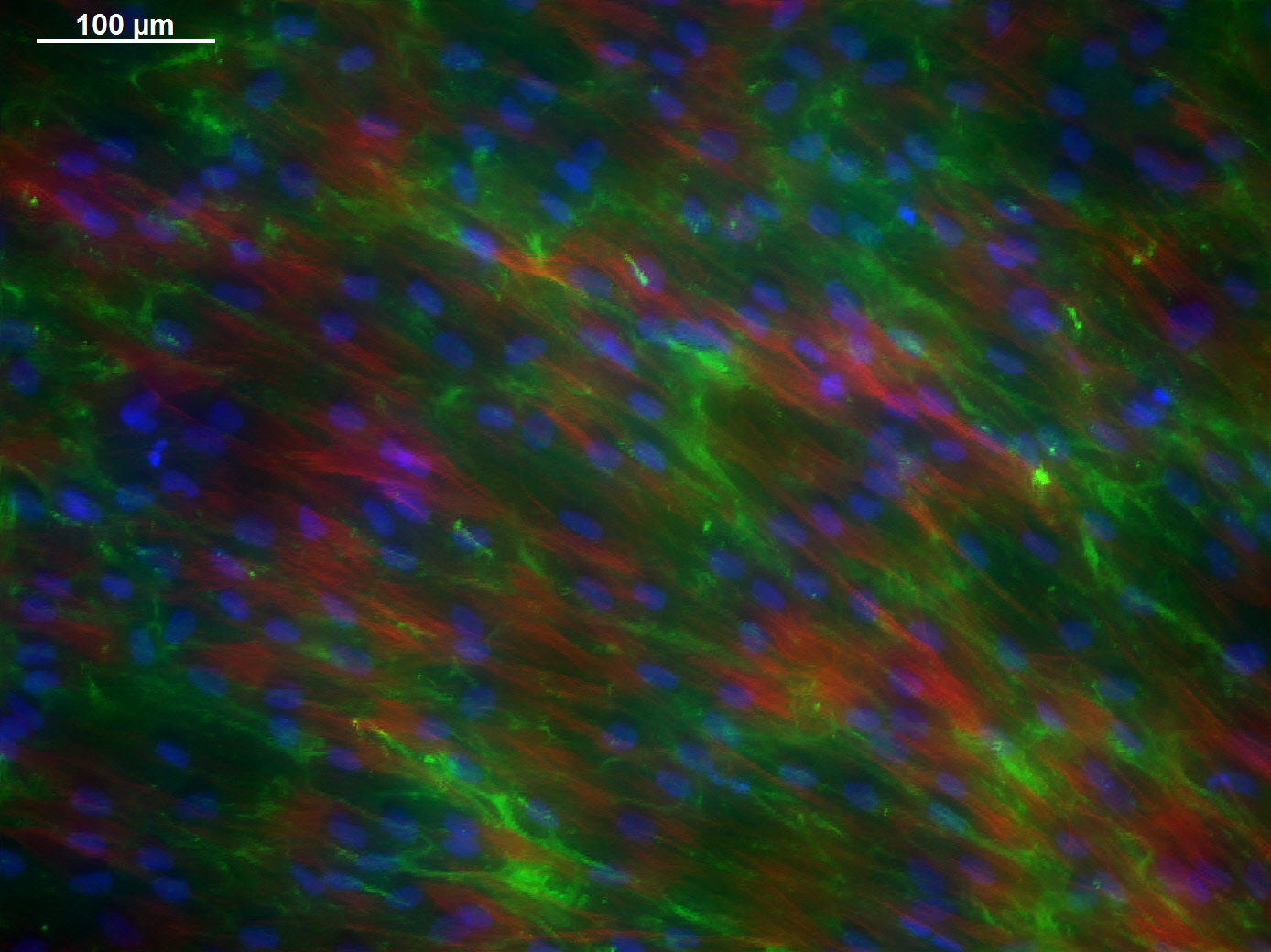

Supplement: S1 File — (ZIP) [file pone.0303106.s001.zip › S1 File/Figure2_TNC_a enviar/ASCs_TNC_Fig2/14d_m1_(c1+c2+c3).JPG]

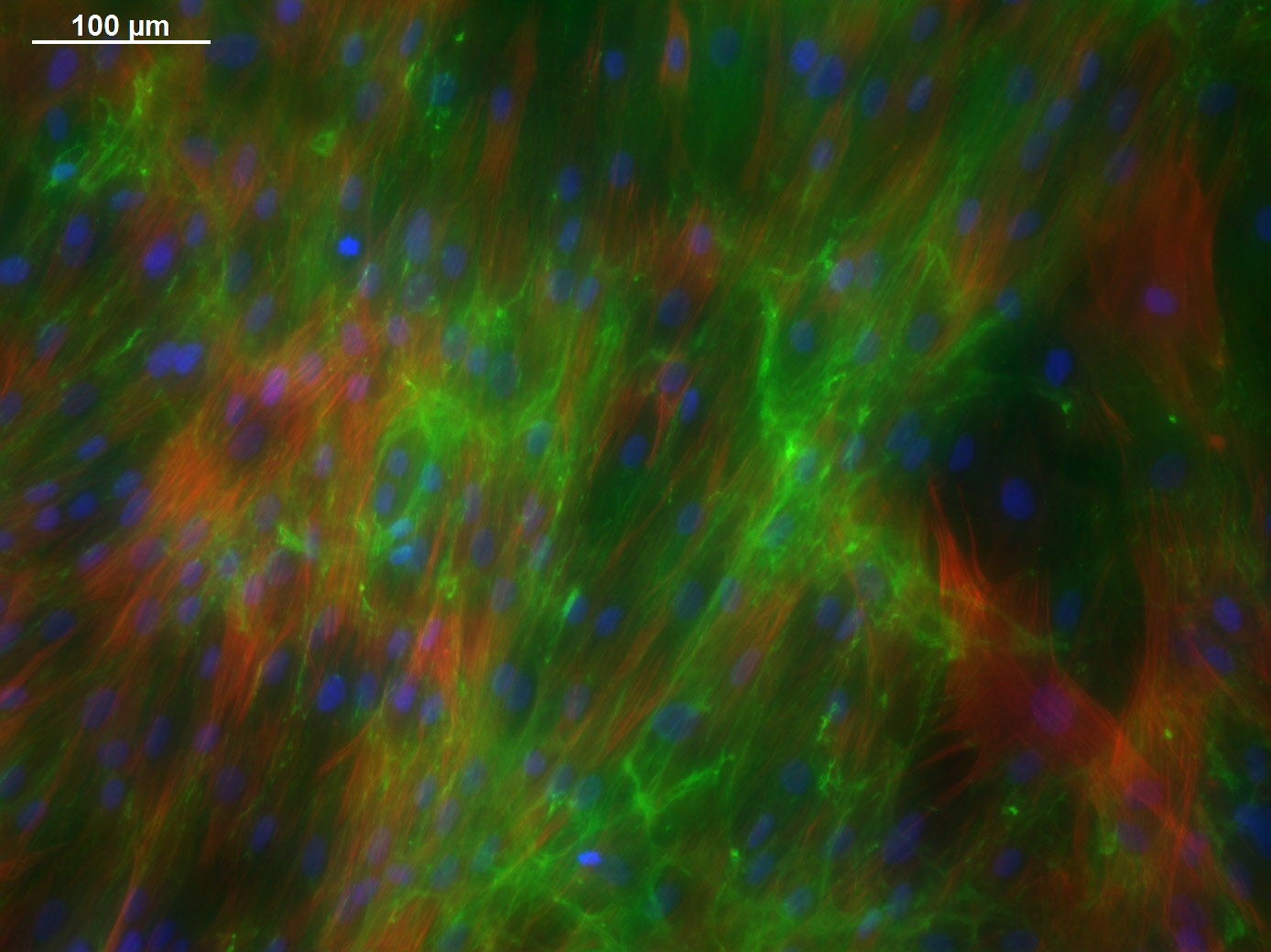

Supplement: S1 File — (ZIP) [file pone.0303106.s001.zip › S1 File/Figure2_TNC_a enviar/ASCs_TNC_Fig2/14d_m2_(c1+c2+c3).JPG]

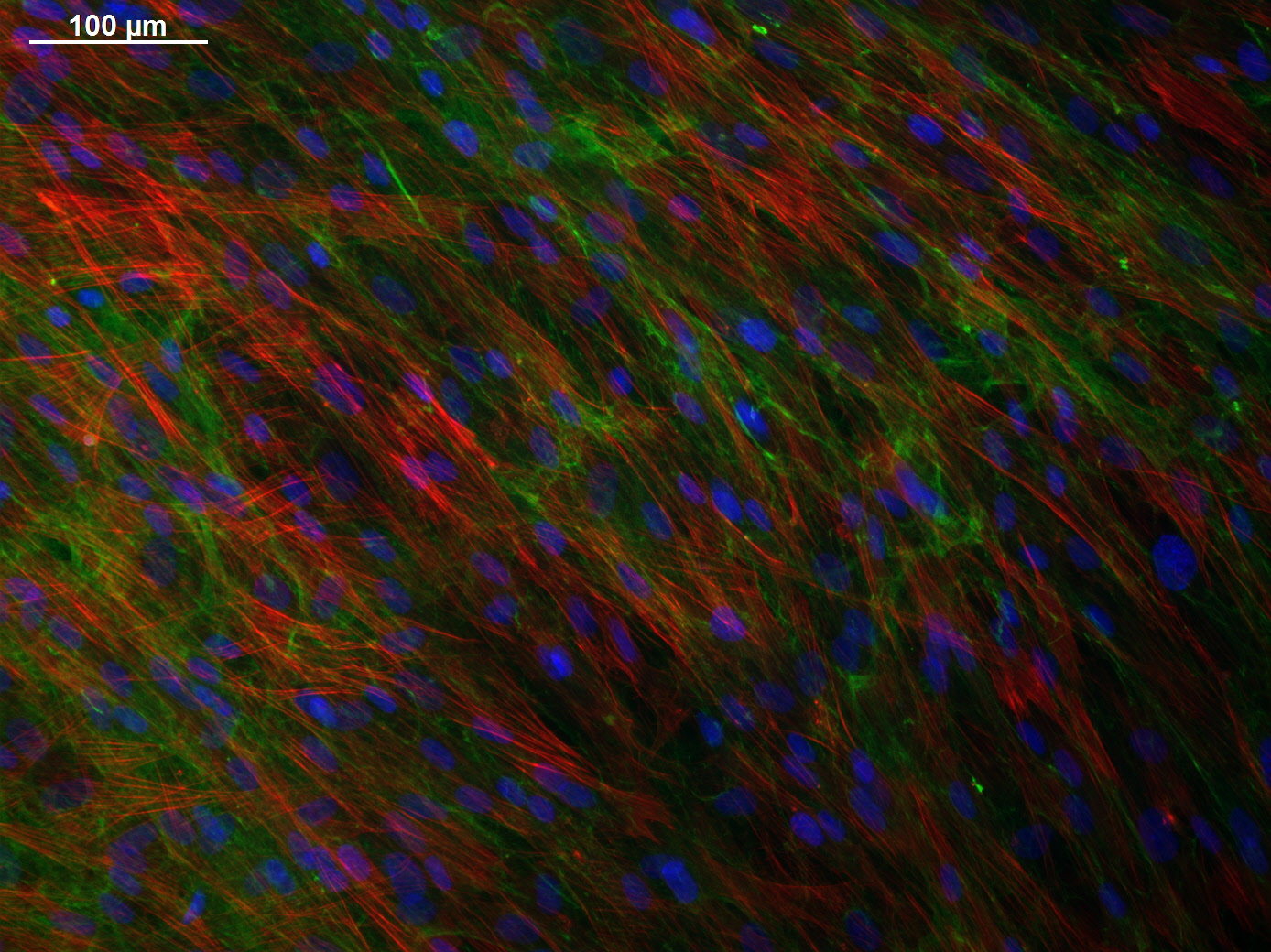

Supplement: S1 File — (ZIP) [file pone.0303106.s001.zip › S1 File/Figure2_TNC_a enviar/ASCs_TNC_Fig2/14d_m3_(c1+c2+c3).JPG]

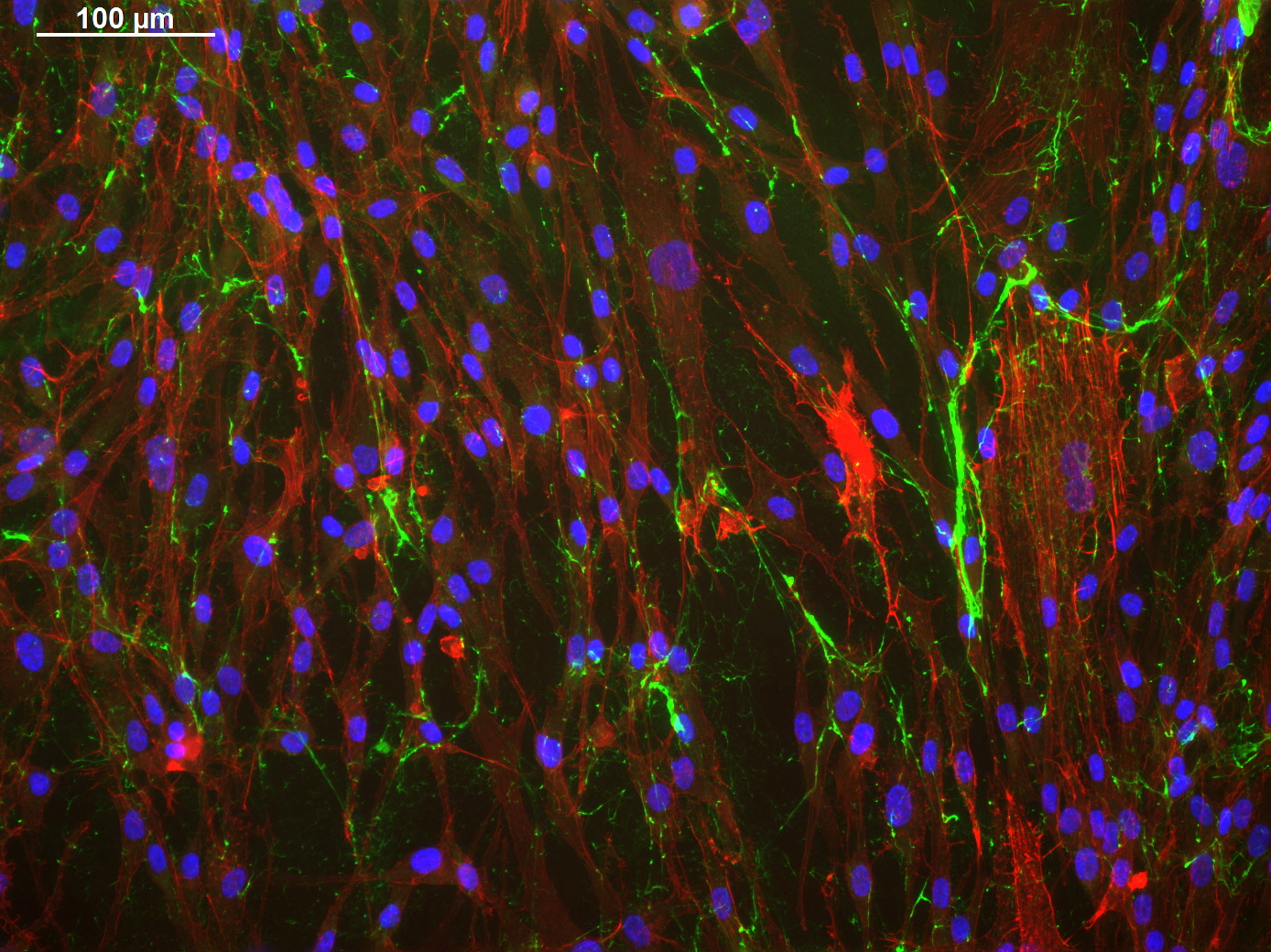

Supplement: S1 File — (ZIP) [file pone.0303106.s001.zip › S1 File/Figure2_TNC_a enviar/ASCs_TNC_Fig2/14d_m4_(c1+c2+c3).JPG]

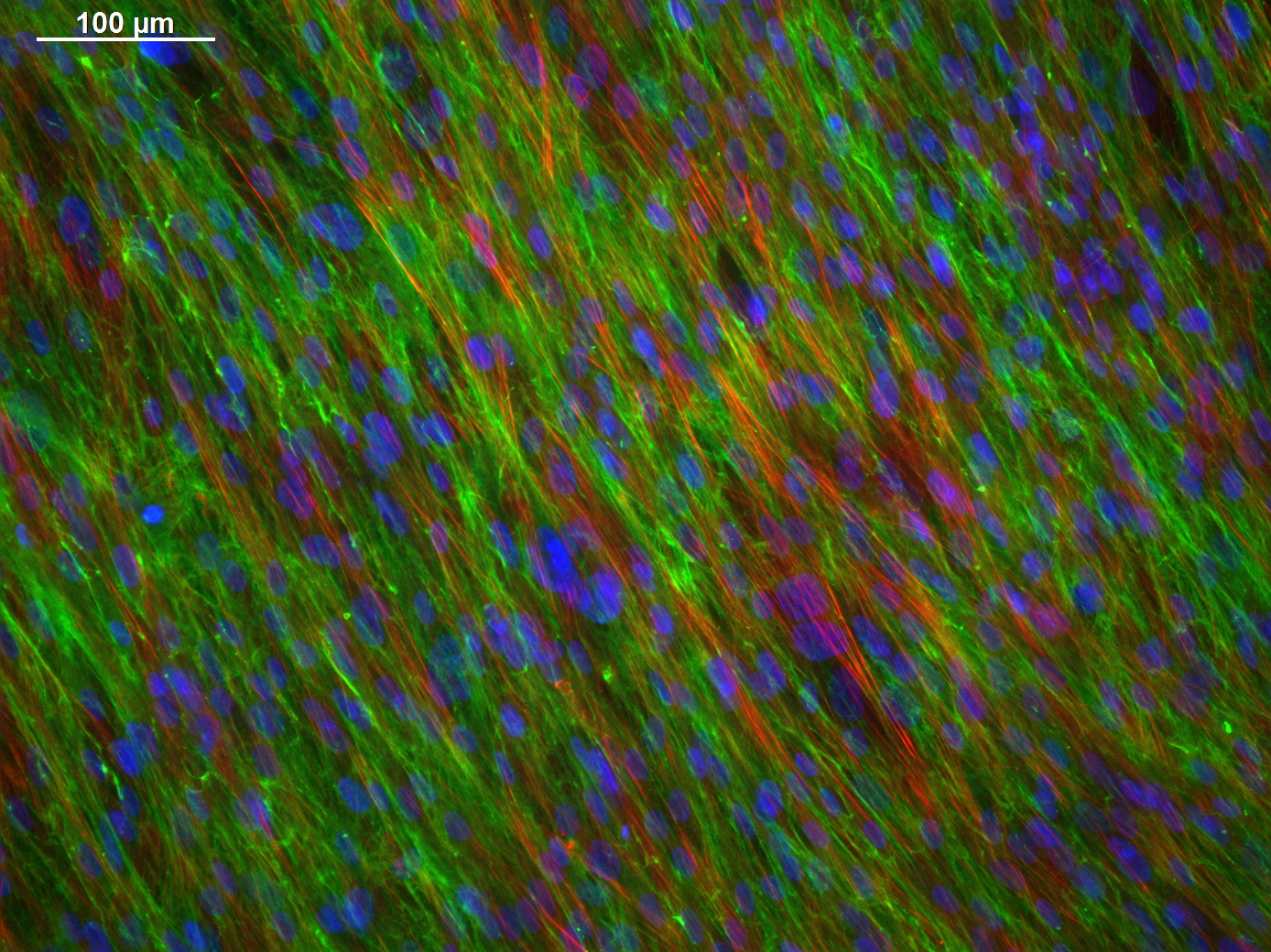

Supplement: S1 File — (ZIP) [file pone.0303106.s001.zip › S1 File/Figure2_TNC_a enviar/ASCs_TNC_Fig2/14d_m5_(c1+c2+c3).JPG]

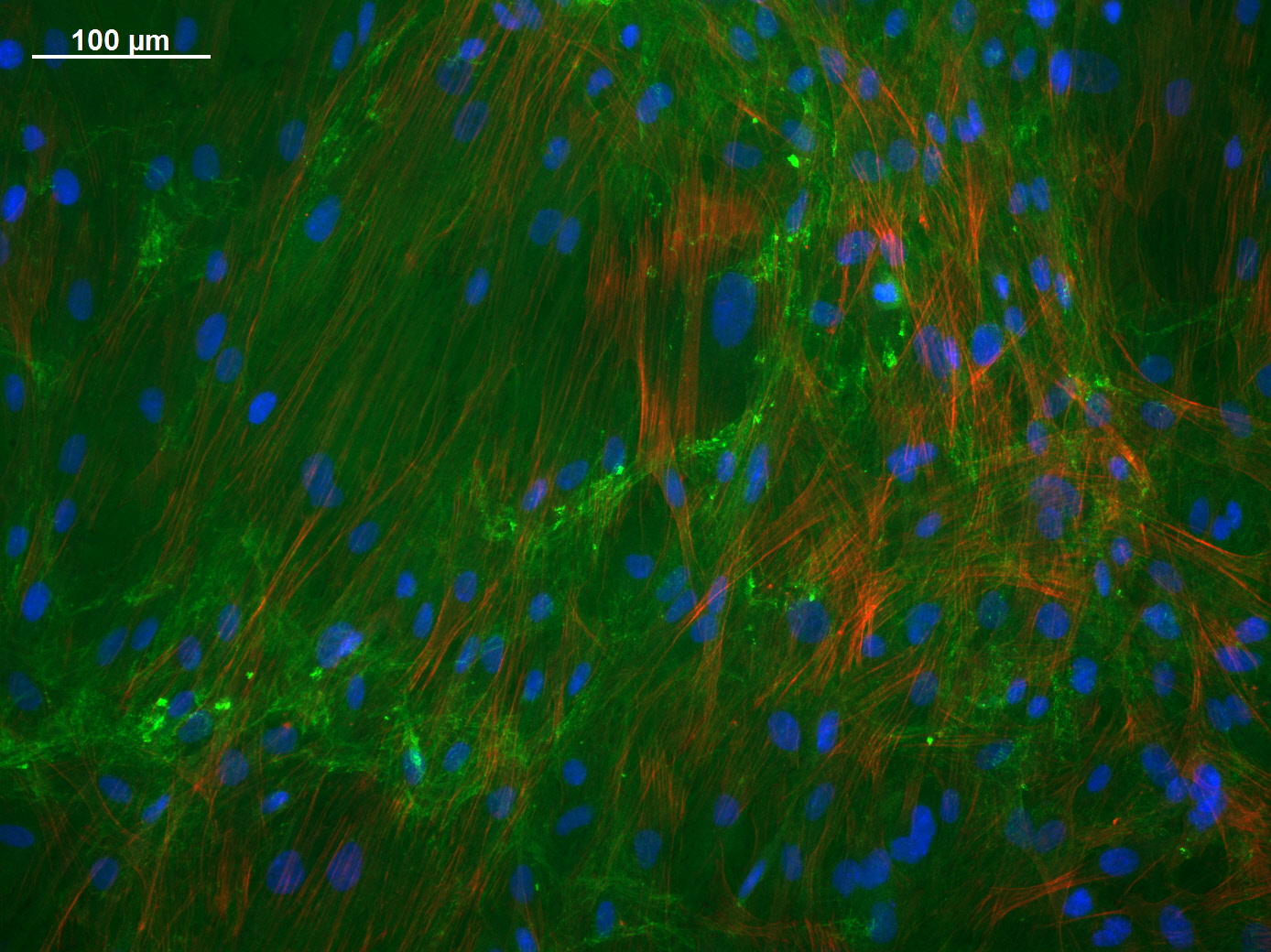

Supplement: S1 File — (ZIP) [file pone.0303106.s001.zip › S1 File/Figure2_TNC_a enviar/ASCs_TNC_Fig2/14d_m6_(c1+c2+c3).JPG]

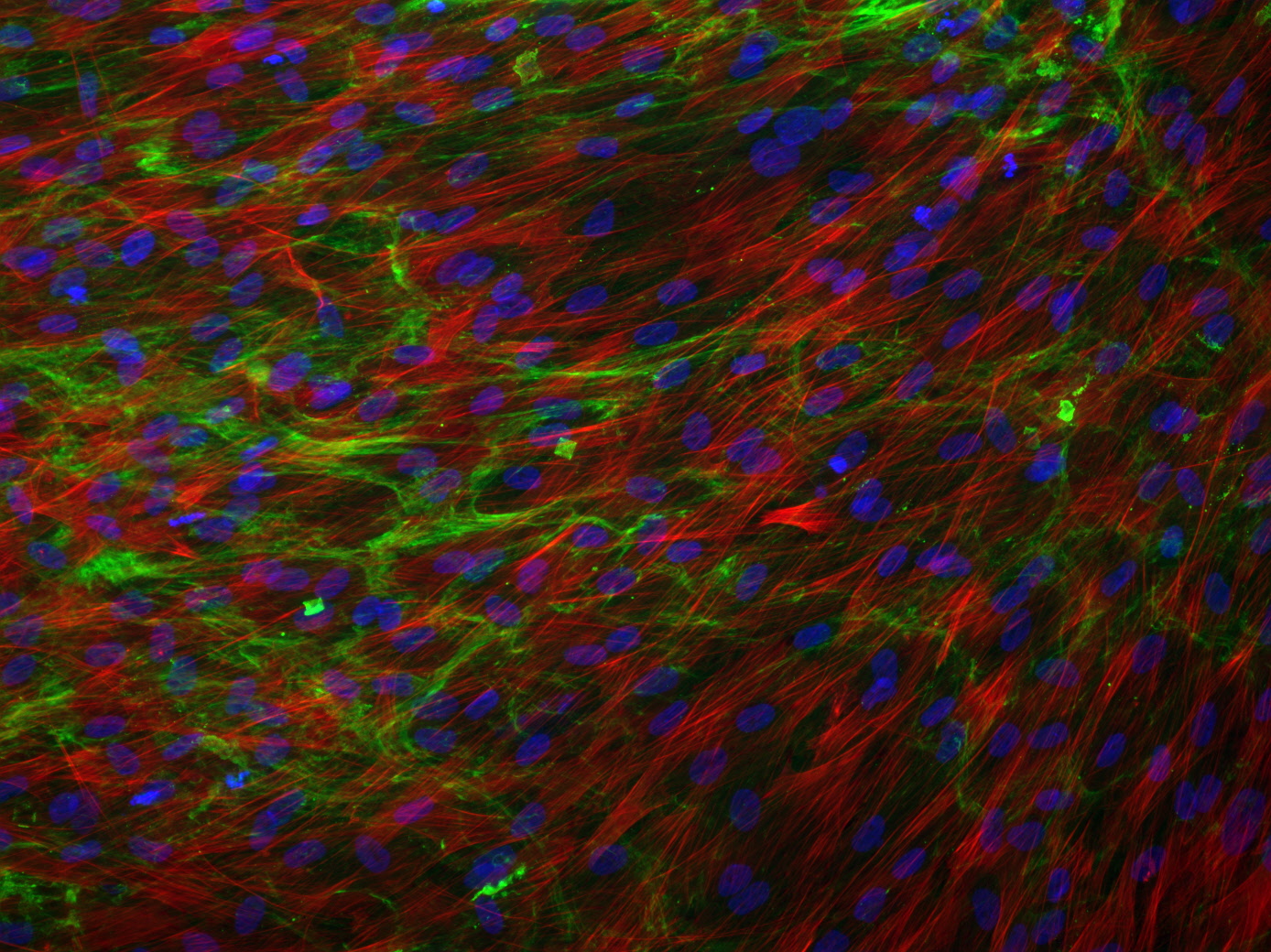

Supplement: S1 File — (ZIP) [file pone.0303106.s001.zip › S1 File/Figure2_TNC_a enviar/ASCs_TNC_Fig2/21d m1_(c1+c2+c3).JPG]

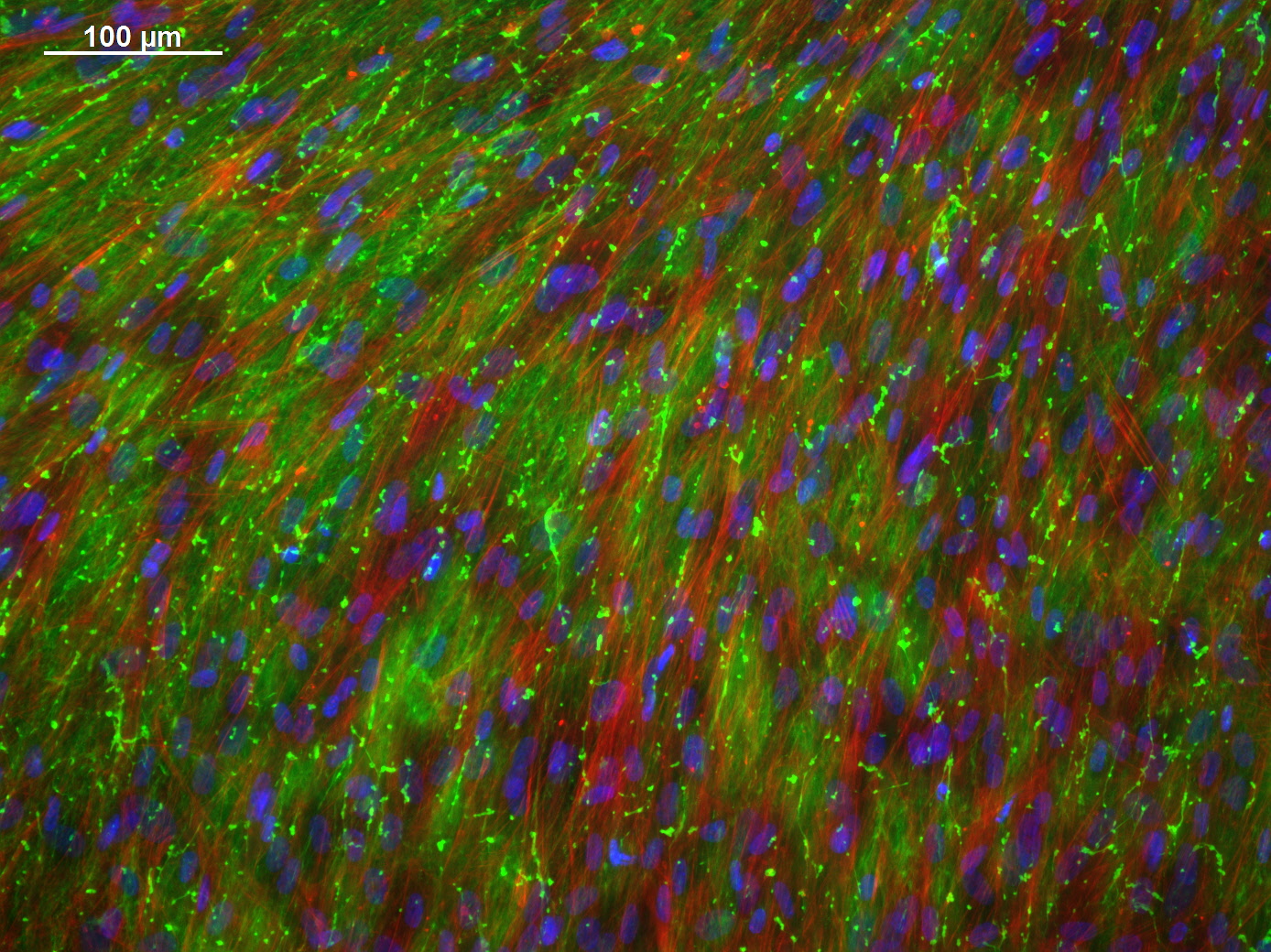

Supplement: S1 File — (ZIP) [file pone.0303106.s001.zip › S1 File/Figure2_TNC_a enviar/ASCs_TNC_Fig2/21d_m2_(c1+c2+c3).JPG]

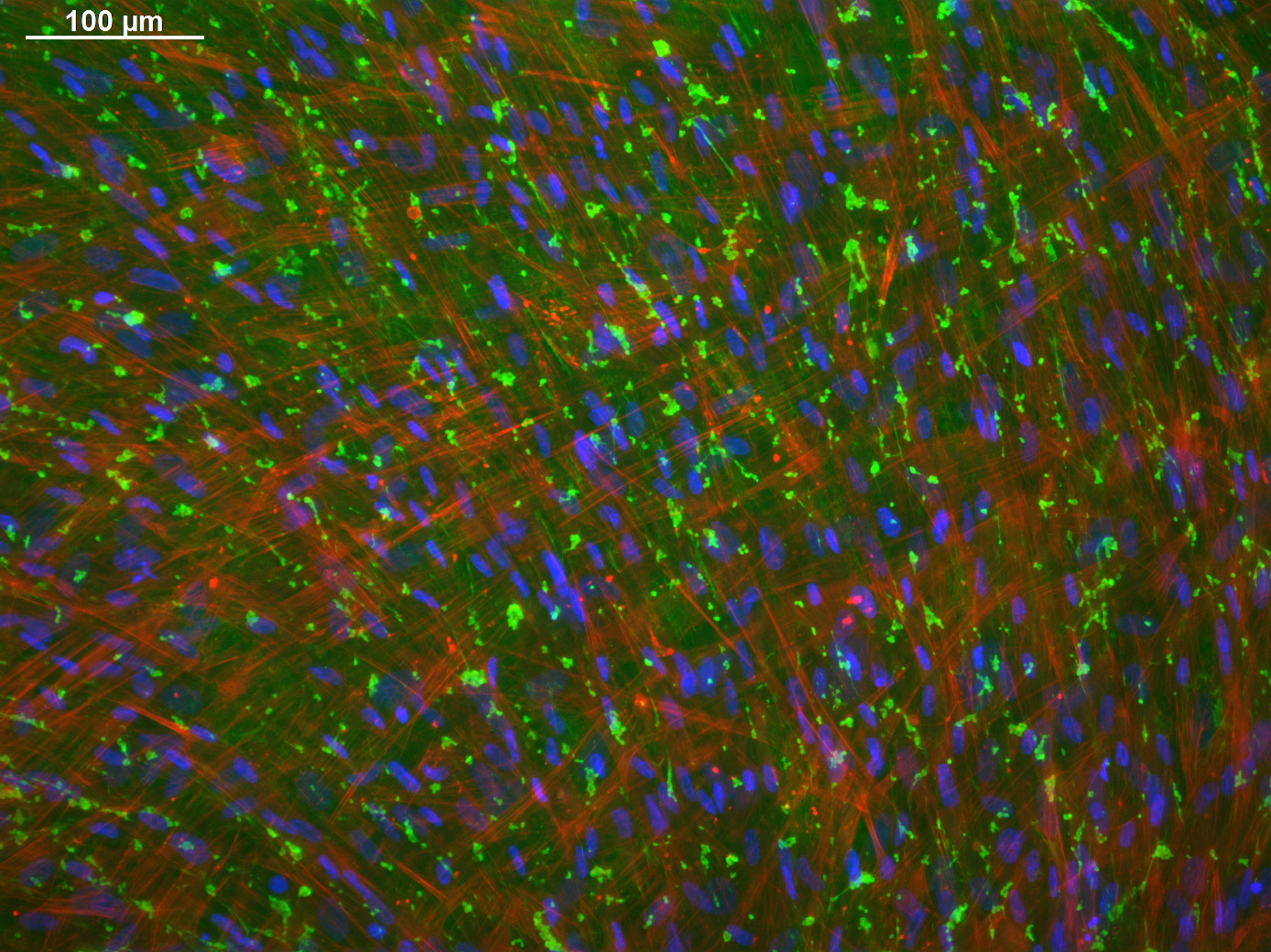

Supplement: S1 File — (ZIP) [file pone.0303106.s001.zip › S1 File/Figure2_TNC_a enviar/ASCs_TNC_Fig2/21d_m3_(c1+c2+c3).JPG]

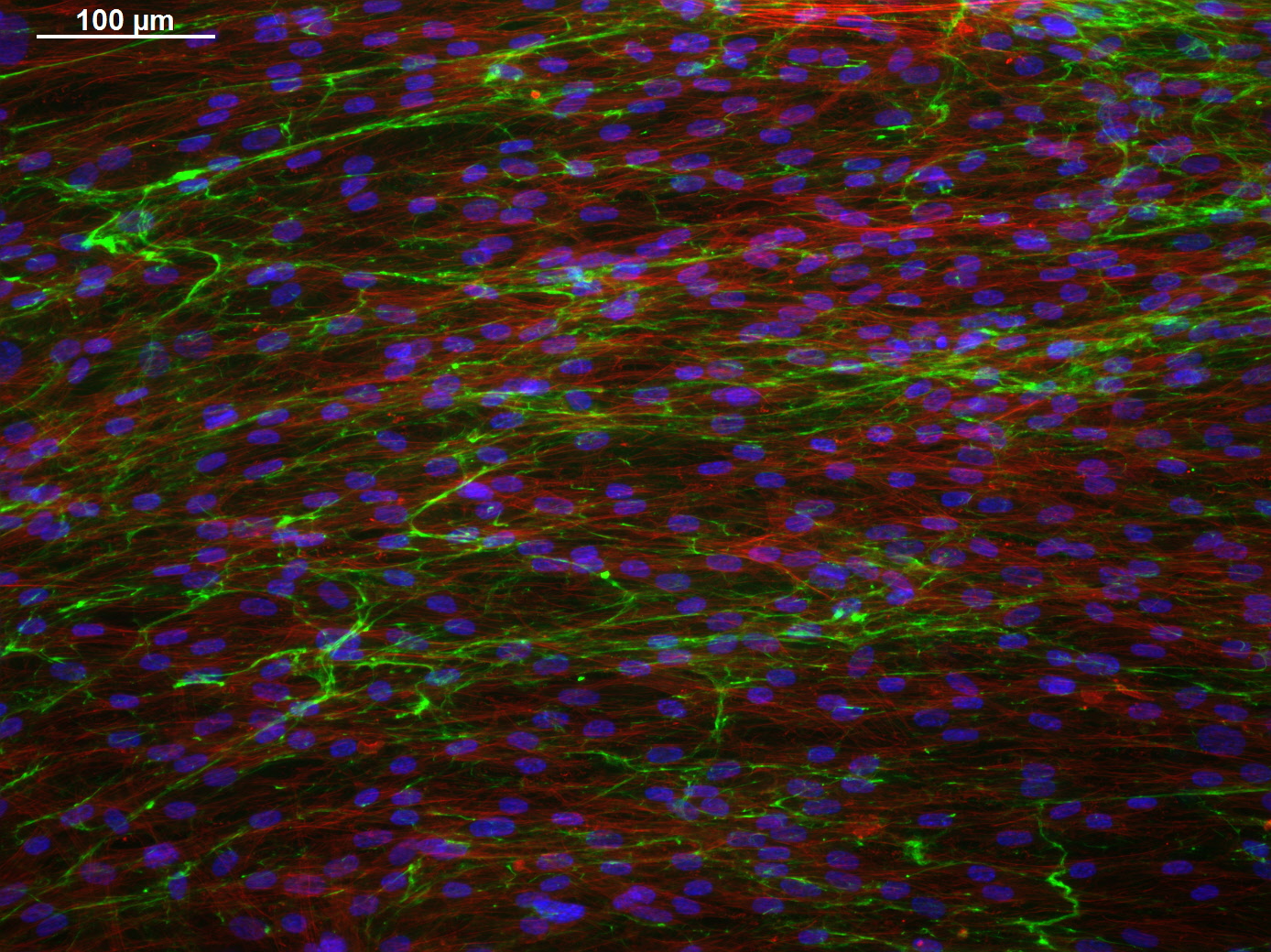

Supplement: S1 File — (ZIP) [file pone.0303106.s001.zip › S1 File/Figure2_TNC_a enviar/ASCs_TNC_Fig2/21d_m4_(c1+c2+c3).JPG]

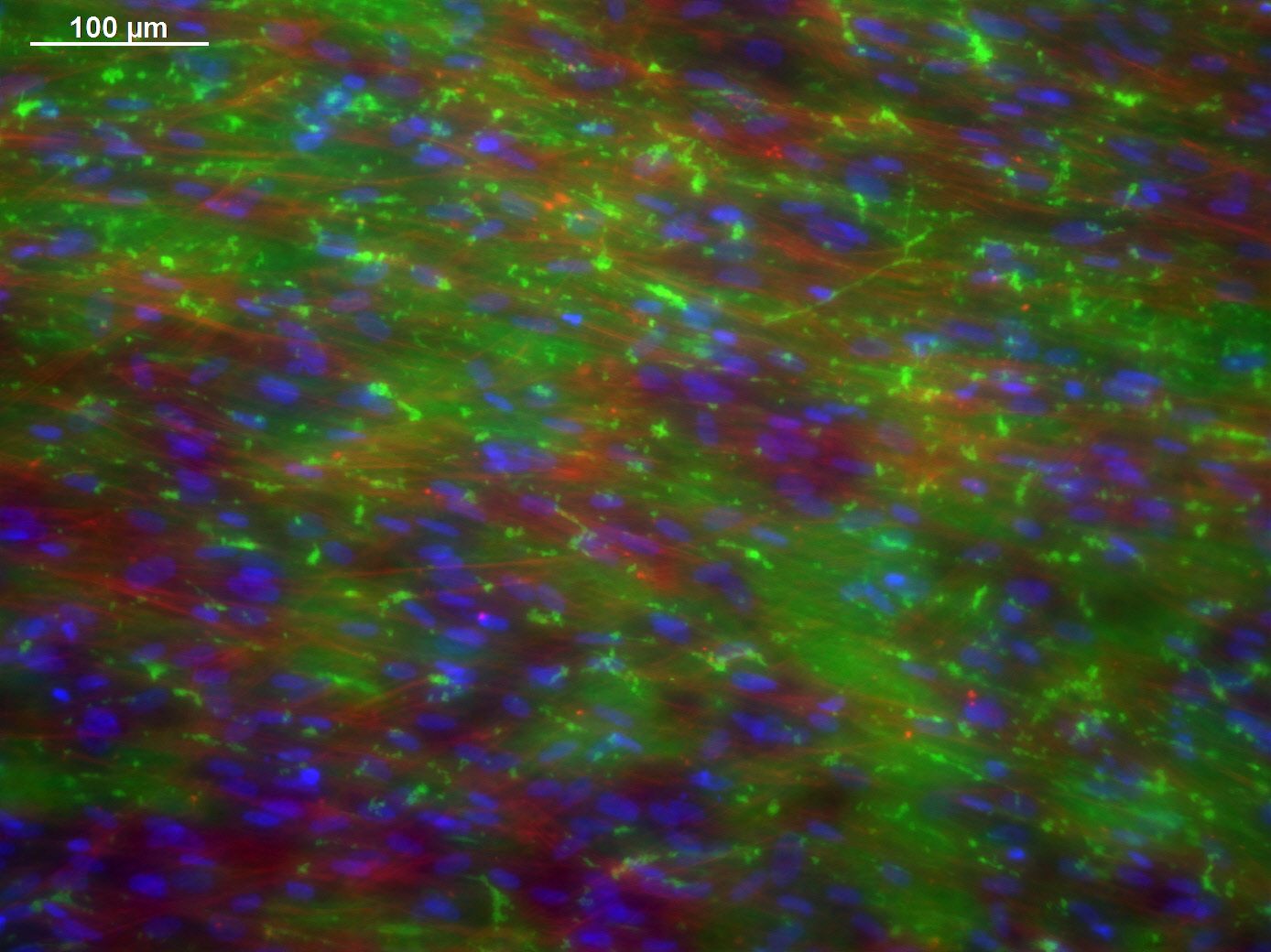

Supplement: S1 File — (ZIP) [file pone.0303106.s001.zip › S1 File/Figure2_TNC_a enviar/ASCs_TNC_Fig2/21d_m5_(c1+c2+c3).JPG]

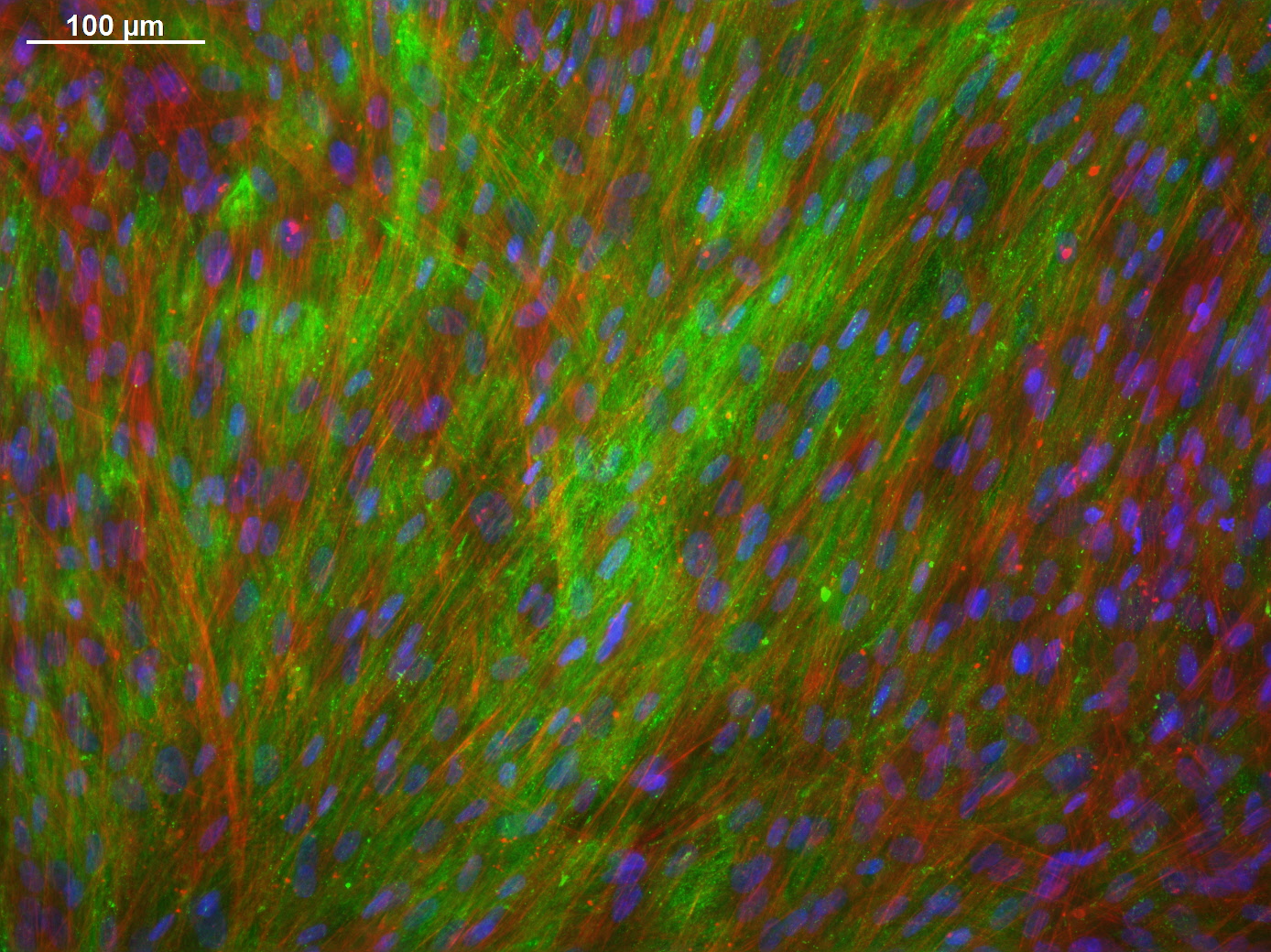

Supplement: S1 File — (ZIP) [file pone.0303106.s001.zip › S1 File/Figure2_TNC_a enviar/ASCs_TNC_Fig2/21d_m6_(c1+c2+c3).JPG]

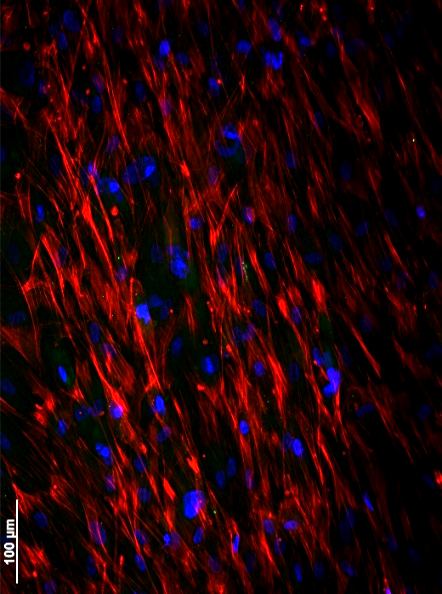

Supplement: S1 File — (ZIP) [file pone.0303106.s001.zip › S1 File/Figure2_TNC_a enviar/ASCs_TNC_Fig2/28d_m1_(c1+c2+c3).png]

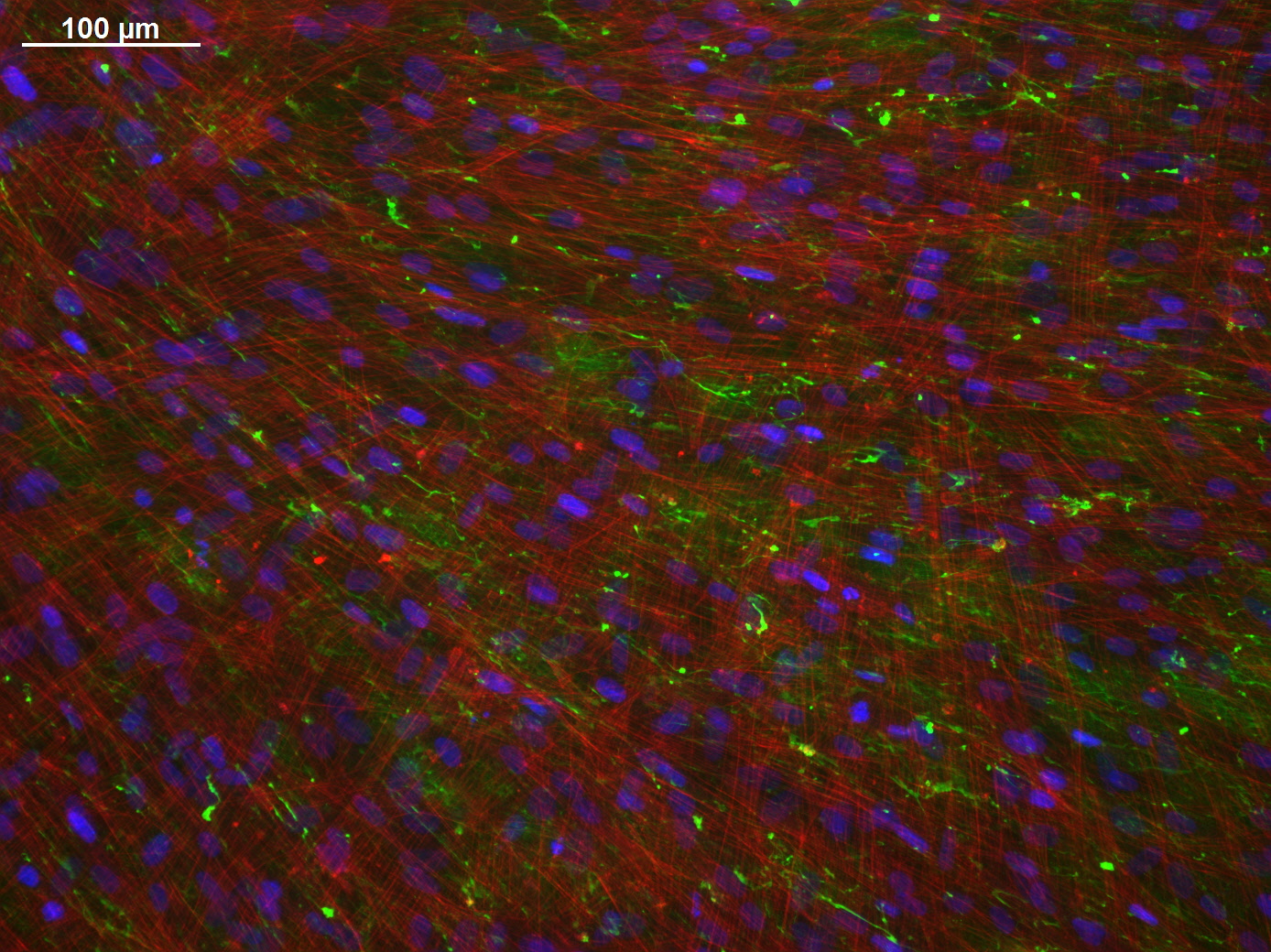

Supplement: S1 File — (ZIP) [file pone.0303106.s001.zip › S1 File/Figure2_TNC_a enviar/ASCs_TNC_Fig2/28d_m2_(c1+c2+c3).JPG]

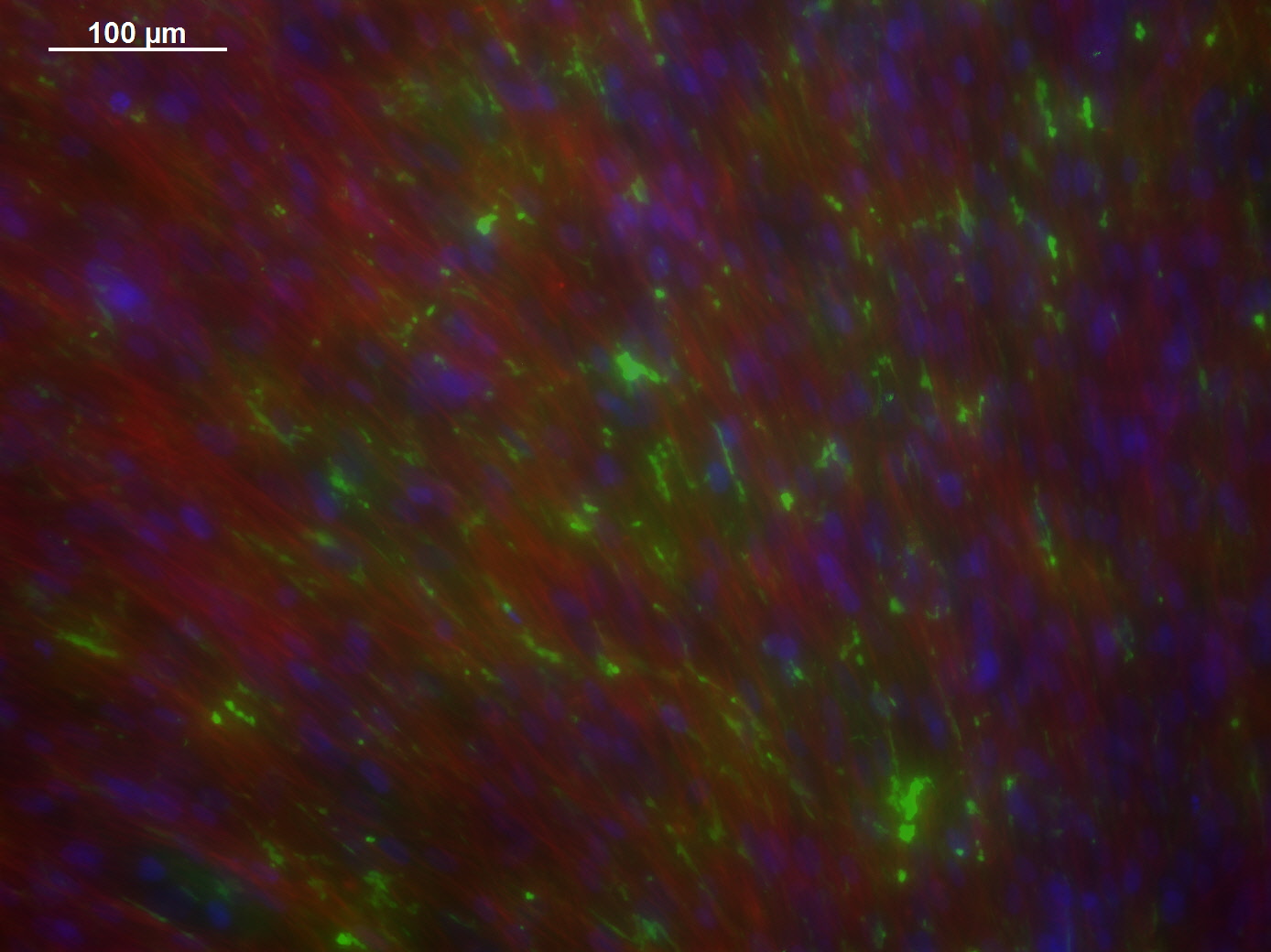

Supplement: S1 File — (ZIP) [file pone.0303106.s001.zip › S1 File/Figure2_TNC_a enviar/ASCs_TNC_Fig2/28d_m3_(c1+c2+c3).JPG]

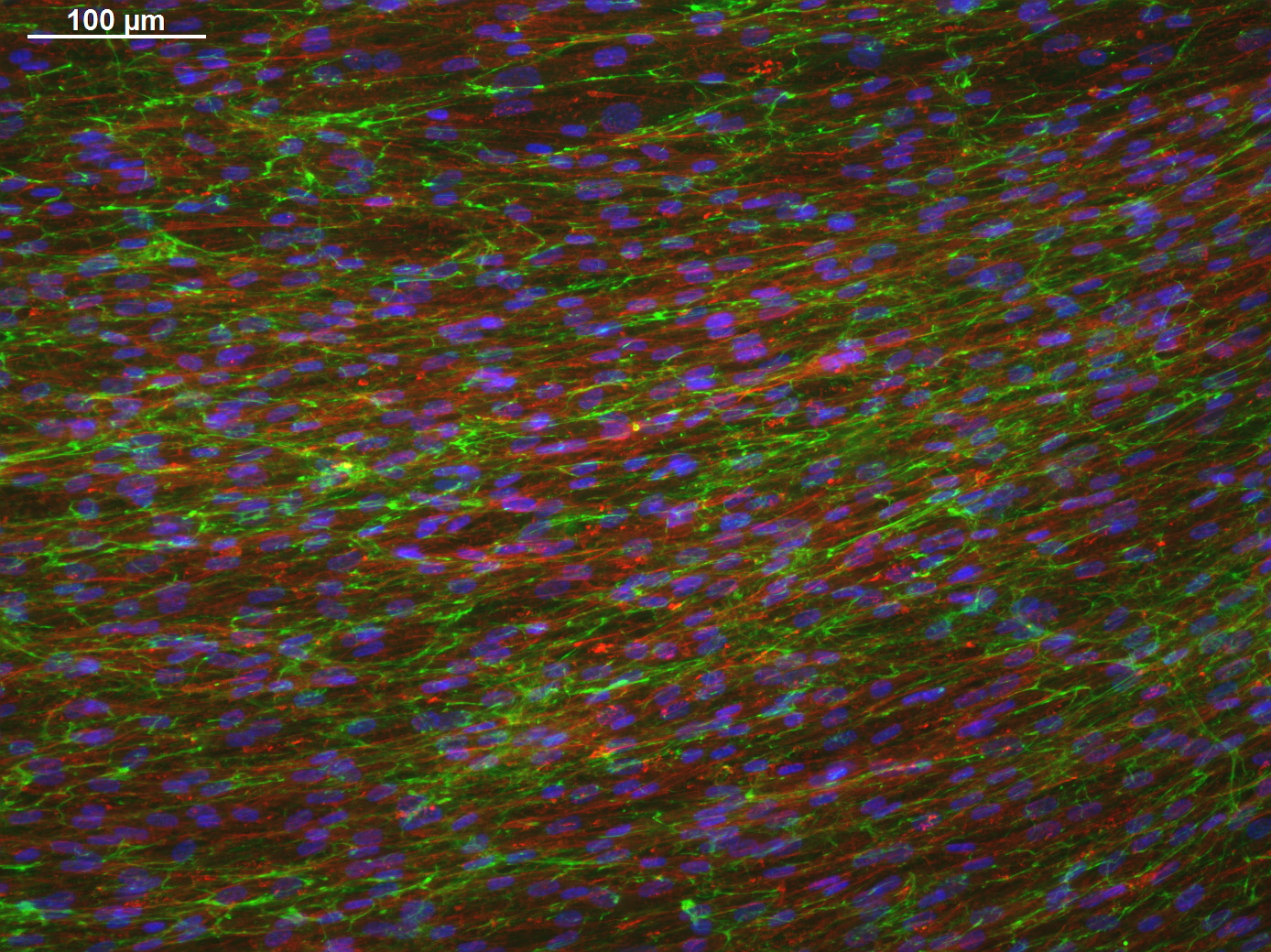

Supplement: S1 File — (ZIP) [file pone.0303106.s001.zip › S1 File/Figure2_TNC_a enviar/ASCs_TNC_Fig2/28d_m4_(c1+c2+c3).JPG]

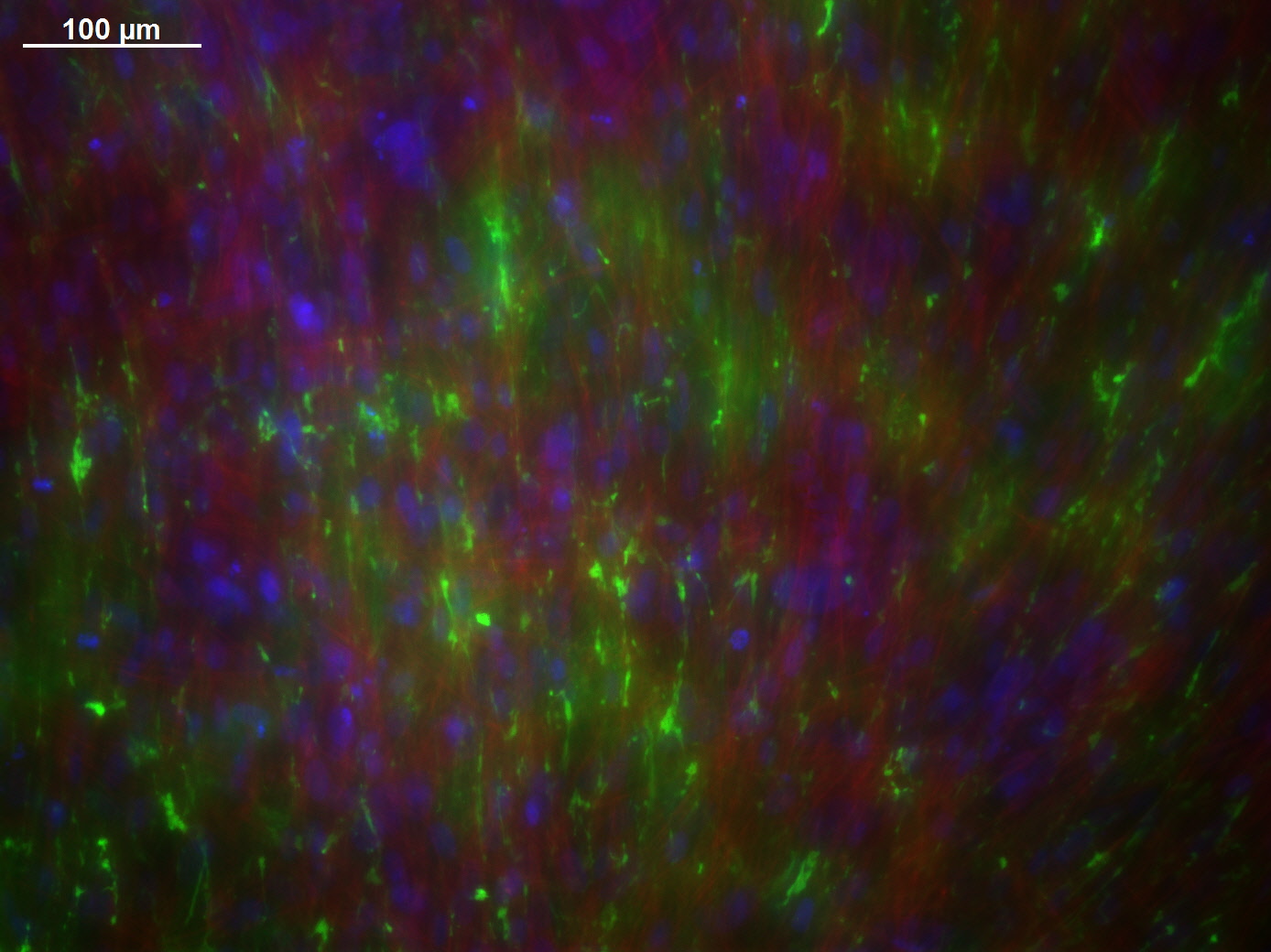

Supplement: S1 File — (ZIP) [file pone.0303106.s001.zip › S1 File/Figure2_TNC_a enviar/ASCs_TNC_Fig2/28d_m5_(c1+c2+c3).JPG]

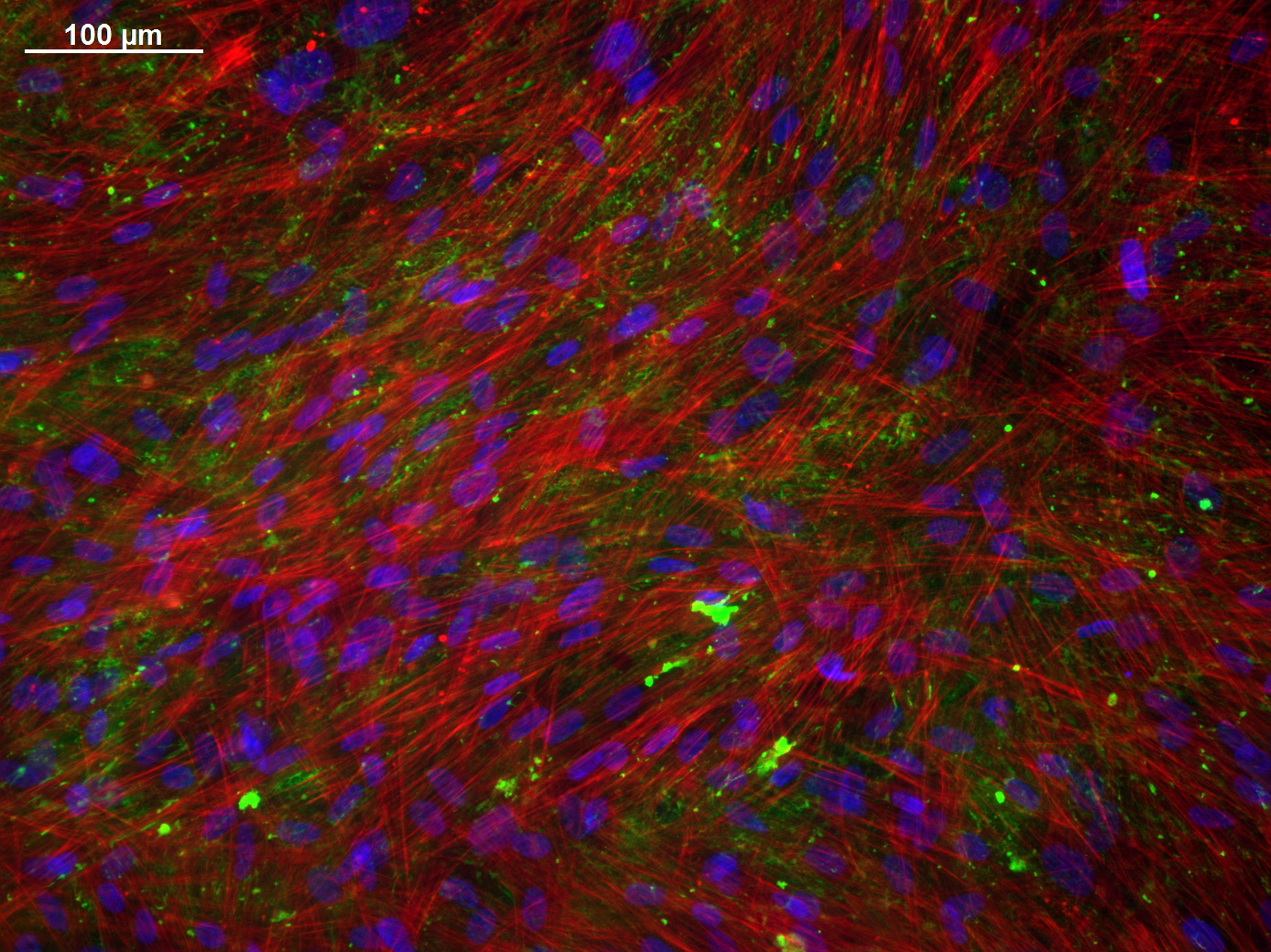

Supplement: S1 File — (ZIP) [file pone.0303106.s001.zip › S1 File/Figure2_TNC_a enviar/ASCs_TNC_Fig2/28d_m6_(c1+c2+c3).JPG]

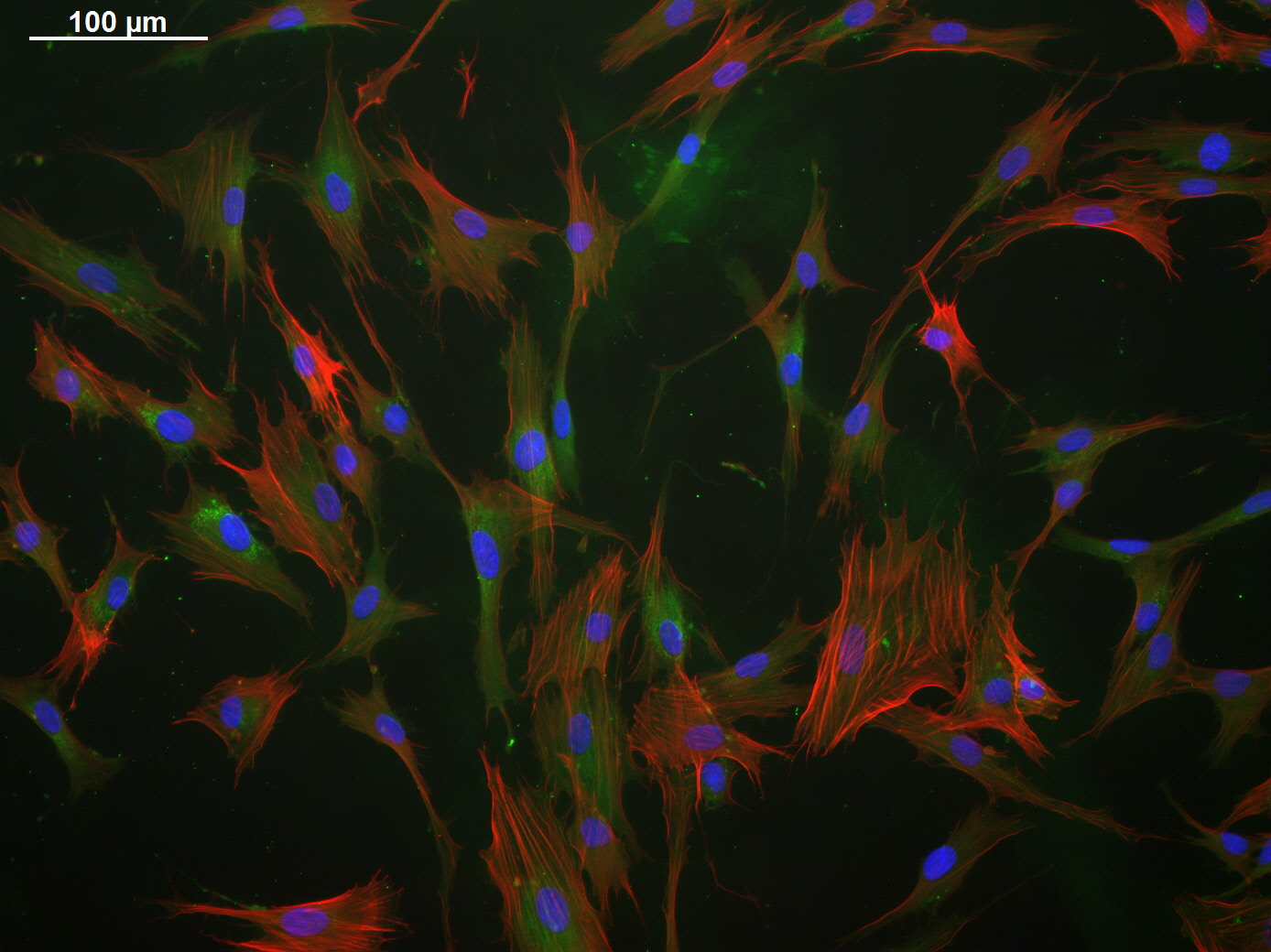

Supplement: S1 File — (ZIP) [file pone.0303106.s001.zip › S1 File/Figure2_TNC_a enviar/ASCs_TNC_Fig2/7d_m1_(c1+c2+c3).JPG]

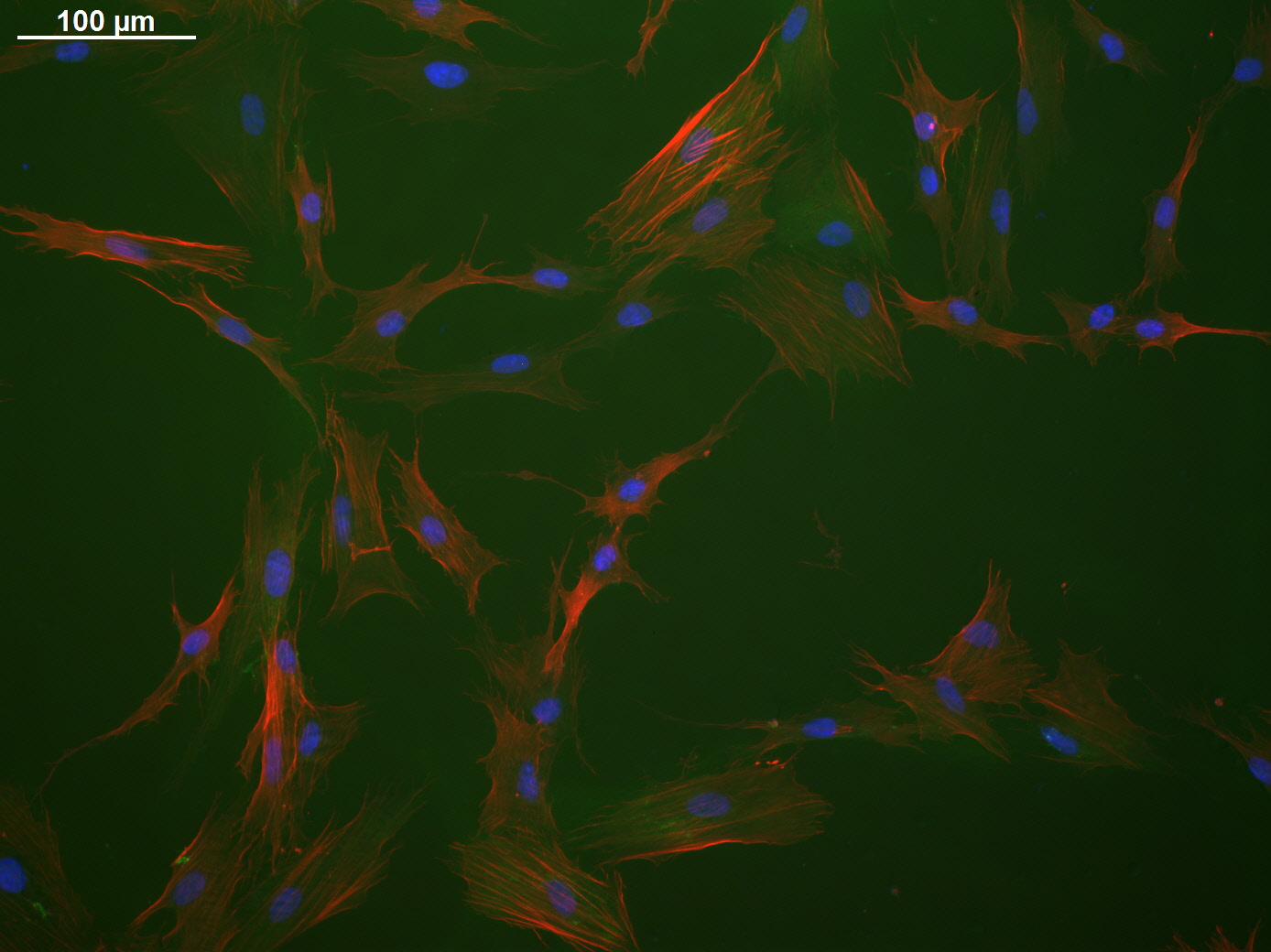

Supplement: S1 File — (ZIP) [file pone.0303106.s001.zip › S1 File/Figure2_TNC_a enviar/ASCs_TNC_Fig2/7d_m2_(c1+c2+c3).JPG]

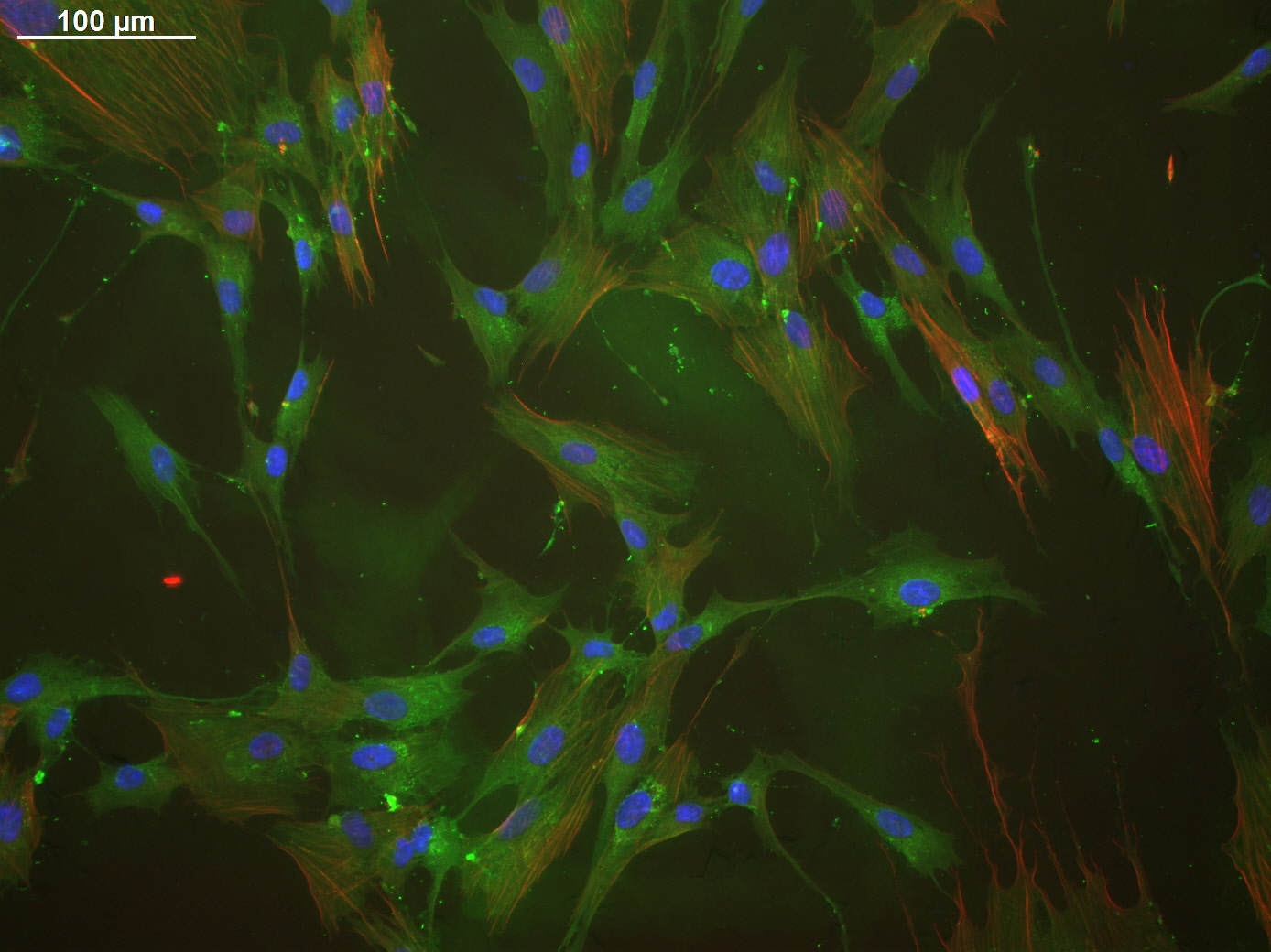

Supplement: S1 File — (ZIP) [file pone.0303106.s001.zip › S1 File/Figure2_TNC_a enviar/ASCs_TNC_Fig2/7d_m3_(c1+c2+c3).JPG]

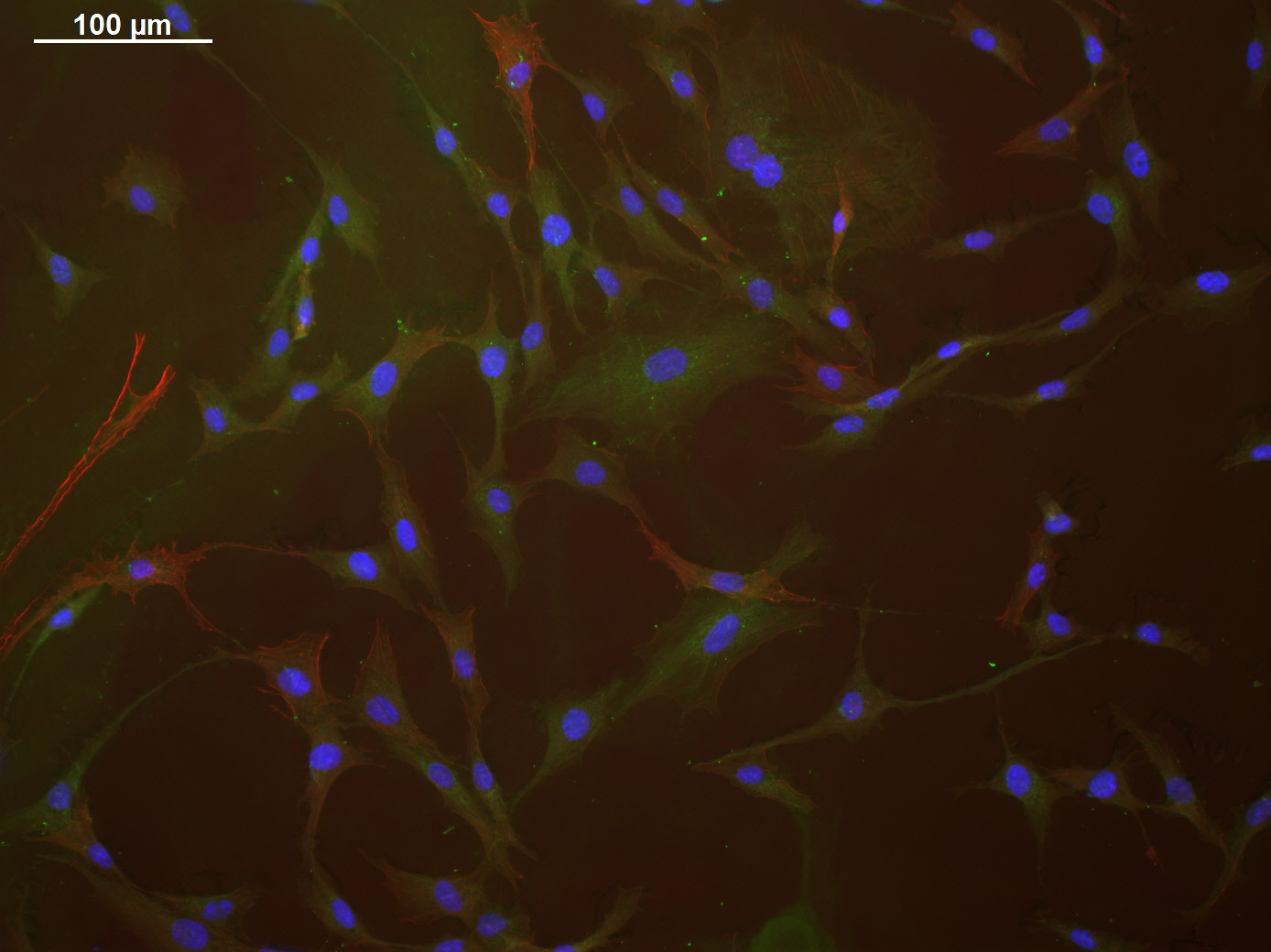

Supplement: S1 File — (ZIP) [file pone.0303106.s001.zip › S1 File/Figure2_TNC_a enviar/ASCs_TNC_Fig2/7d_m4_(c1+c2+c3).JPG]

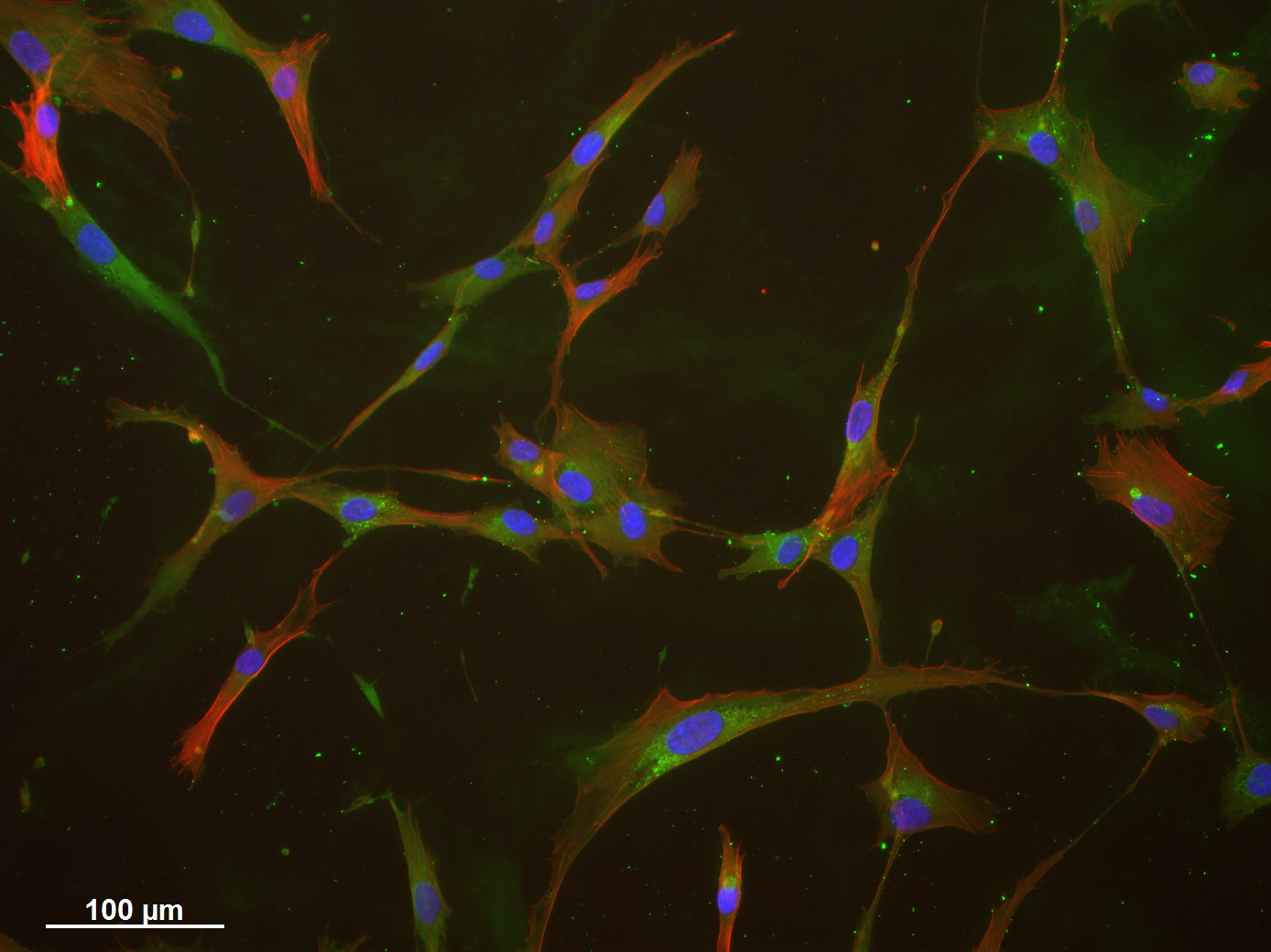

Supplement: S1 File — (ZIP) [file pone.0303106.s001.zip › S1 File/Figure2_TNC_a enviar/ASCs_TNC_Fig2/7d_m5_(c1+c2+c3).JPG]

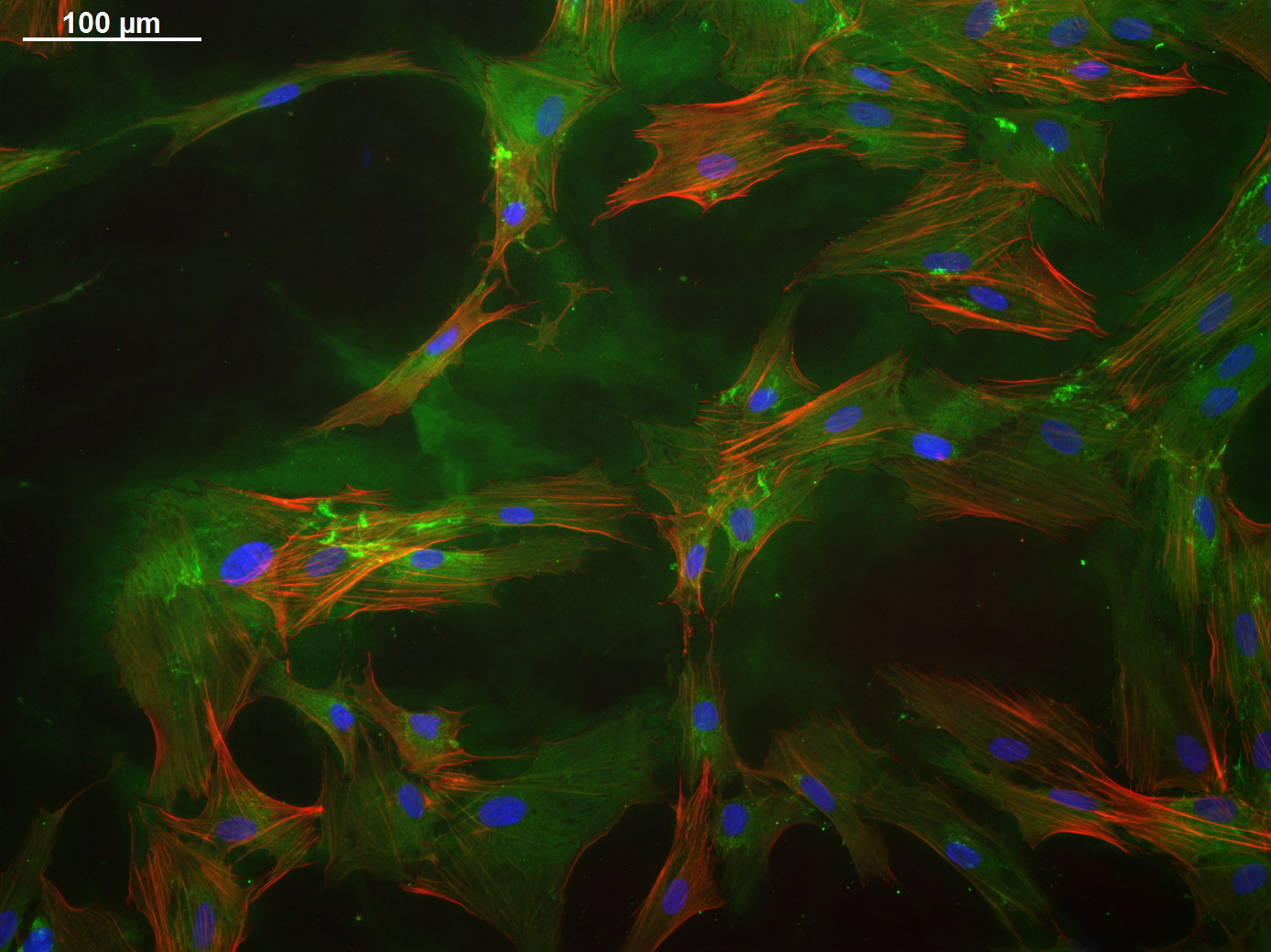

Supplement: S1 File — (ZIP) [file pone.0303106.s001.zip › S1 File/Figure2_TNC_a enviar/ASCs_TNC_Fig2/7d_m6_(c1+c2+c3).JPG]
