# Supplementary figures and images for: Correction: Correction: Understanding the Role of Growth Factors in Modulating Stem Cell Tenogenesis
Source: PLoS One. 2024 May 24;19(5):e0304645. doi: 10.1371/journal.pone.0304645 (PMC11125457; doi:10.1371/journal.pone.0304645)

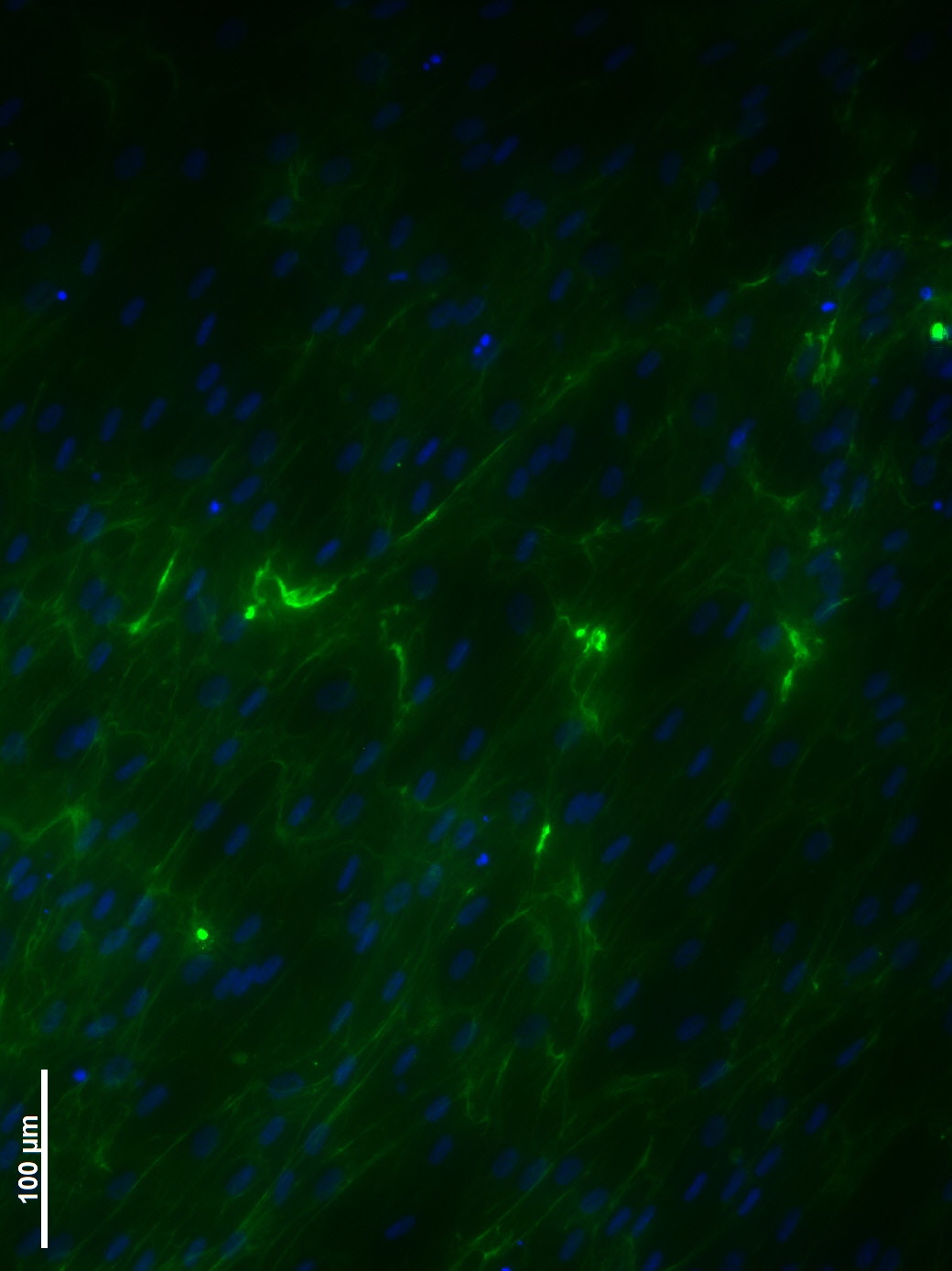

Supplement: S1 File — (ZIP) [file pone.0304645.s001.zip › AFSCs_TNC_Fig2/14d_mA.JPG]

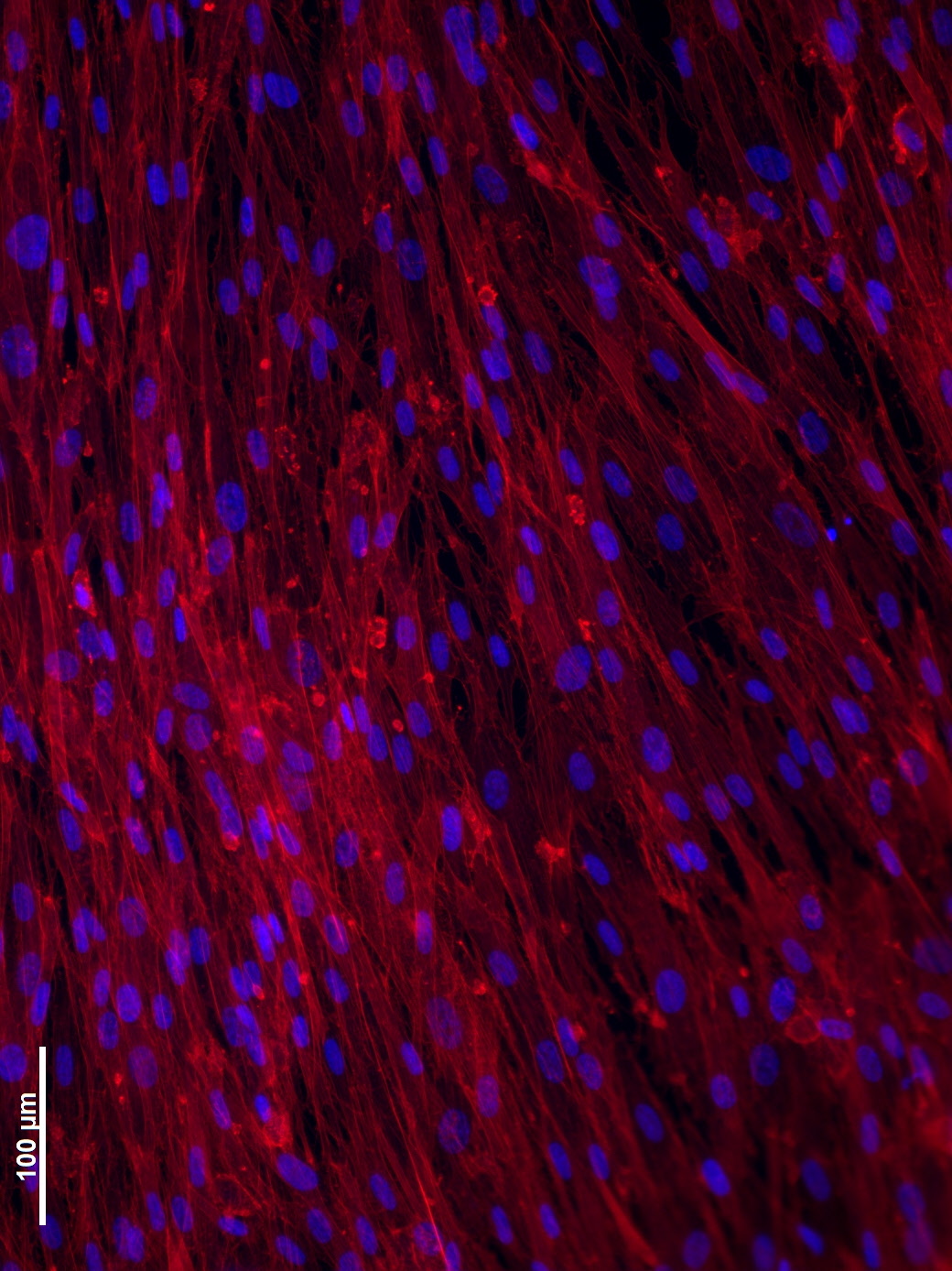

Supplement: S1 File — (ZIP) [file pone.0304645.s001.zip › AFSCs_TNC_Fig2/14d_mB.JPG]

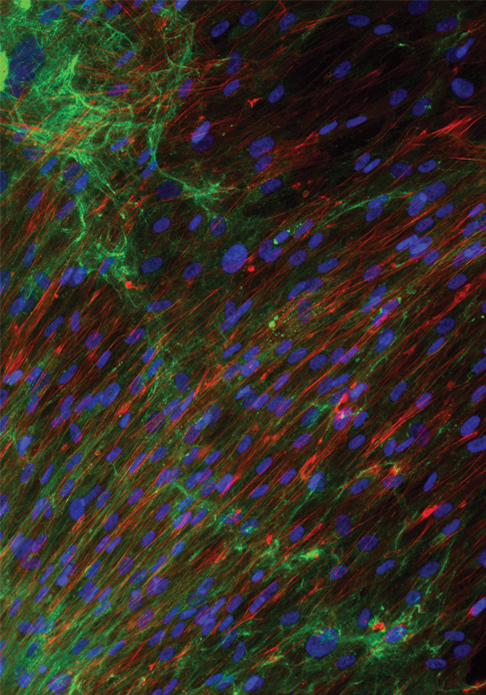

Supplement: S1 File — (ZIP) [file pone.0304645.s001.zip › AFSCs_TNC_Fig2/14d_mEGF.jpg]

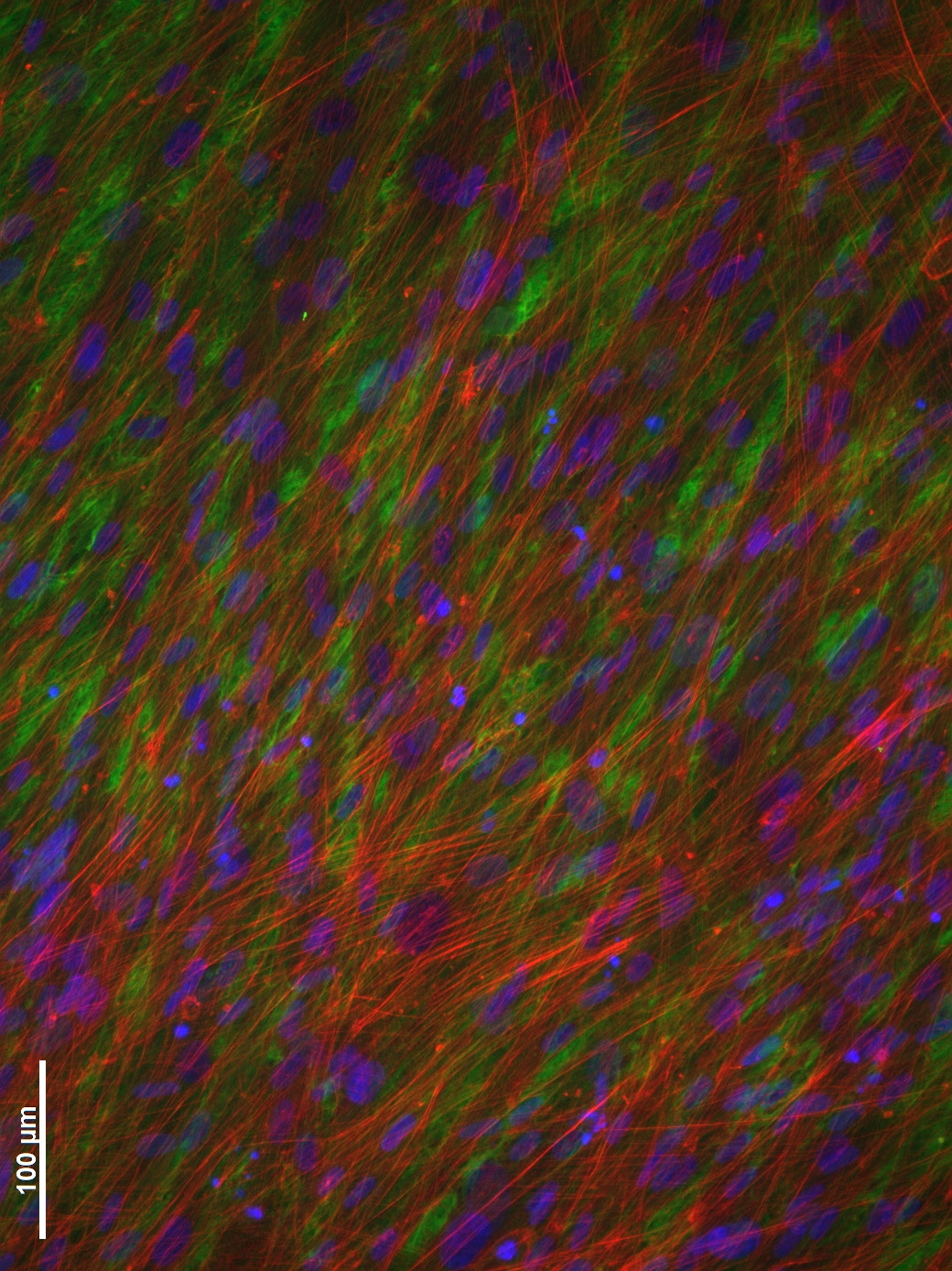

Supplement: S1 File — (ZIP) [file pone.0304645.s001.zip › AFSCs_TNC_Fig2/14d_mFGF.JPG]

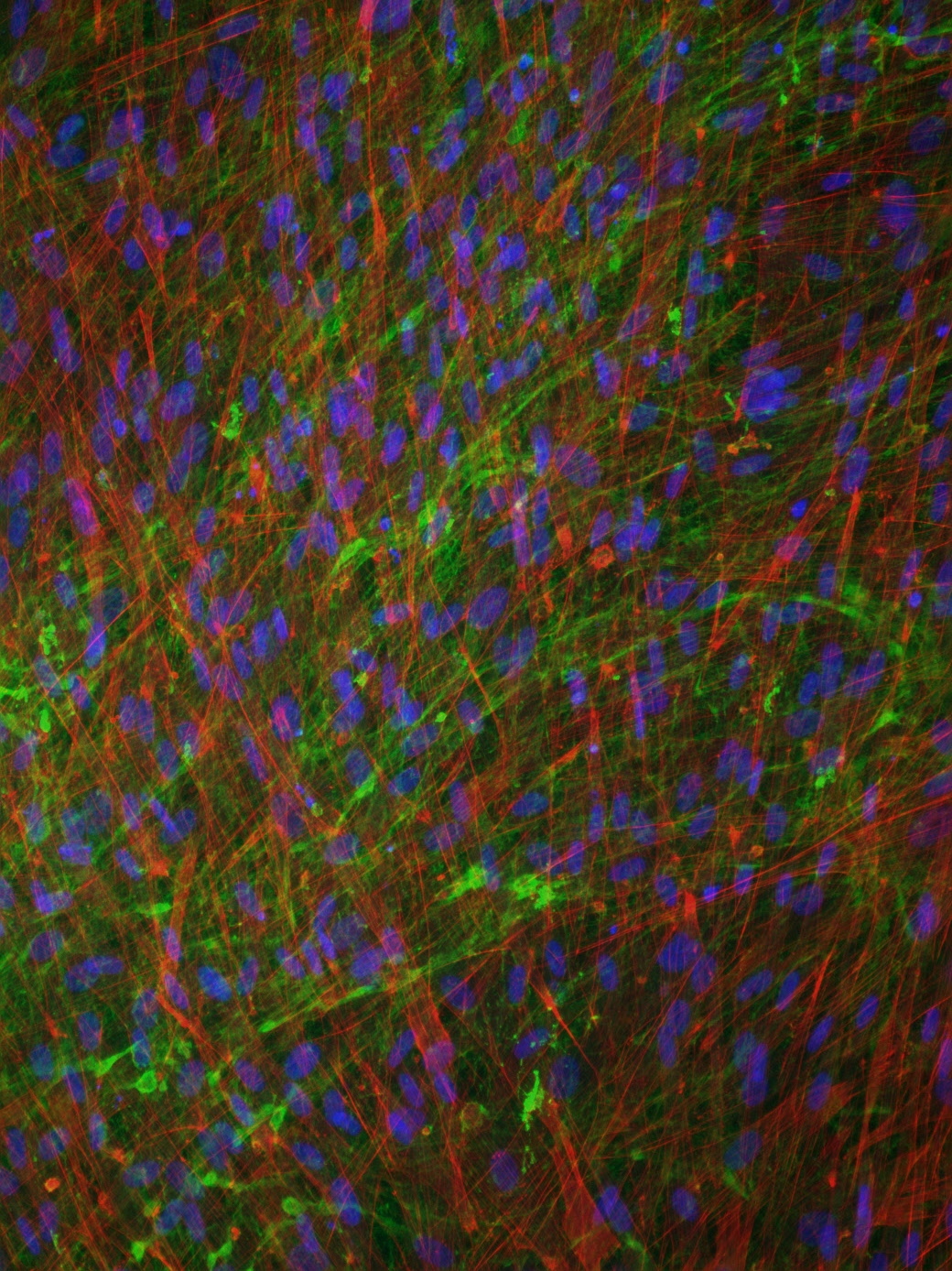

Supplement: S1 File — (ZIP) [file pone.0304645.s001.zip › AFSCs_TNC_Fig2/14d_mPDGF-BB.JPG]

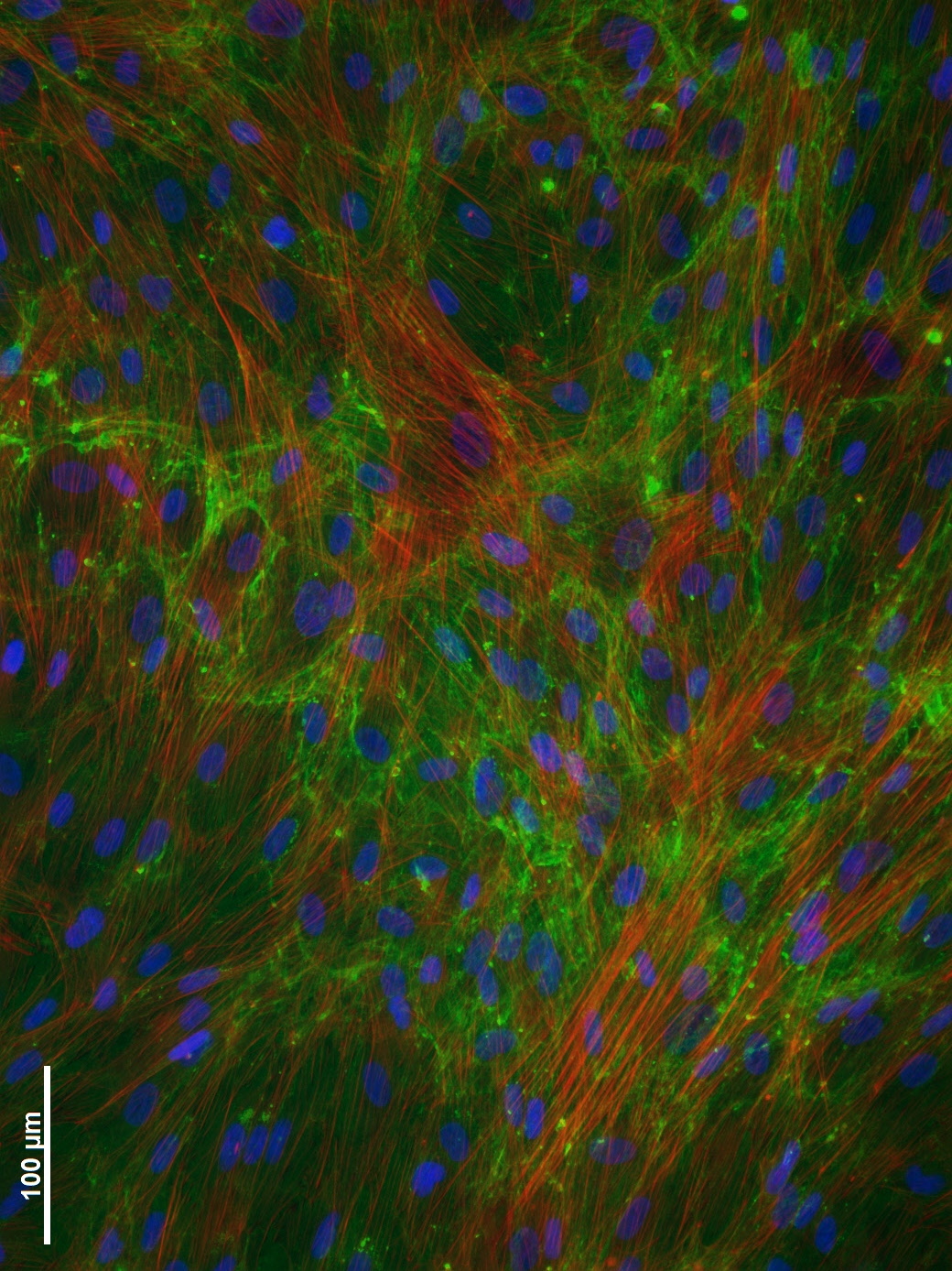

Supplement: S1 File — (ZIP) [file pone.0304645.s001.zip › AFSCs_TNC_Fig2/14d_mTGF-B.JPG]

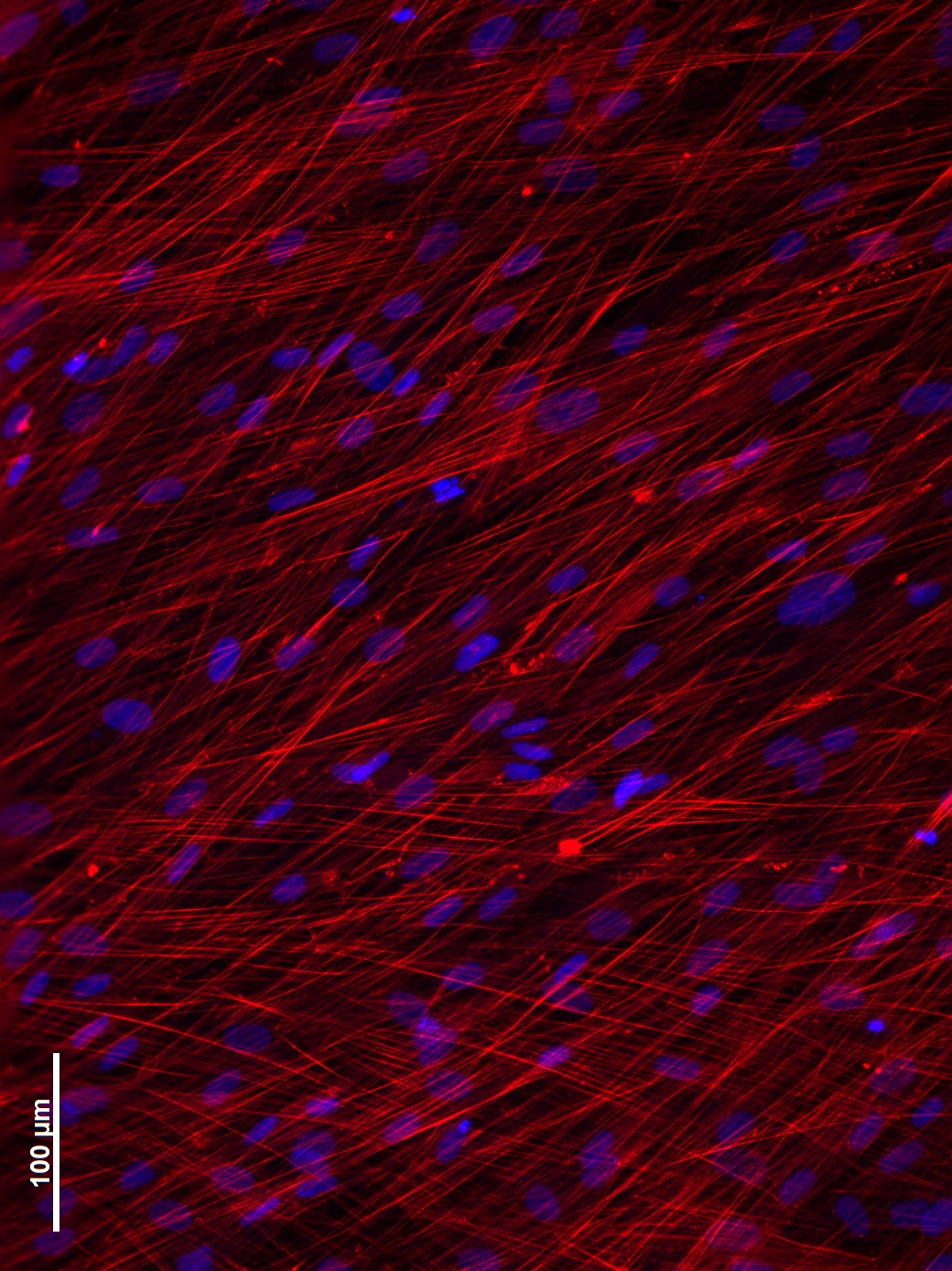

Supplement: S1 File — (ZIP) [file pone.0304645.s001.zip › AFSCs_TNC_Fig2/21d_mA.JPG]

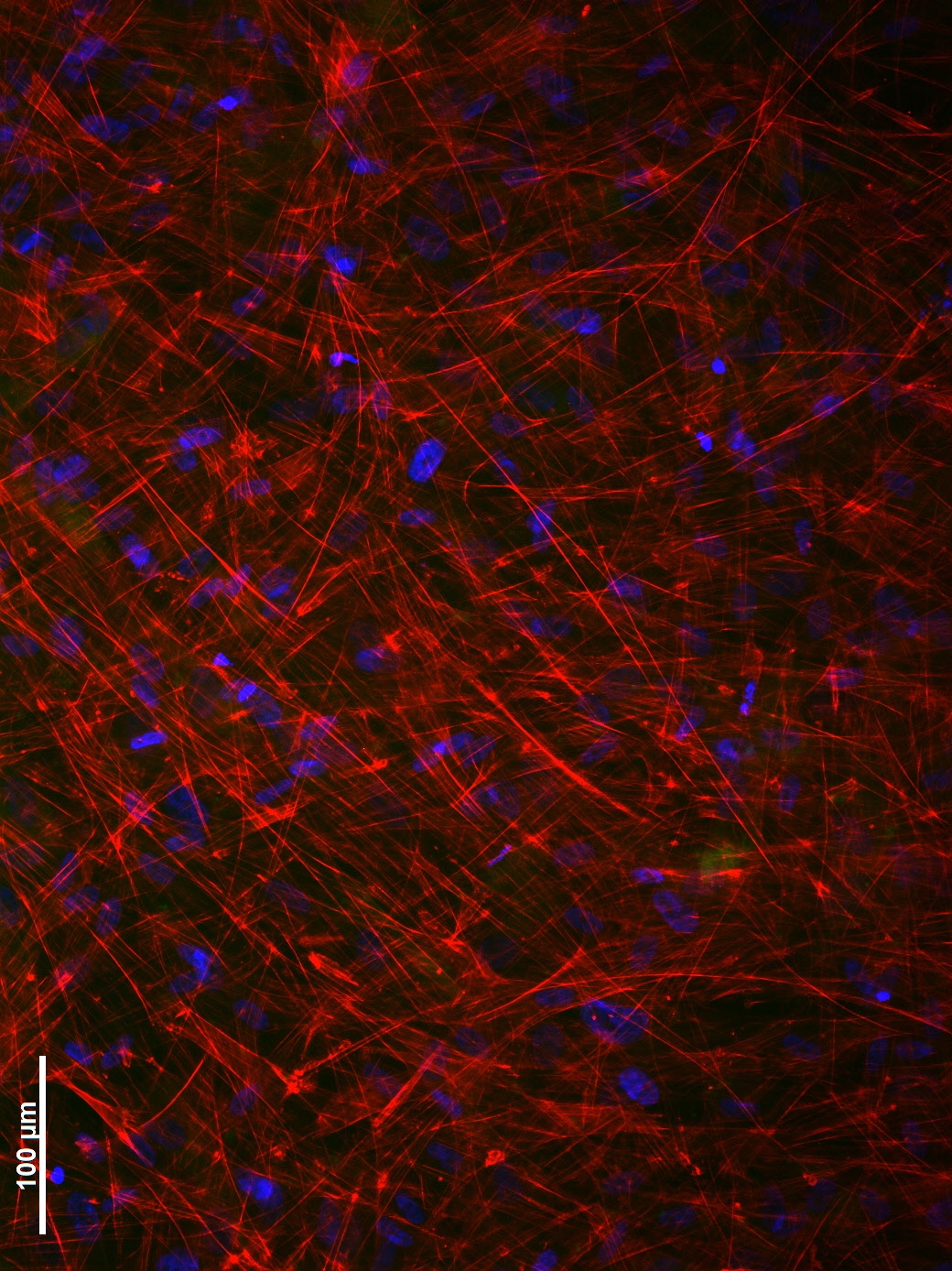

Supplement: S1 File — (ZIP) [file pone.0304645.s001.zip › AFSCs_TNC_Fig2/21d_mB.JPG]

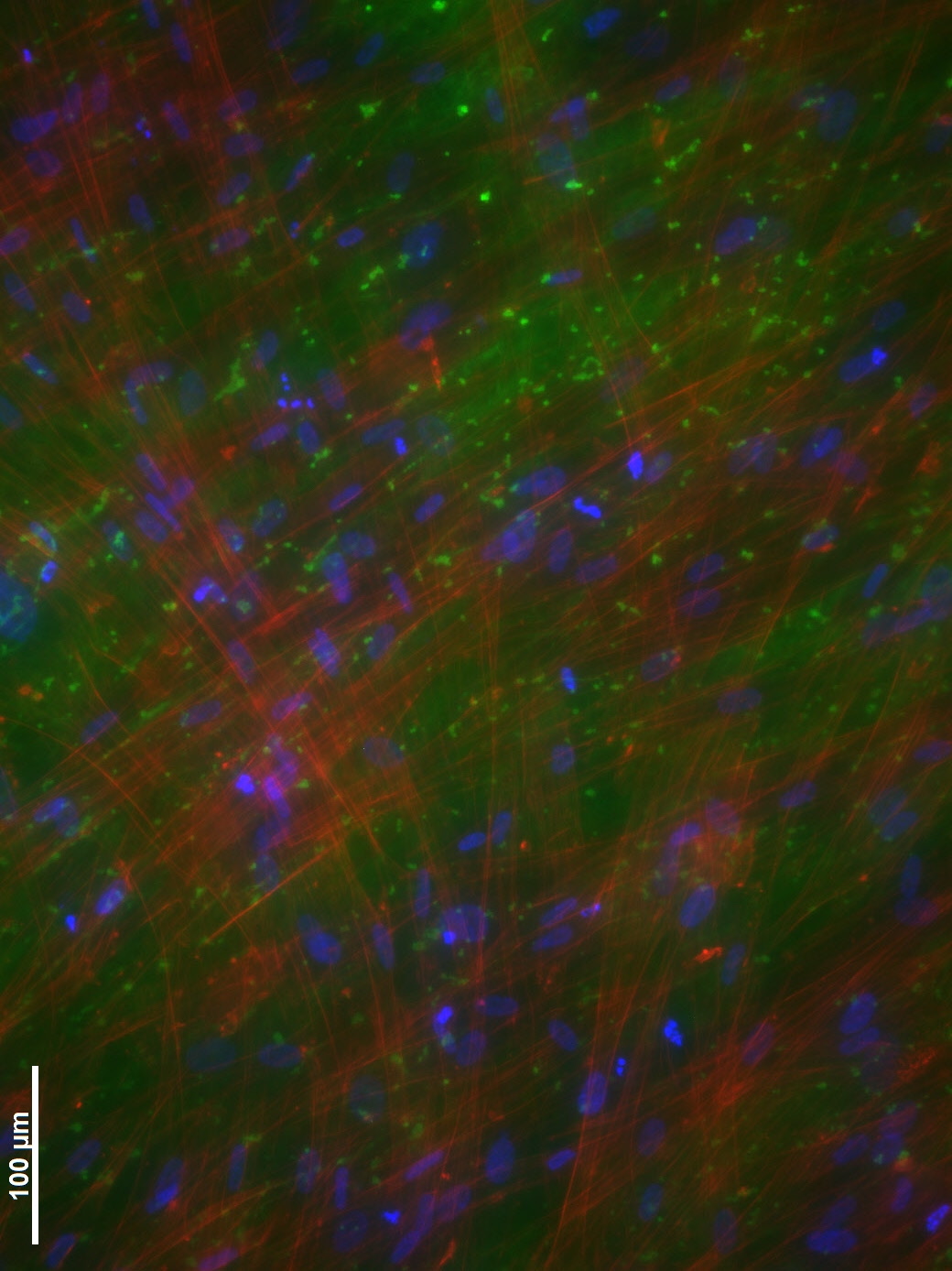

Supplement: S1 File — (ZIP) [file pone.0304645.s001.zip › AFSCs_TNC_Fig2/21d_mEGF.JPG]

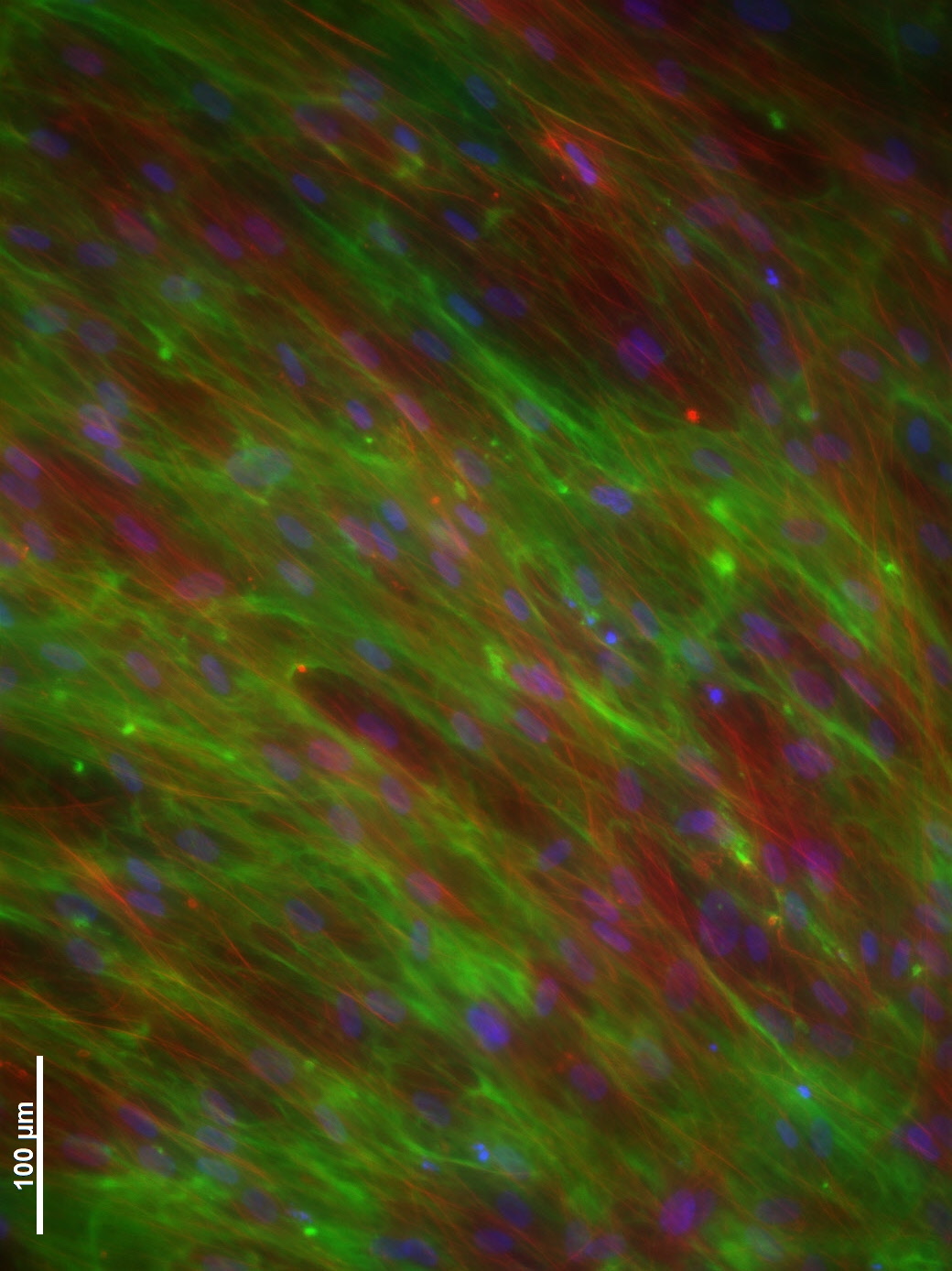

Supplement: S1 File — (ZIP) [file pone.0304645.s001.zip › AFSCs_TNC_Fig2/21d_mFGF.JPG]

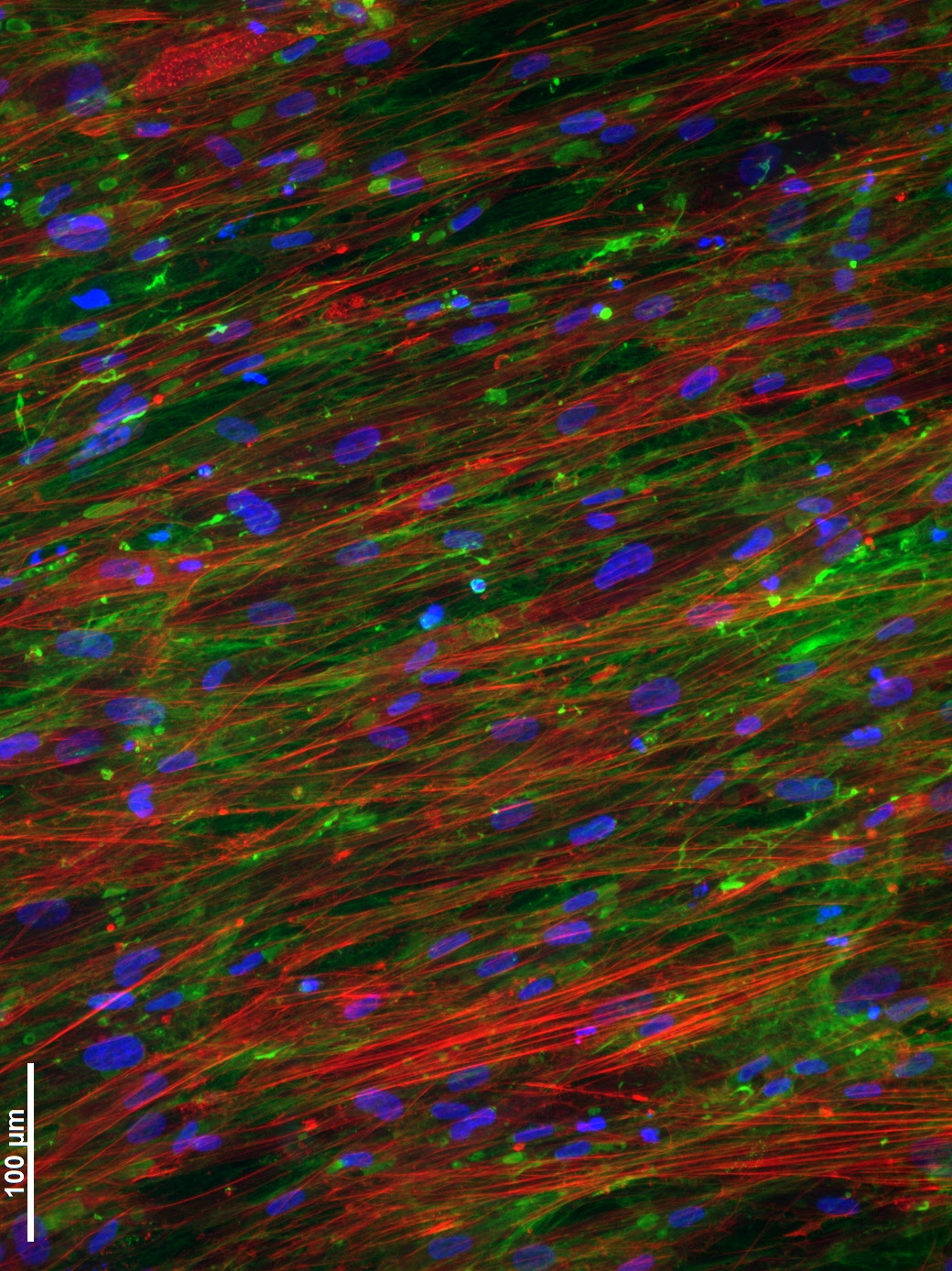

Supplement: S1 File — (ZIP) [file pone.0304645.s001.zip › AFSCs_TNC_Fig2/21d_mPDGF-BB.JPG]

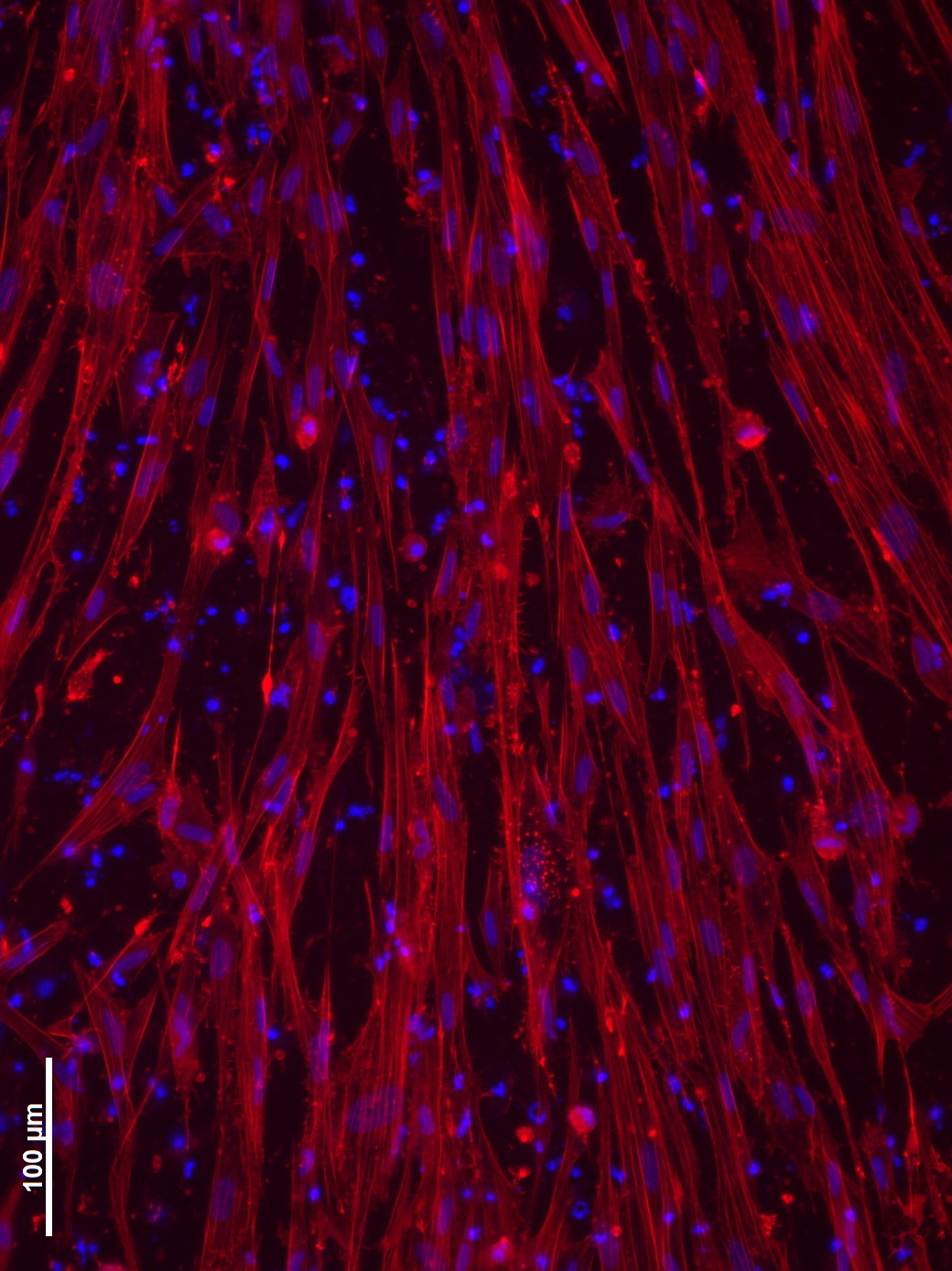

Supplement: S1 File — (ZIP) [file pone.0304645.s001.zip › AFSCs_TNC_Fig2/21d_mTGF-B.JPG]

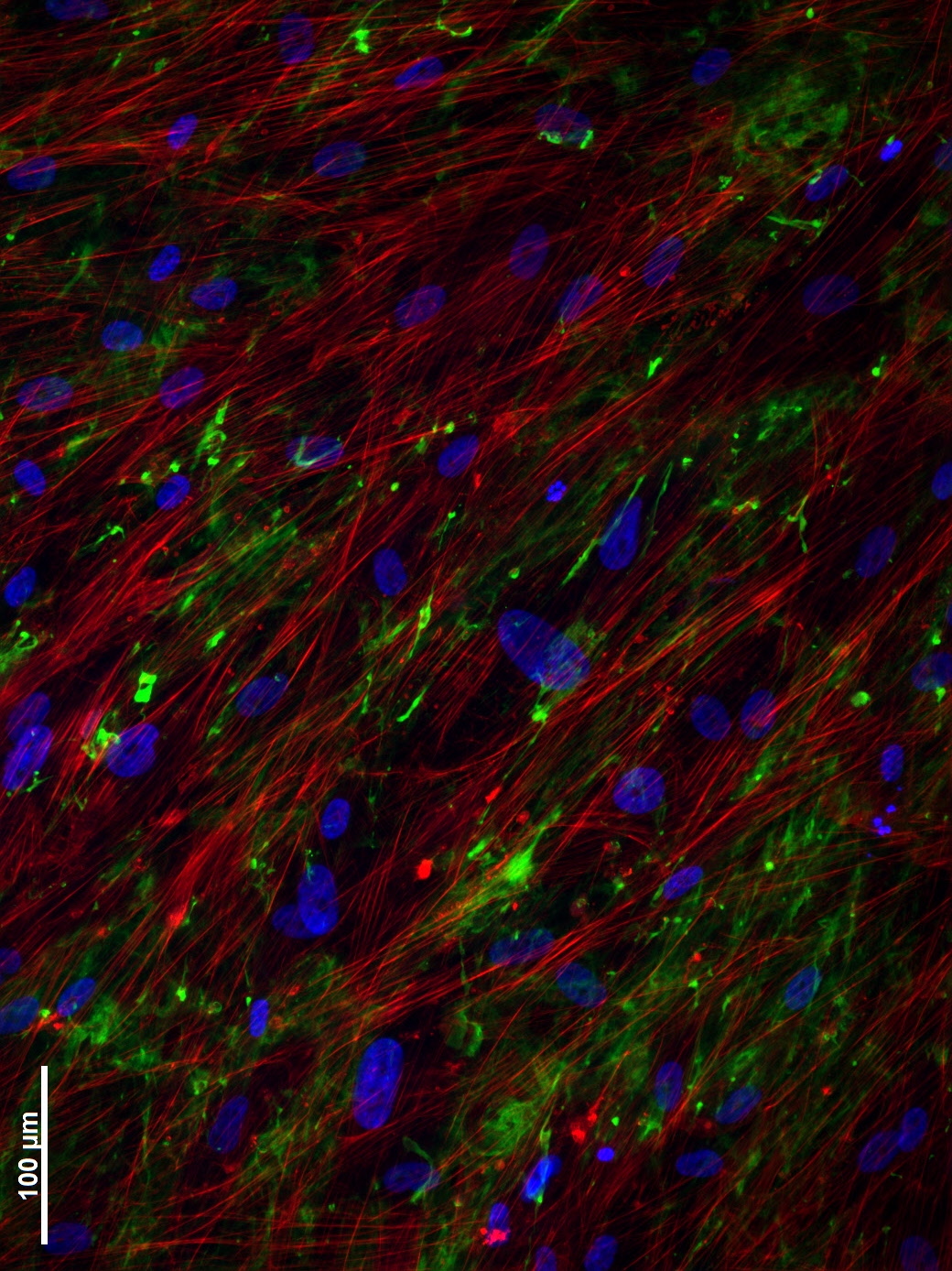

Supplement: S1 File — (ZIP) [file pone.0304645.s001.zip › AFSCs_TNC_Fig2/28d_mA.JPG]

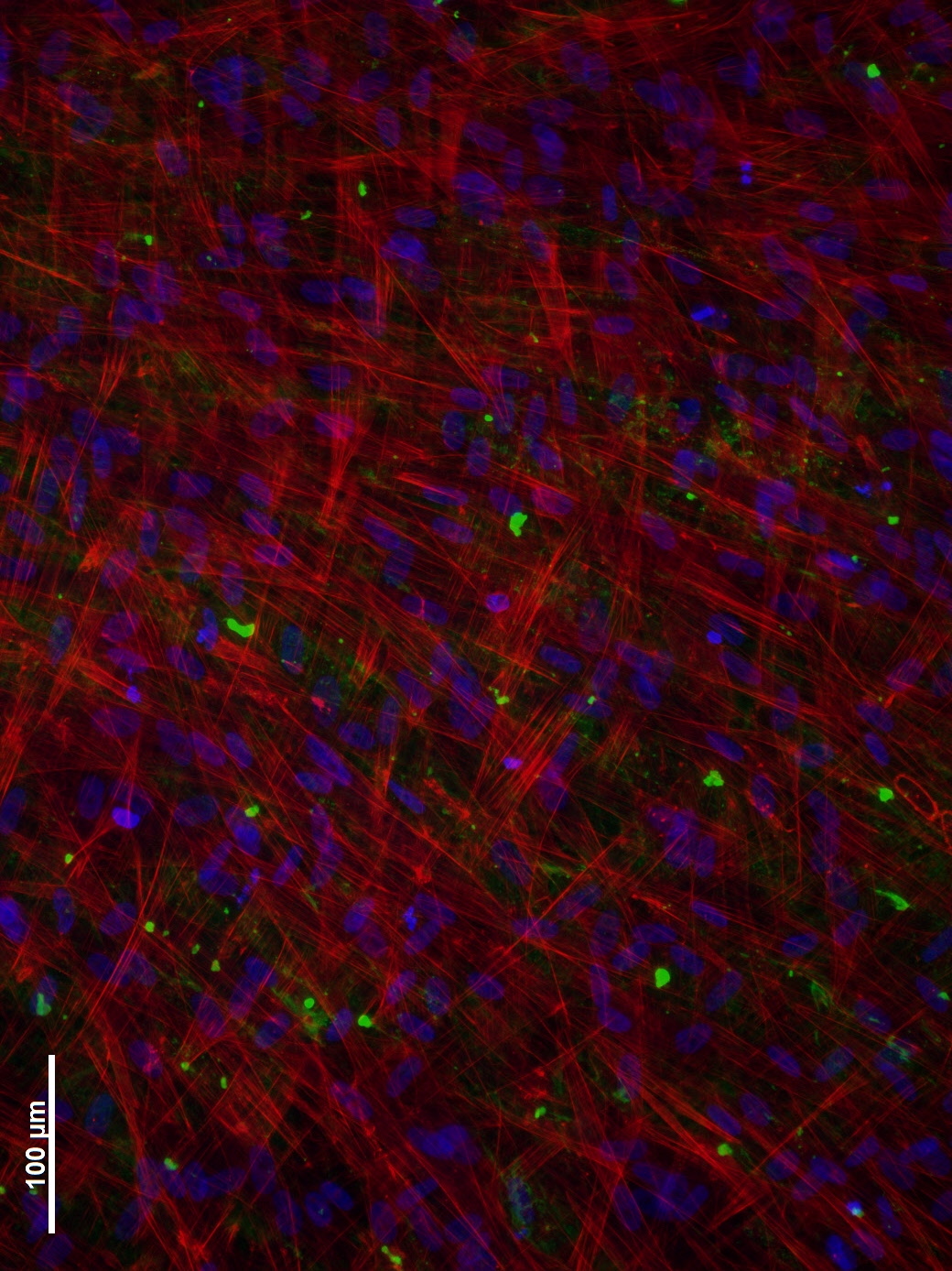

Supplement: S1 File — (ZIP) [file pone.0304645.s001.zip › AFSCs_TNC_Fig2/28d_mB.JPG]

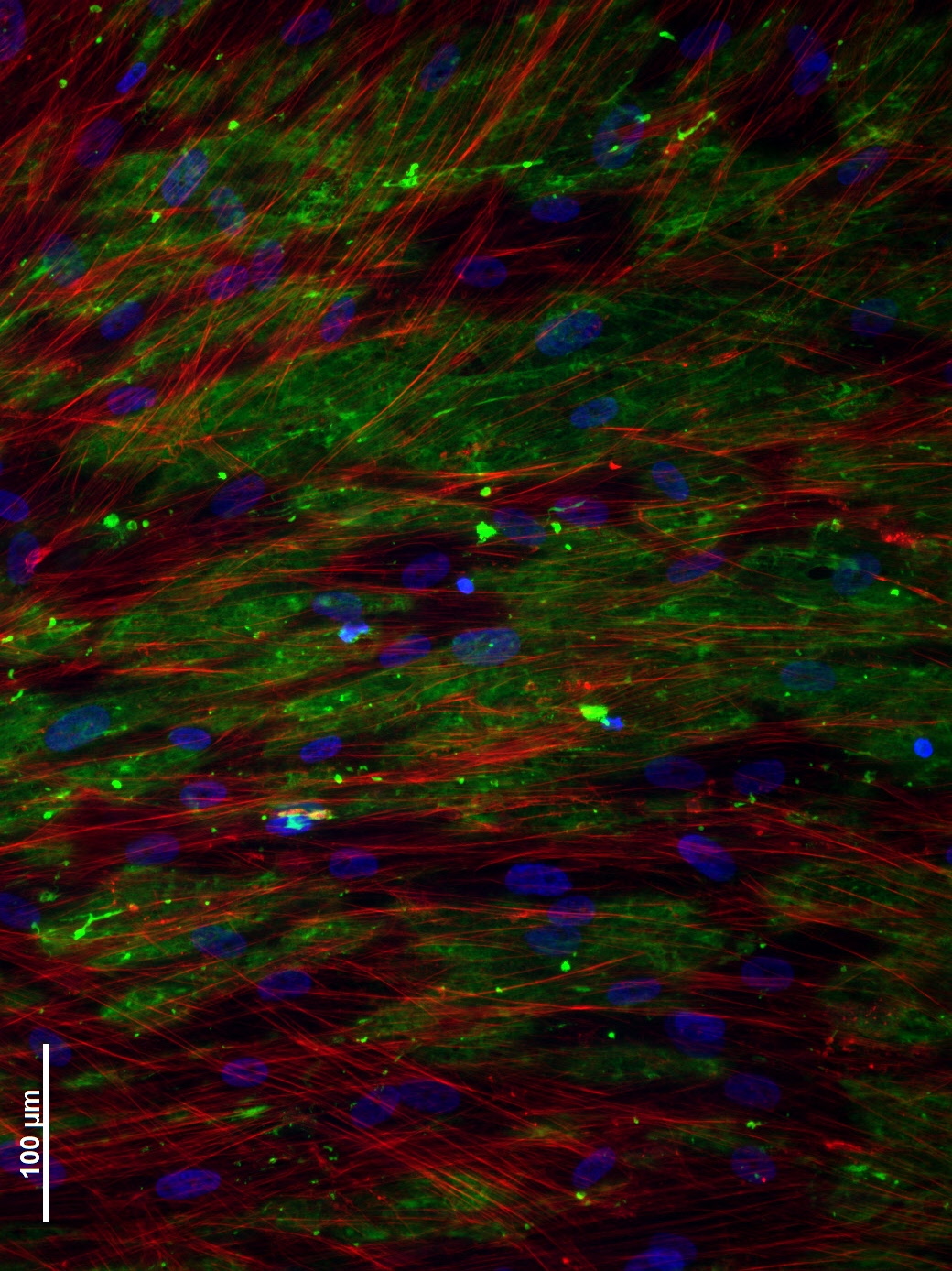

Supplement: S1 File — (ZIP) [file pone.0304645.s001.zip › AFSCs_TNC_Fig2/28d_mEGF.JPG]

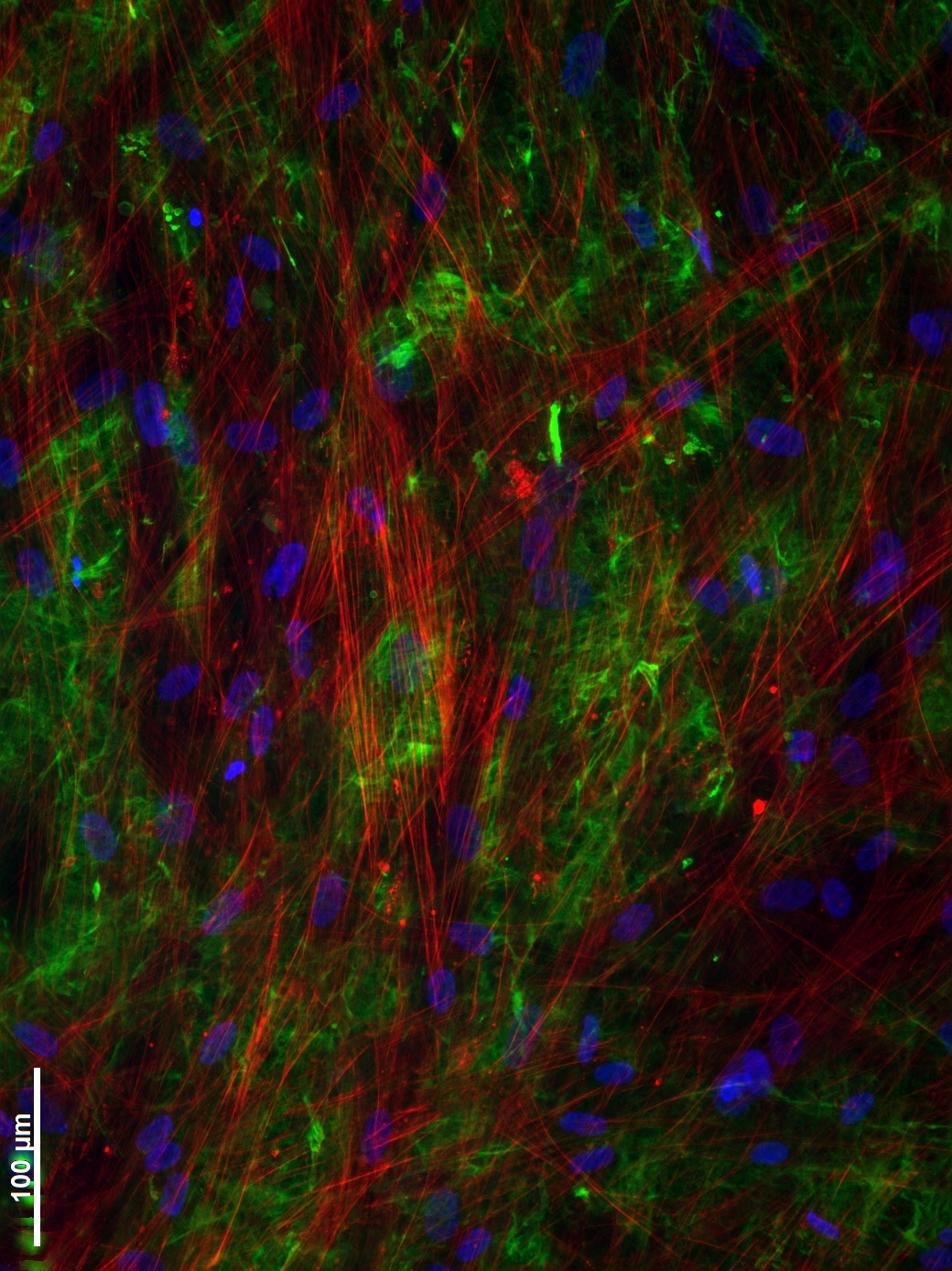

Supplement: S1 File — (ZIP) [file pone.0304645.s001.zip › AFSCs_TNC_Fig2/28d_mFGF.JPG]

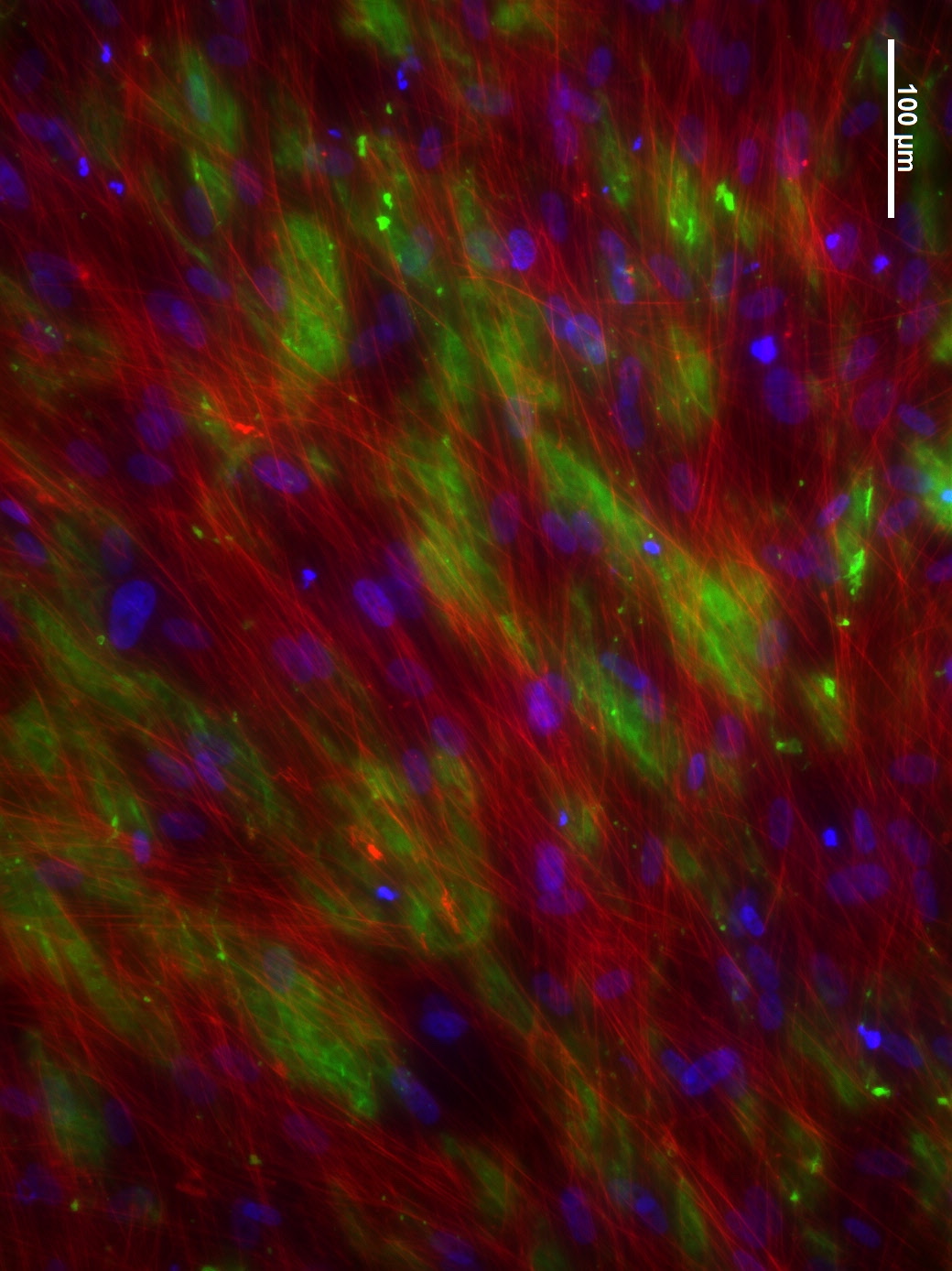

Supplement: S1 File — (ZIP) [file pone.0304645.s001.zip › AFSCs_TNC_Fig2/28d_mPDGF-BB.JPG]

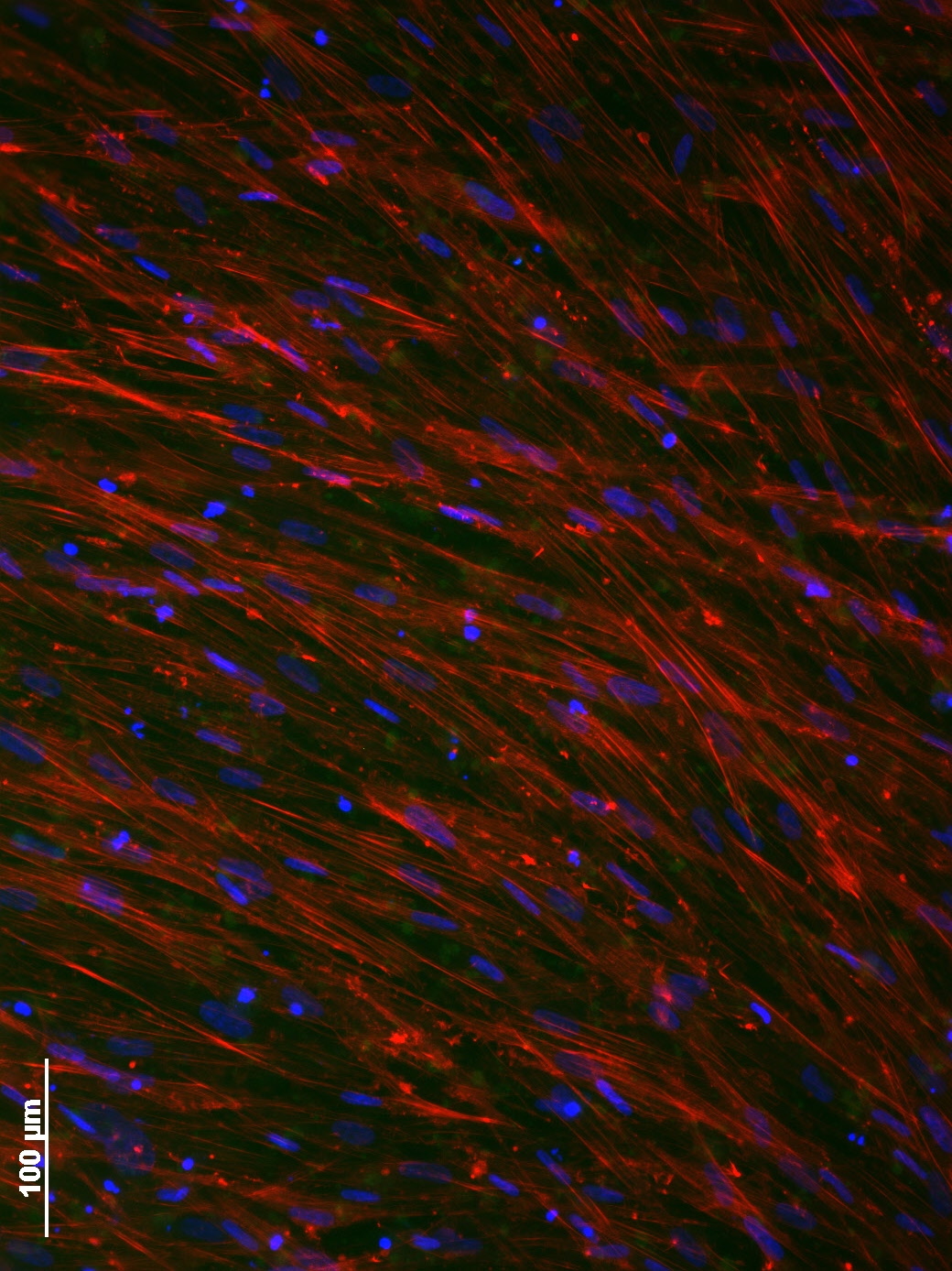

Supplement: S1 File — (ZIP) [file pone.0304645.s001.zip › AFSCs_TNC_Fig2/28d_mTGF-B.jpg]

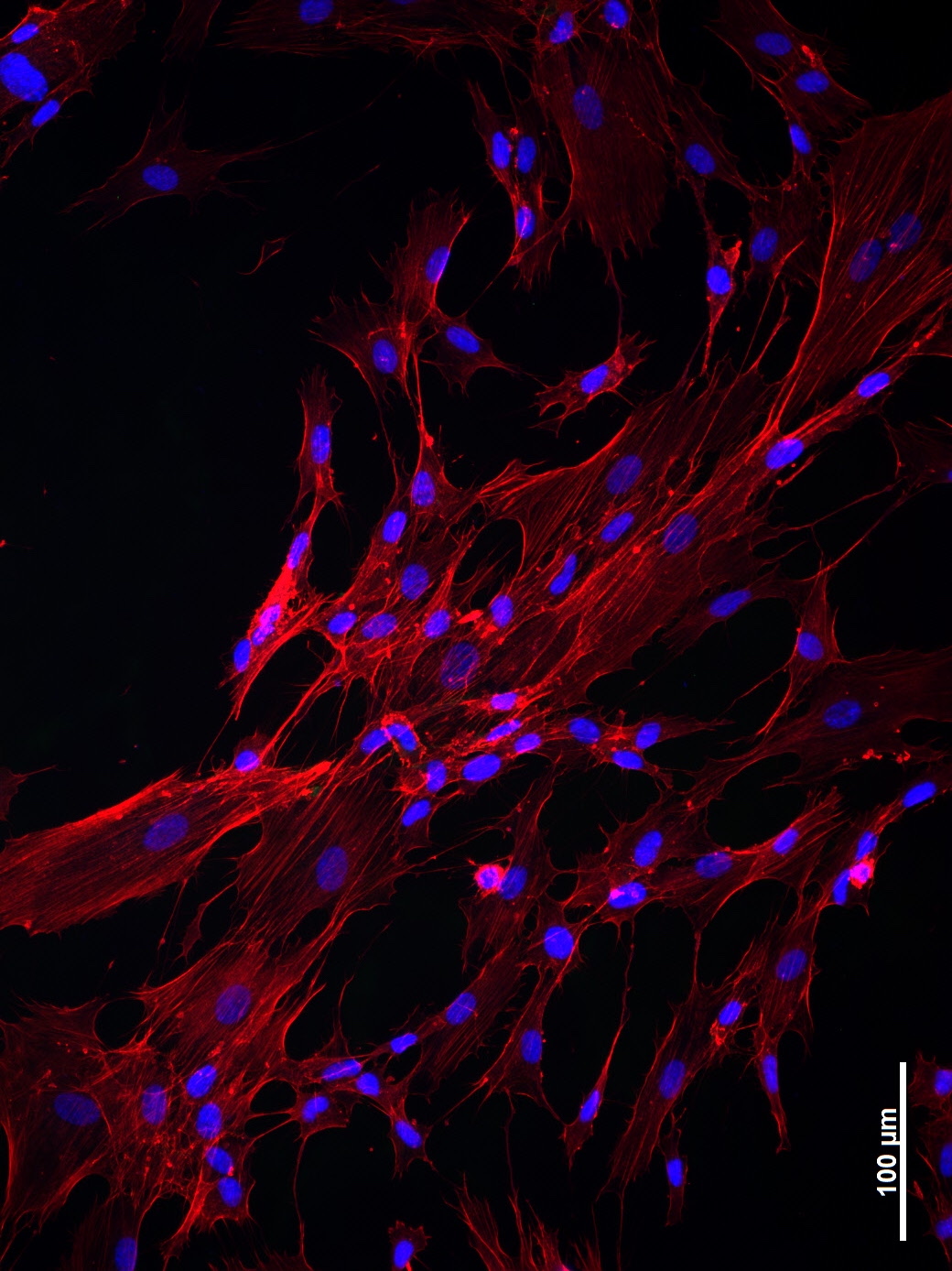

Supplement: S1 File — (ZIP) [file pone.0304645.s001.zip › AFSCs_TNC_Fig2/7d_mA.JPG]

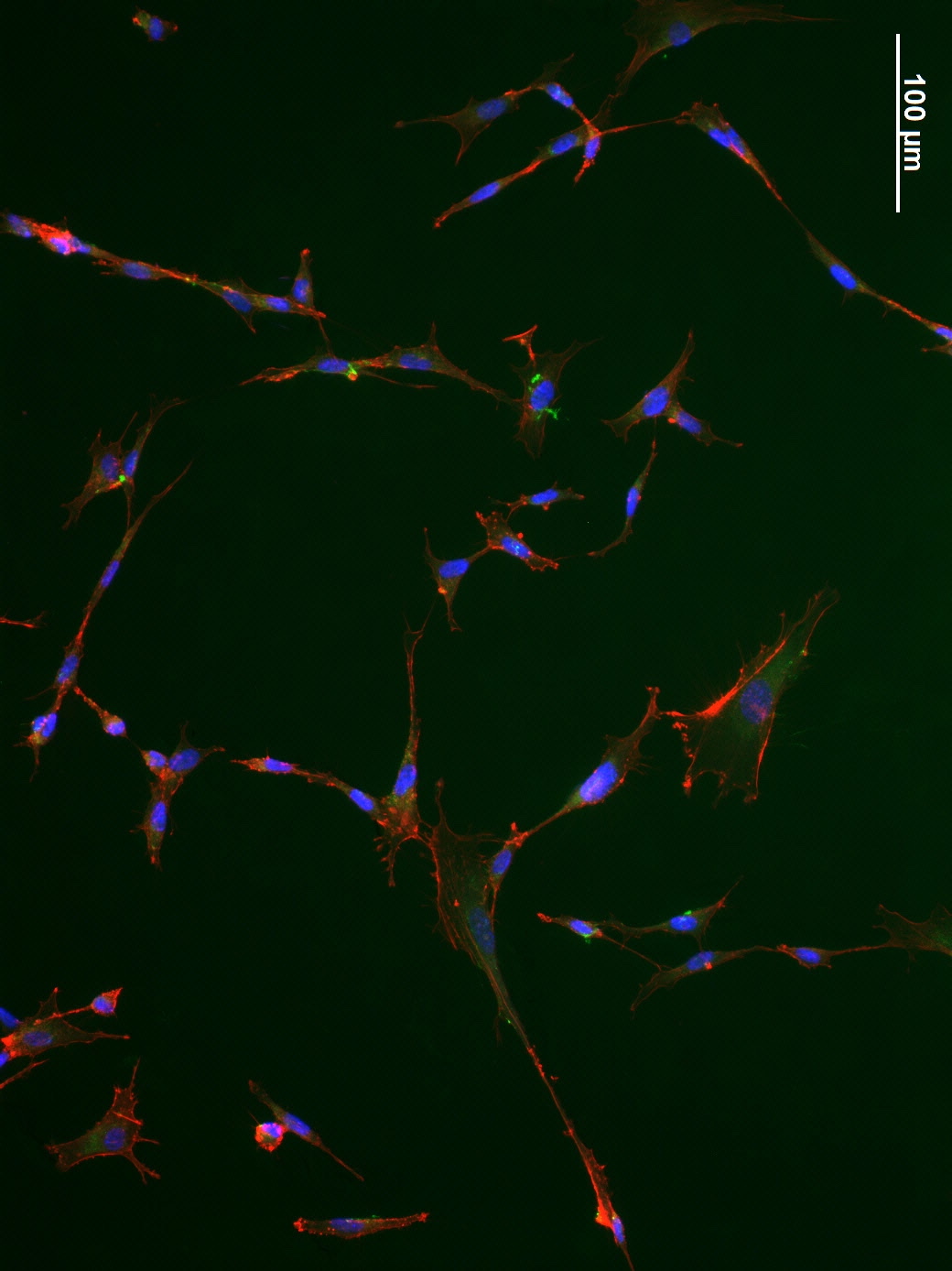

Supplement: S1 File — (ZIP) [file pone.0304645.s001.zip › AFSCs_TNC_Fig2/7d_mB.JPG]

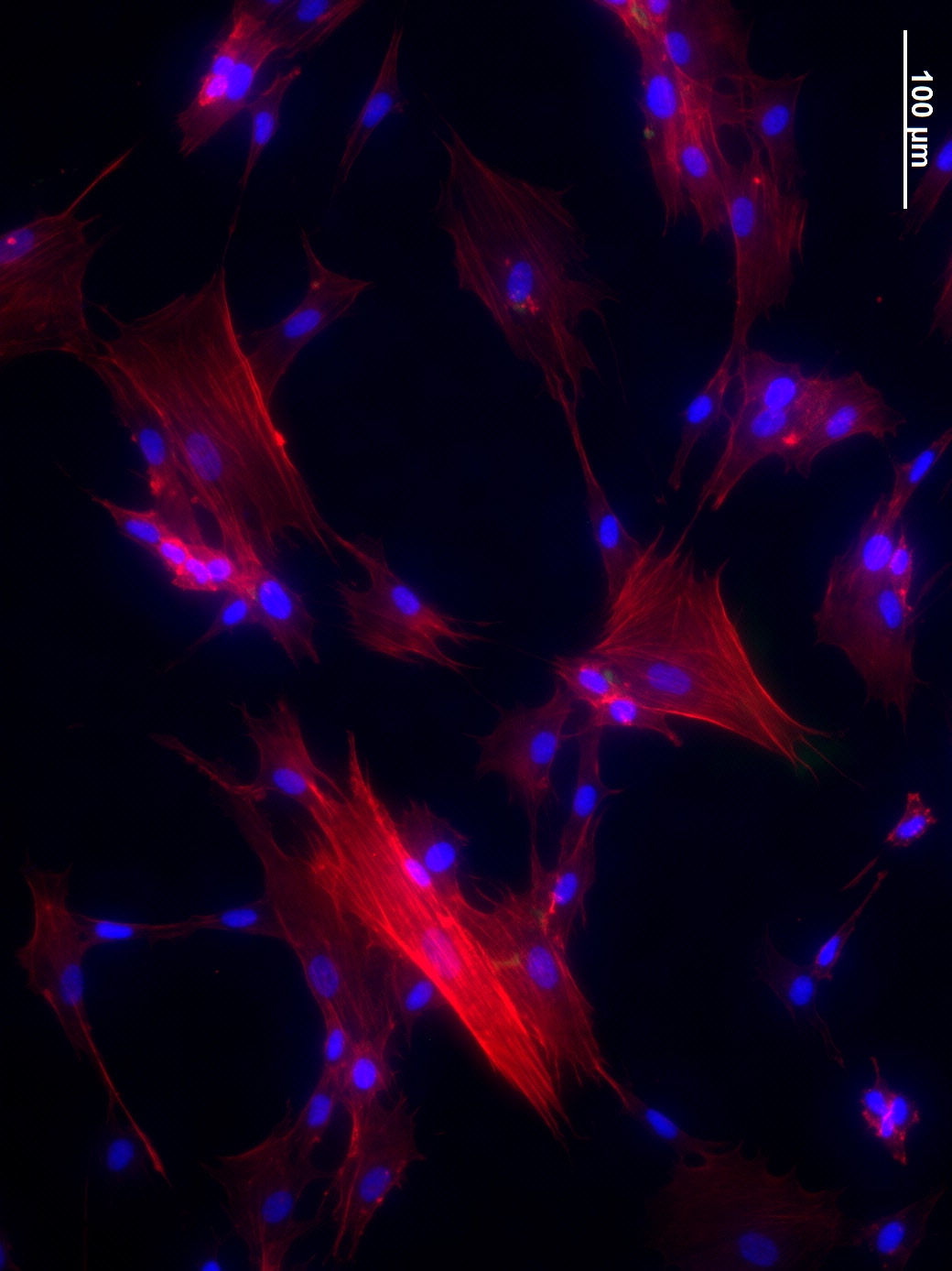

Supplement: S1 File — (ZIP) [file pone.0304645.s001.zip › AFSCs_TNC_Fig2/7d_mEGF.JPG]

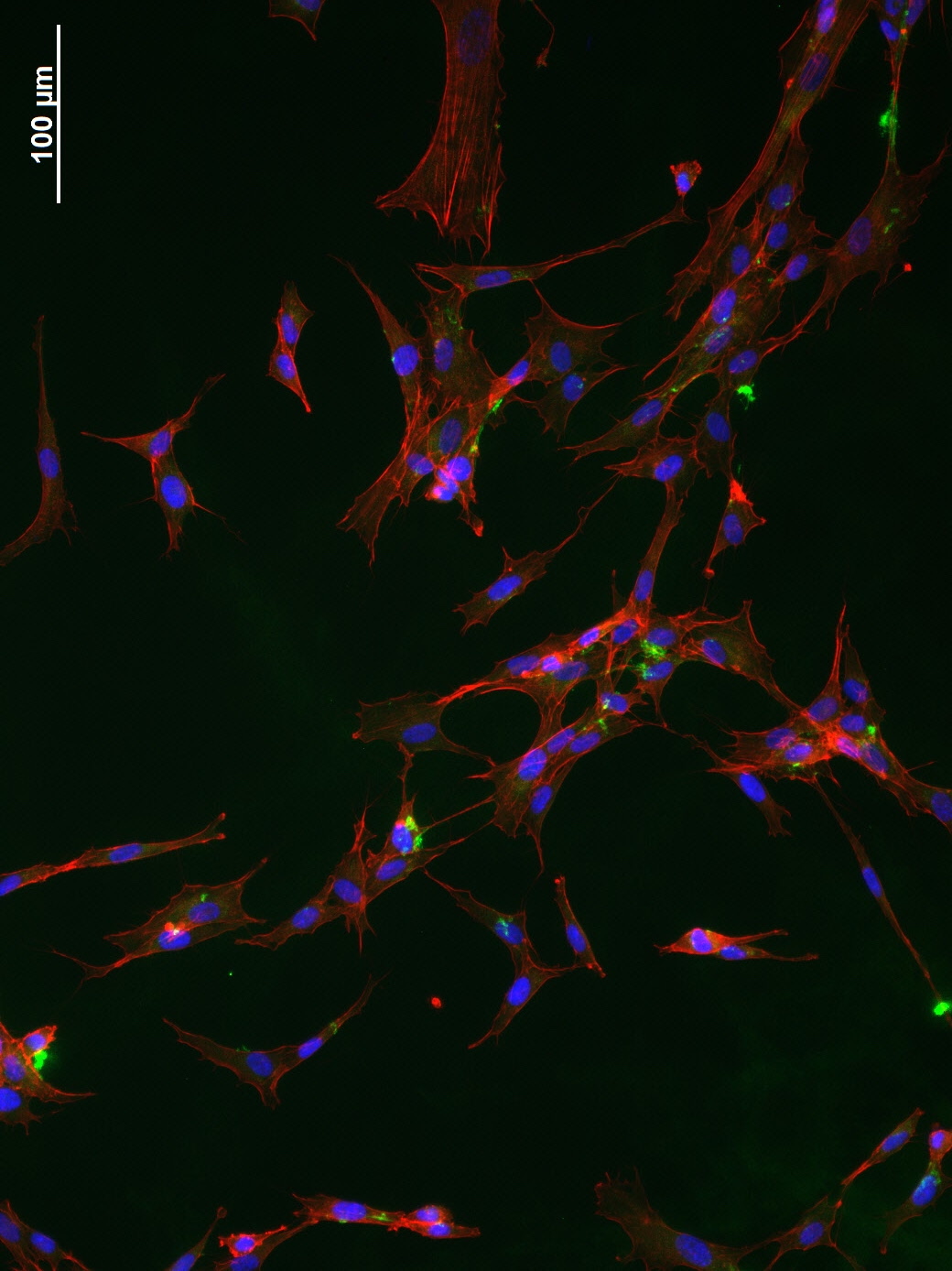

Supplement: S1 File — (ZIP) [file pone.0304645.s001.zip › AFSCs_TNC_Fig2/7d_mFGF.JPG]

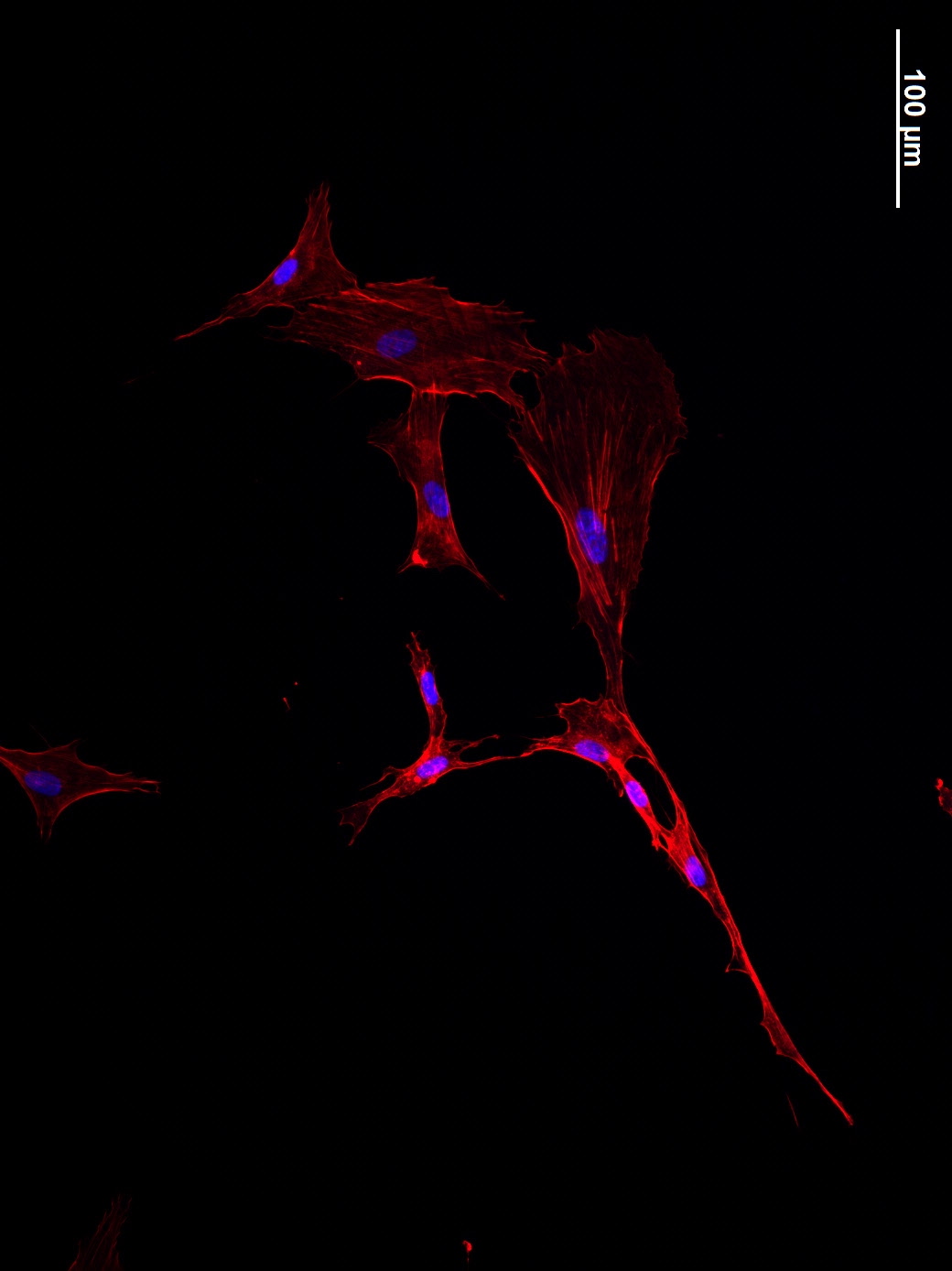

Supplement: S1 File — (ZIP) [file pone.0304645.s001.zip › AFSCs_TNC_Fig2/7d_mPDGF-BB.JPG]

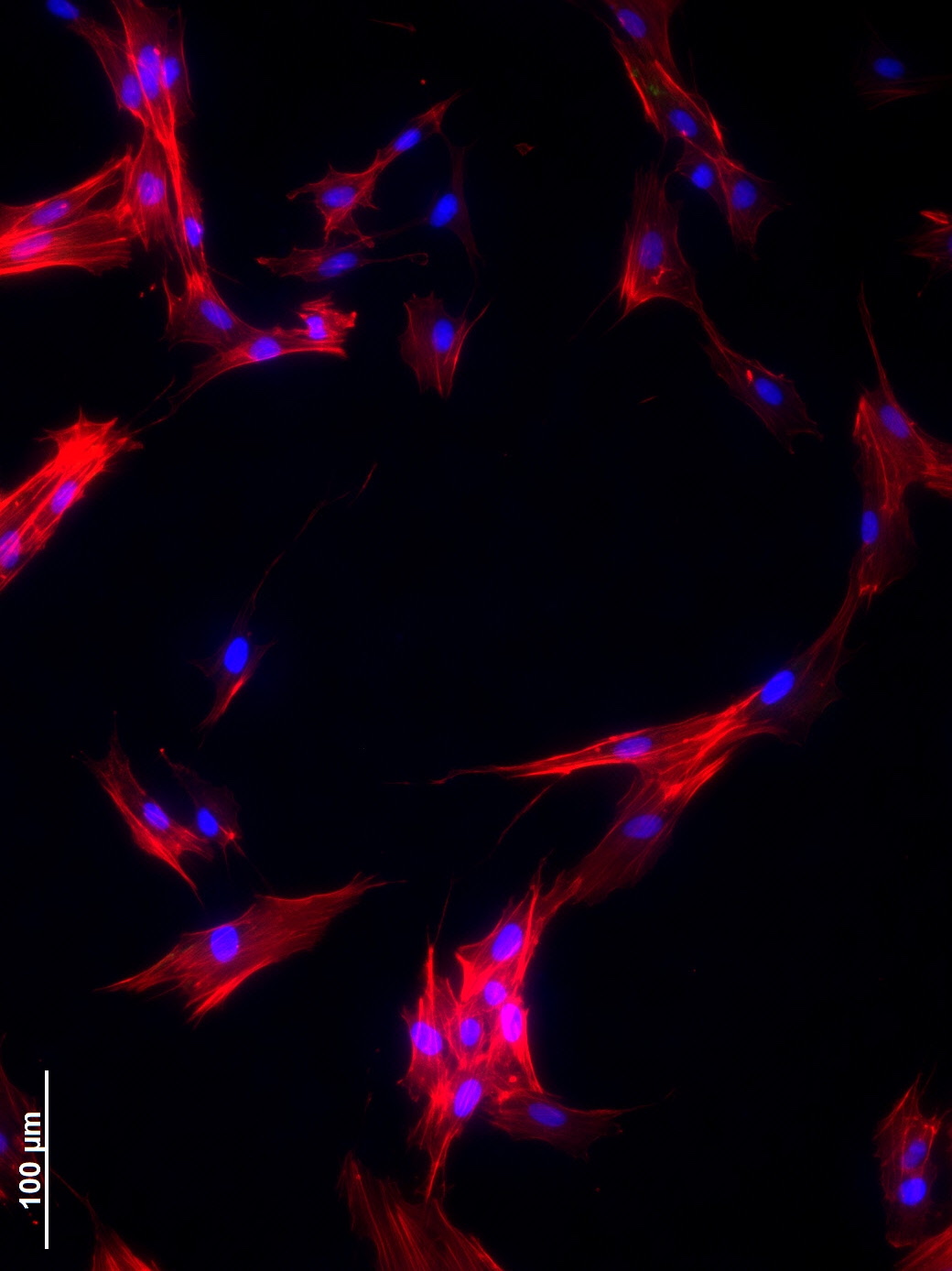

Supplement: S1 File — (ZIP) [file pone.0304645.s001.zip › AFSCs_TNC_Fig2/7d_mTGF-B.JPG]

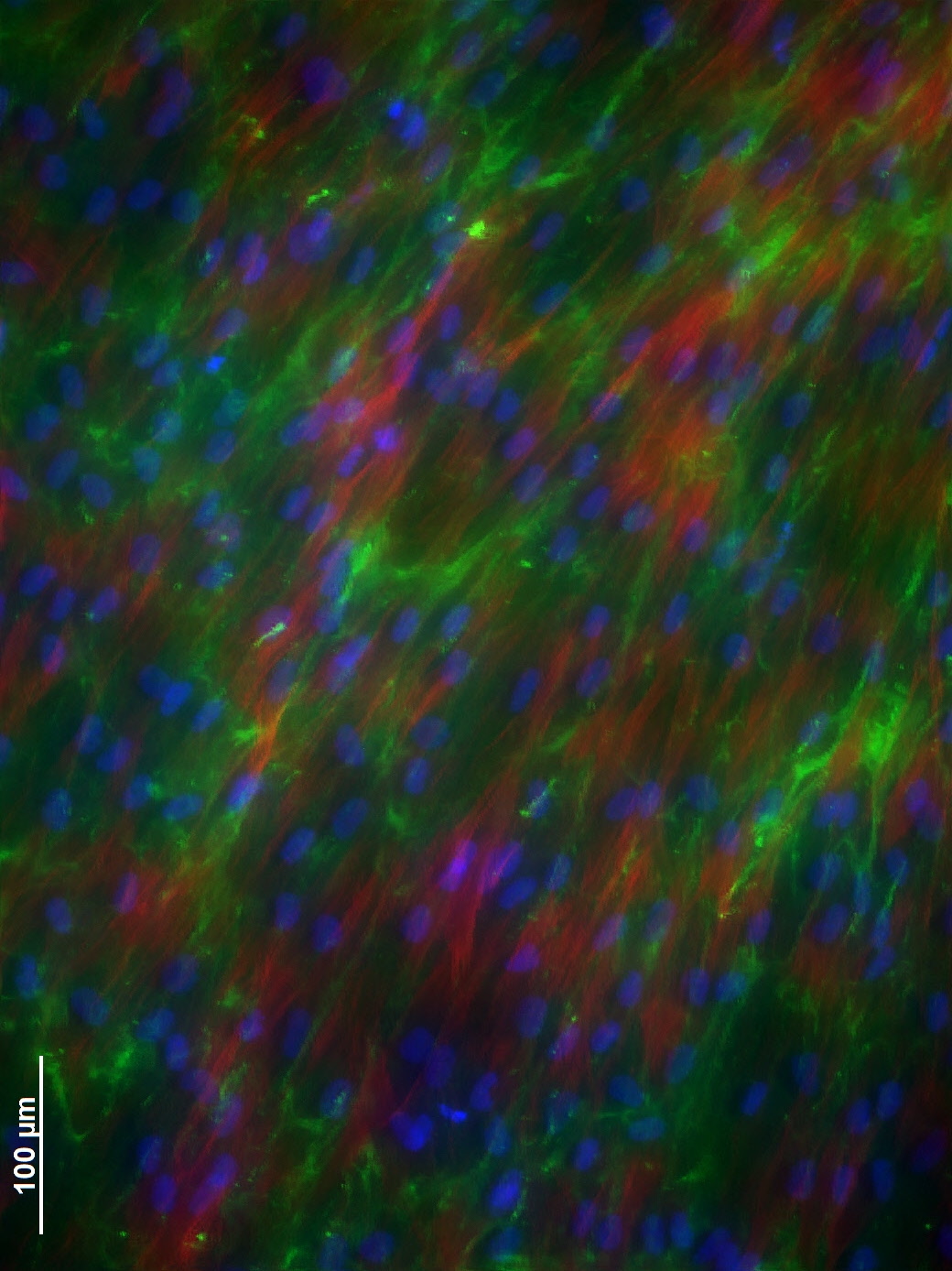

Supplement: S1 File — (ZIP) [file pone.0304645.s001.zip › ASCs_TNC_Fig2/14d_mA.JPG]

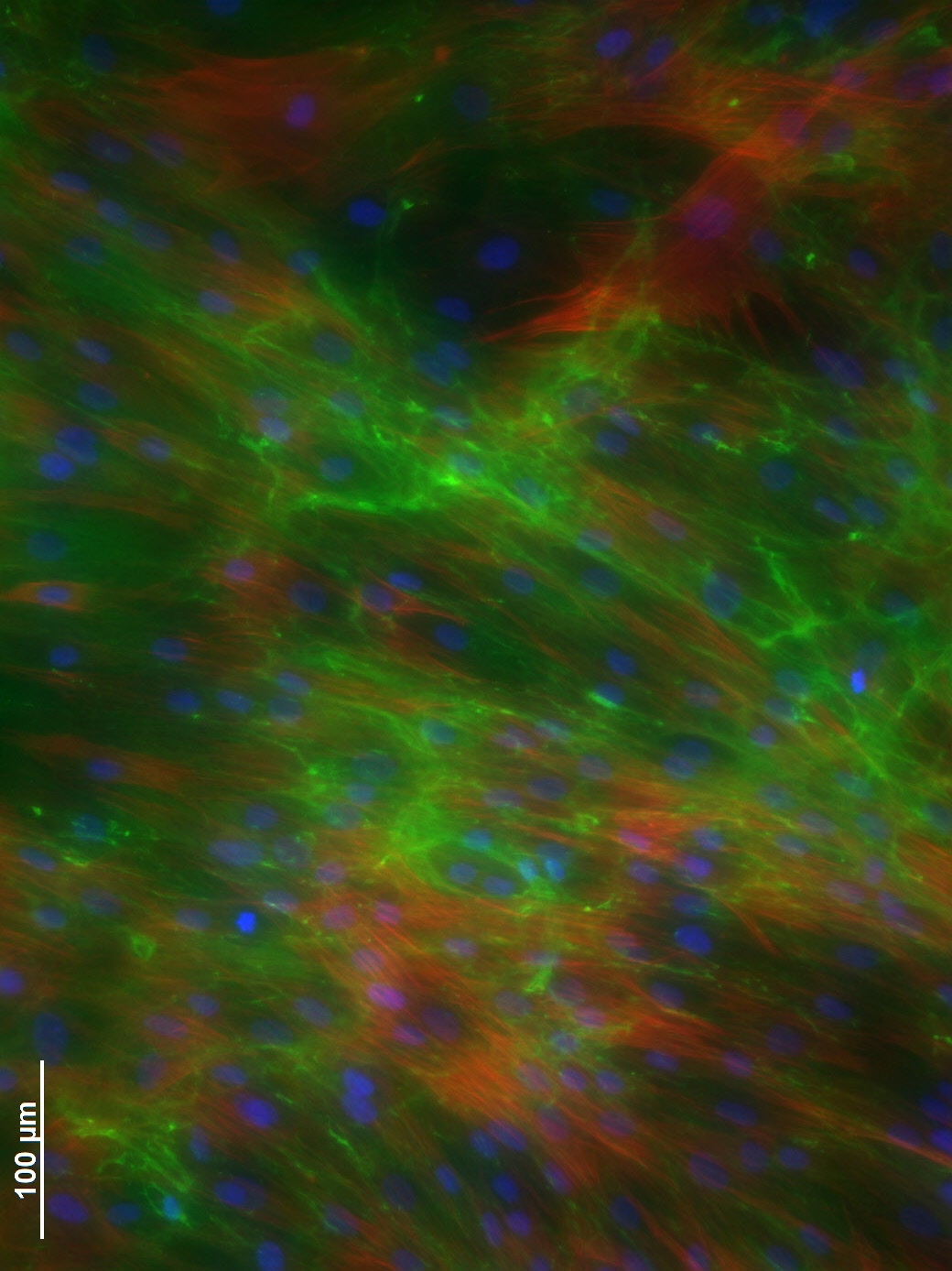

Supplement: S1 File — (ZIP) [file pone.0304645.s001.zip › ASCs_TNC_Fig2/14d_mC.JPG]

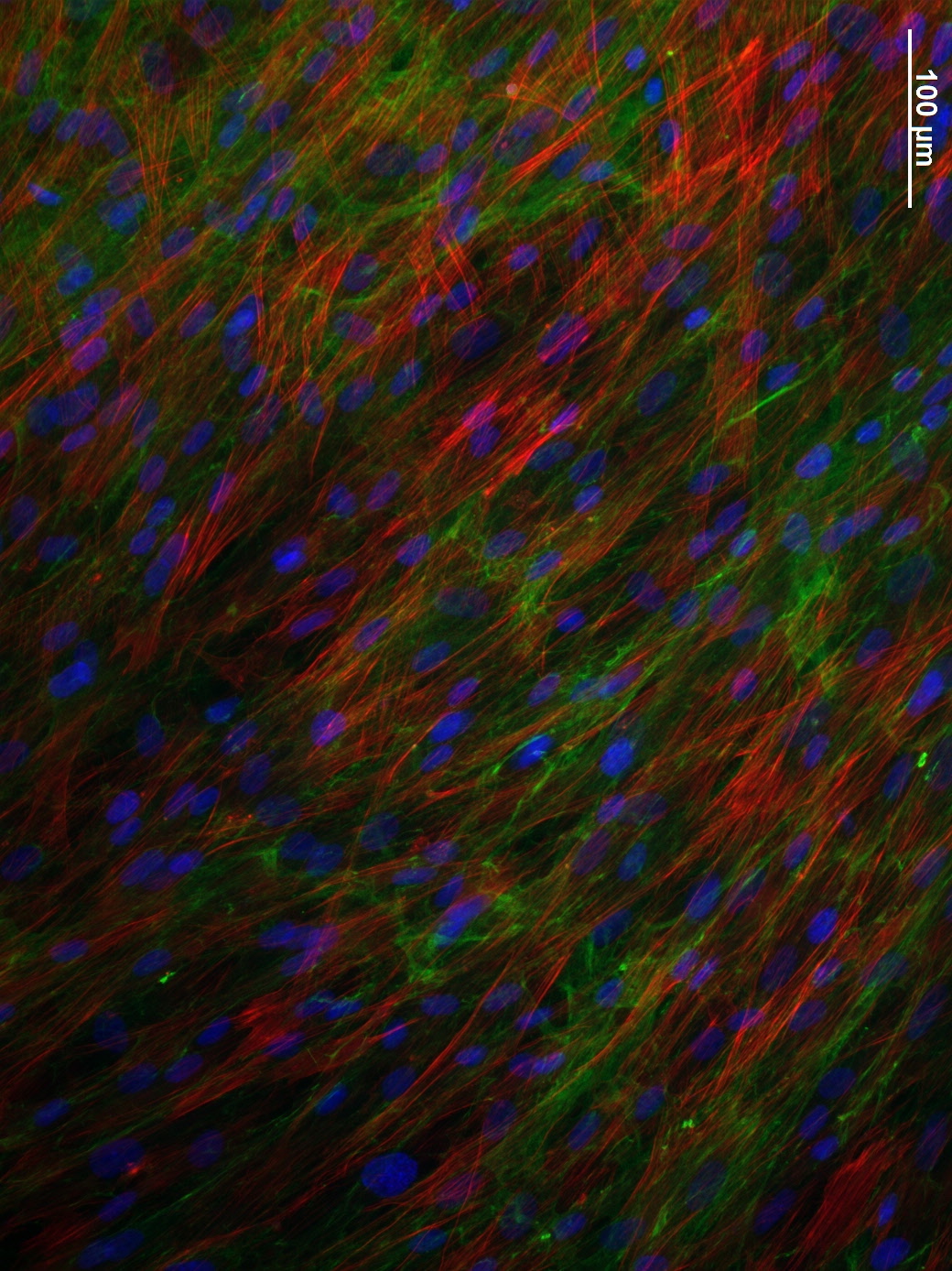

Supplement: S1 File — (ZIP) [file pone.0304645.s001.zip › ASCs_TNC_Fig2/14d_mEGF.JPG]

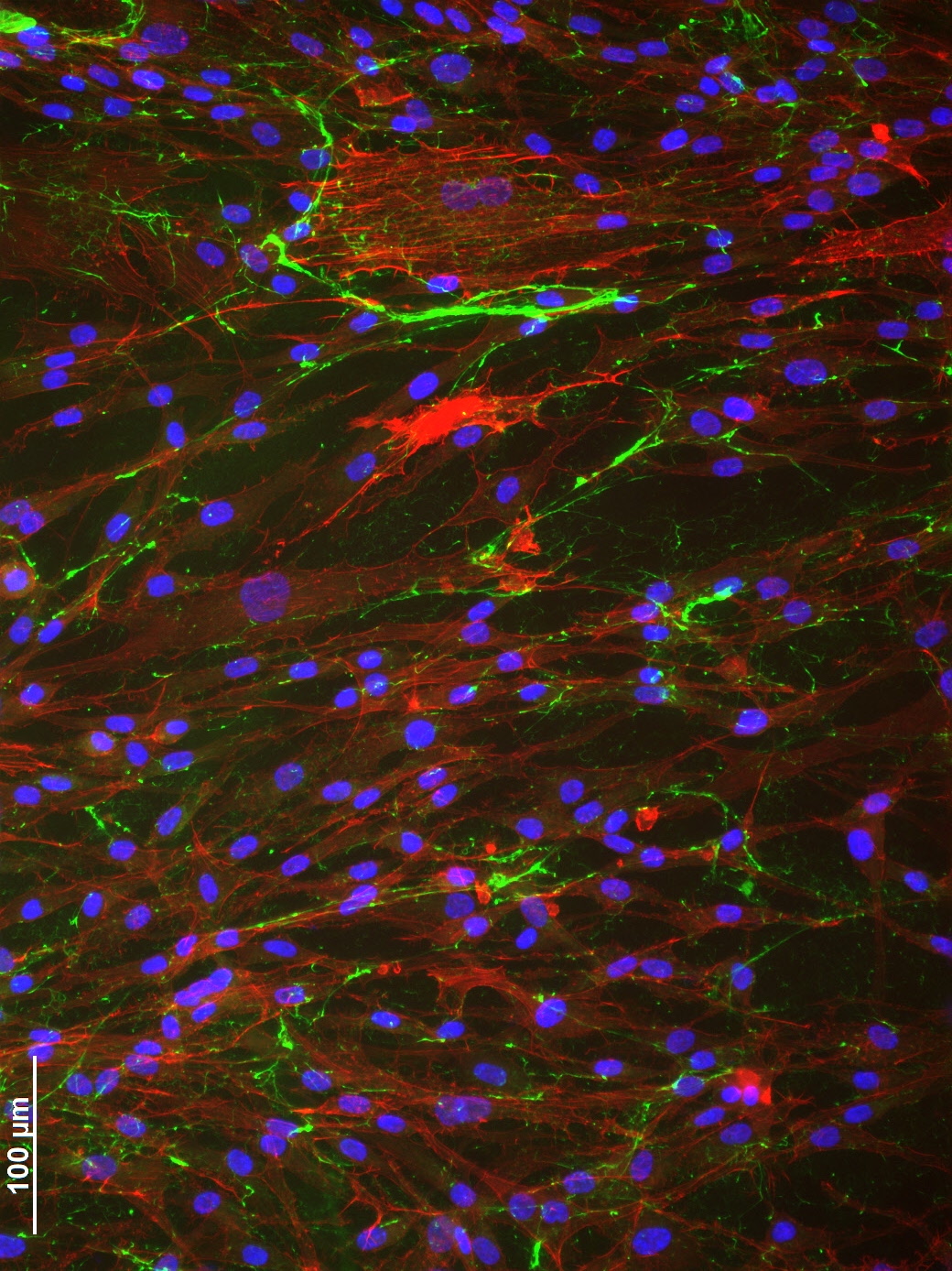

Supplement: S1 File — (ZIP) [file pone.0304645.s001.zip › ASCs_TNC_Fig2/14d_mFGF.JPG]

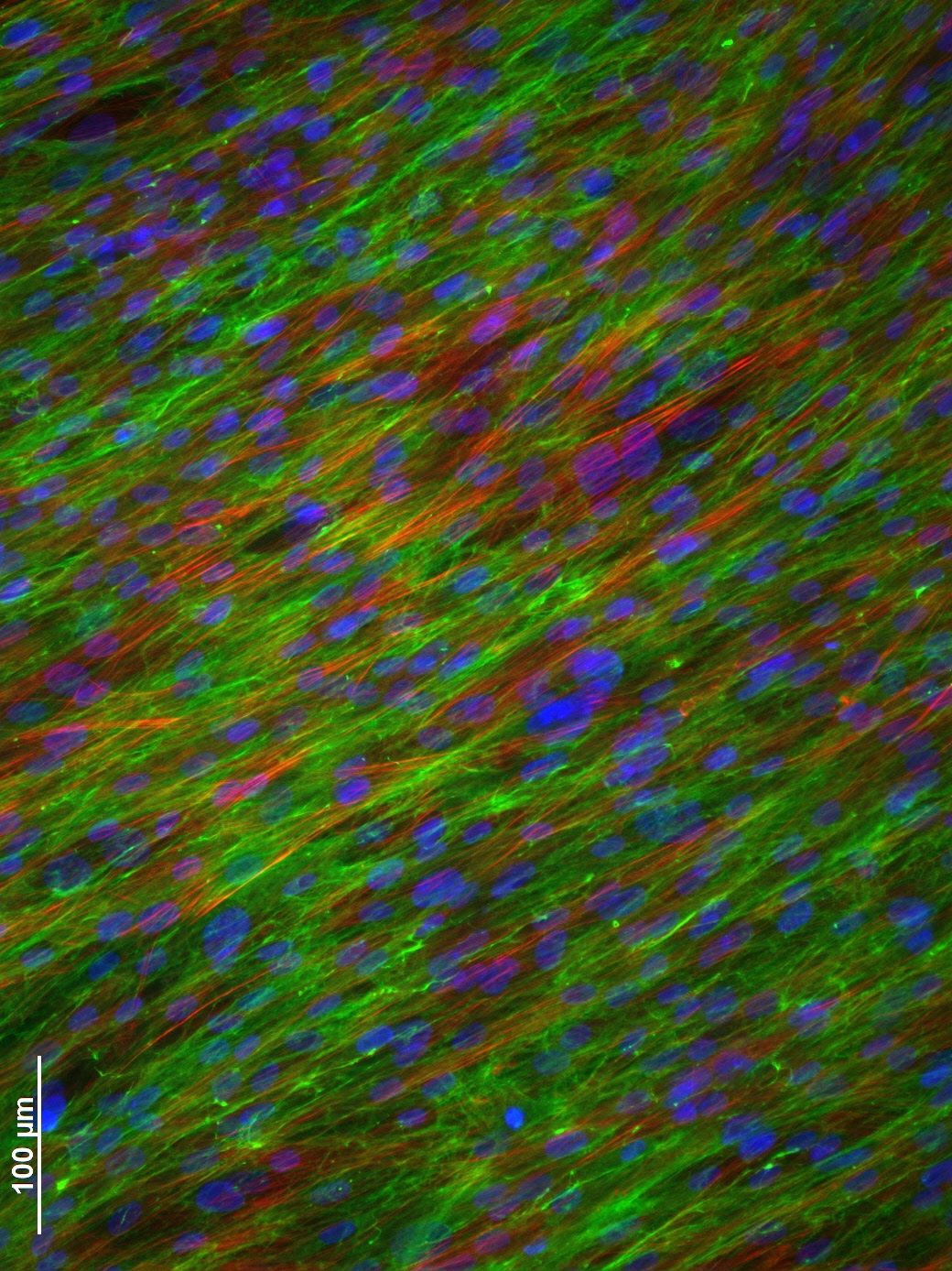

Supplement: S1 File — (ZIP) [file pone.0304645.s001.zip › ASCs_TNC_Fig2/14d_mPDGF-BB.JPG]

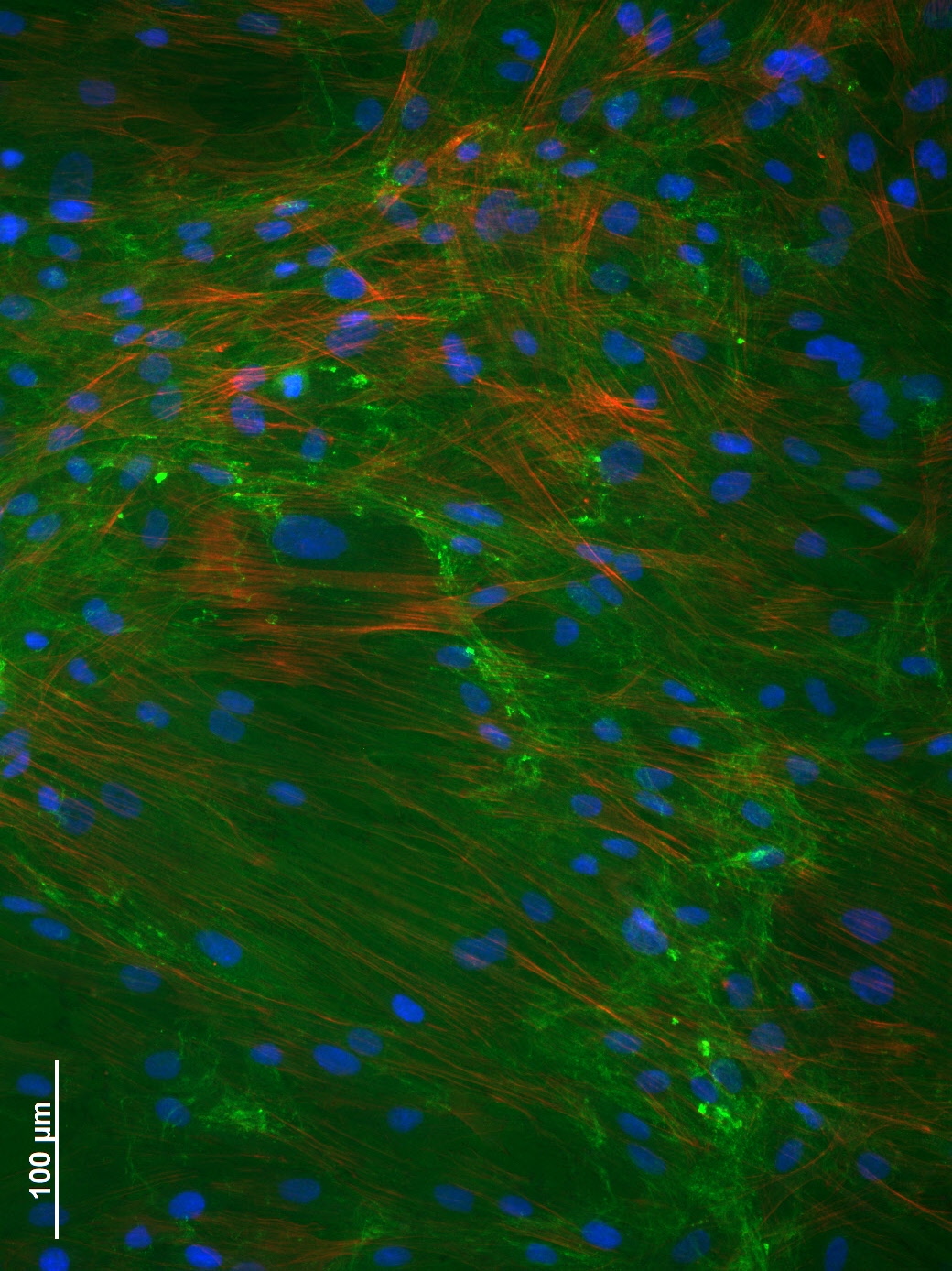

Supplement: S1 File — (ZIP) [file pone.0304645.s001.zip › ASCs_TNC_Fig2/14d_mTGF-B.JPG]

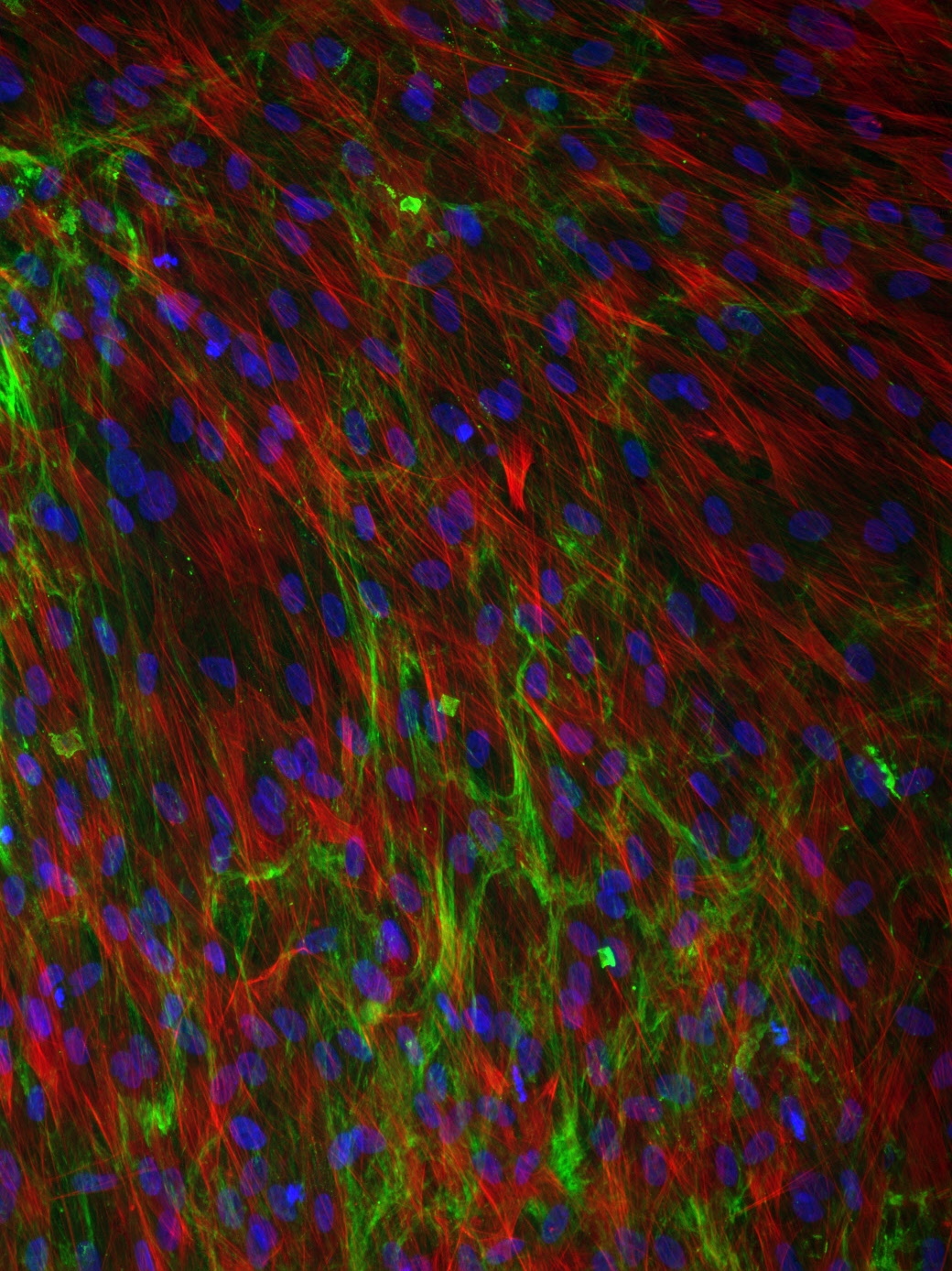

Supplement: S1 File — (ZIP) [file pone.0304645.s001.zip › ASCs_TNC_Fig2/21d mA.JPG]

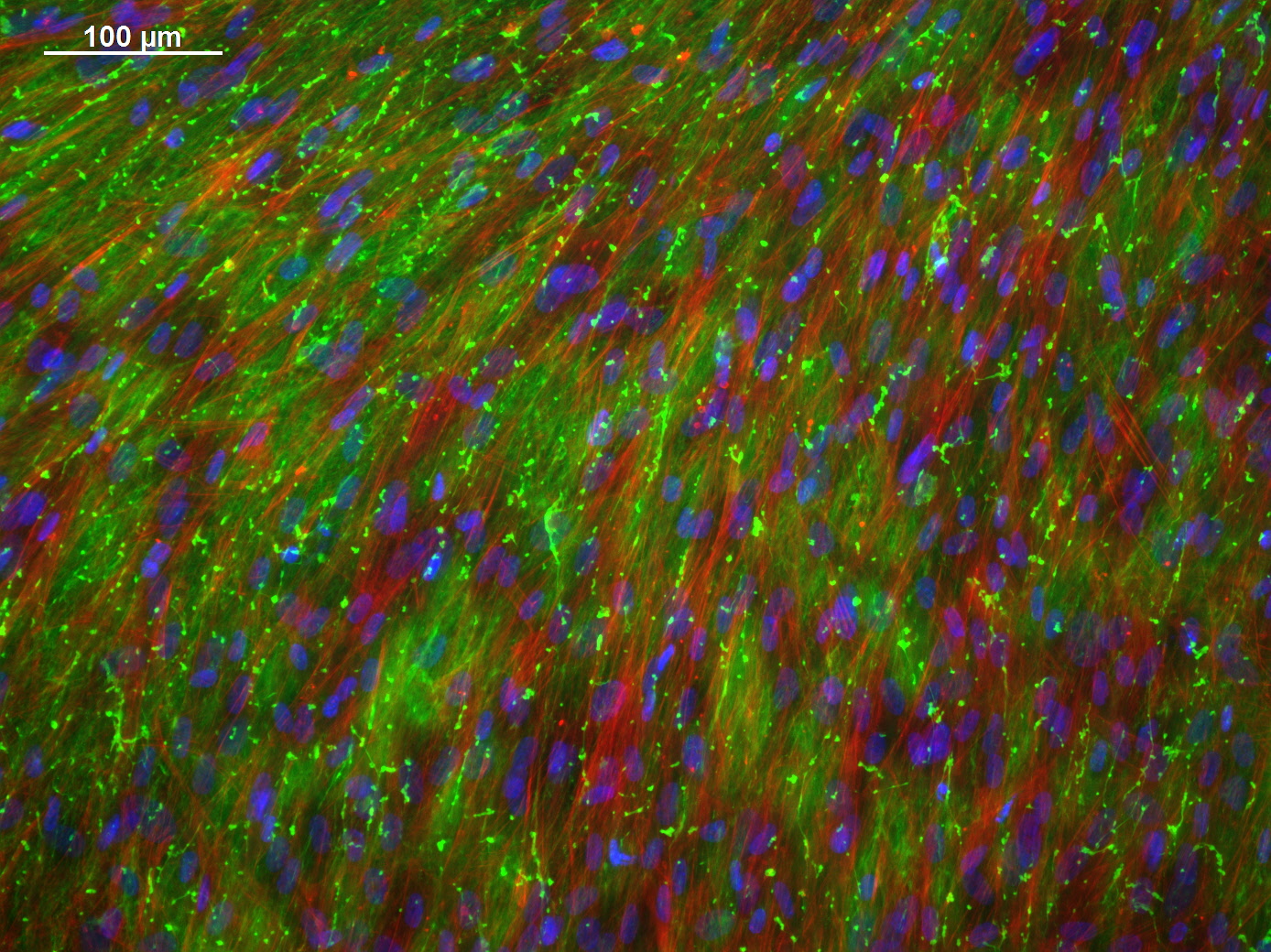

Supplement: S1 File — (ZIP) [file pone.0304645.s001.zip › ASCs_TNC_Fig2/21d_mC.JPG]

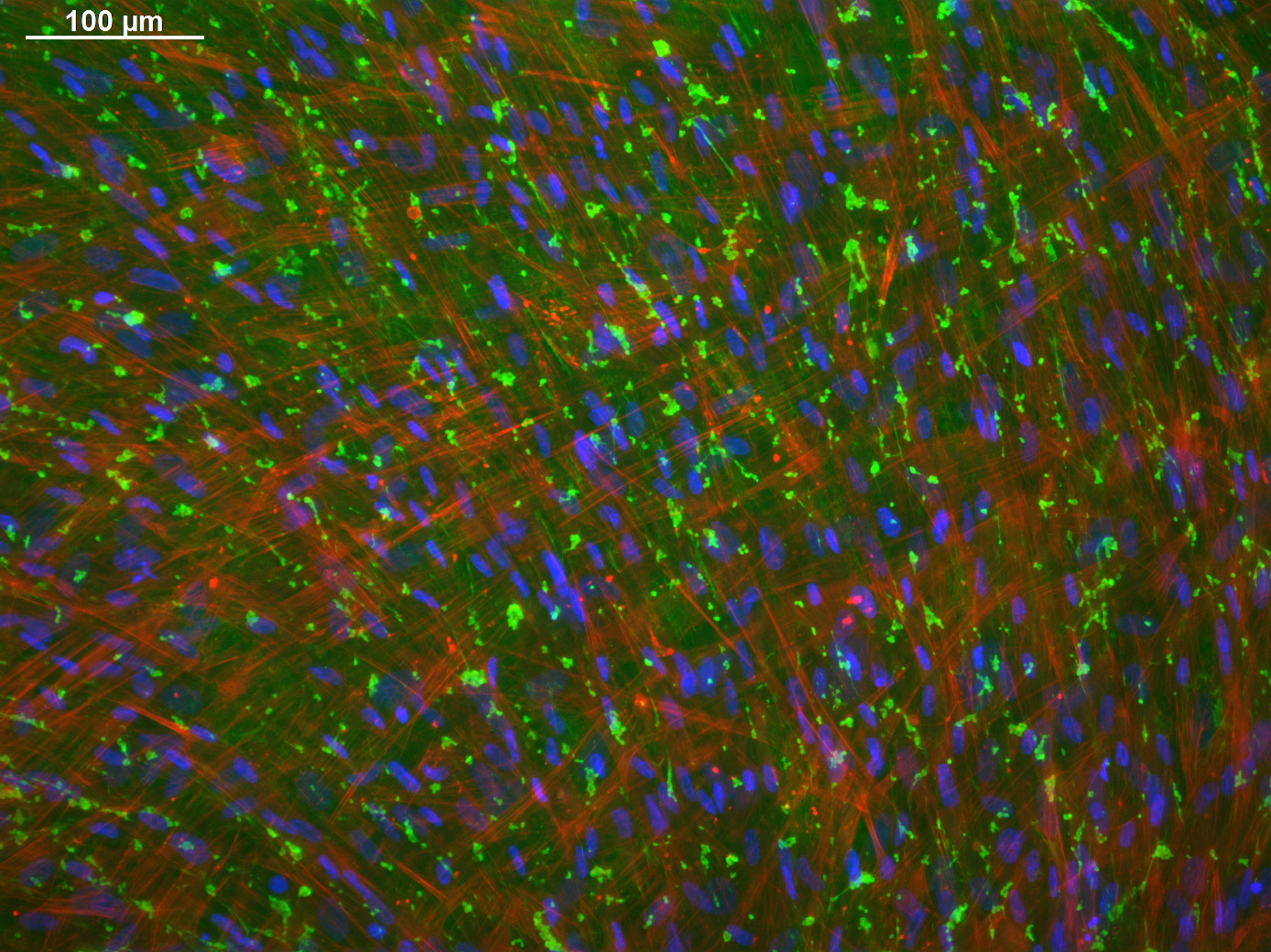

Supplement: S1 File — (ZIP) [file pone.0304645.s001.zip › ASCs_TNC_Fig2/21d_mEGF.JPG]

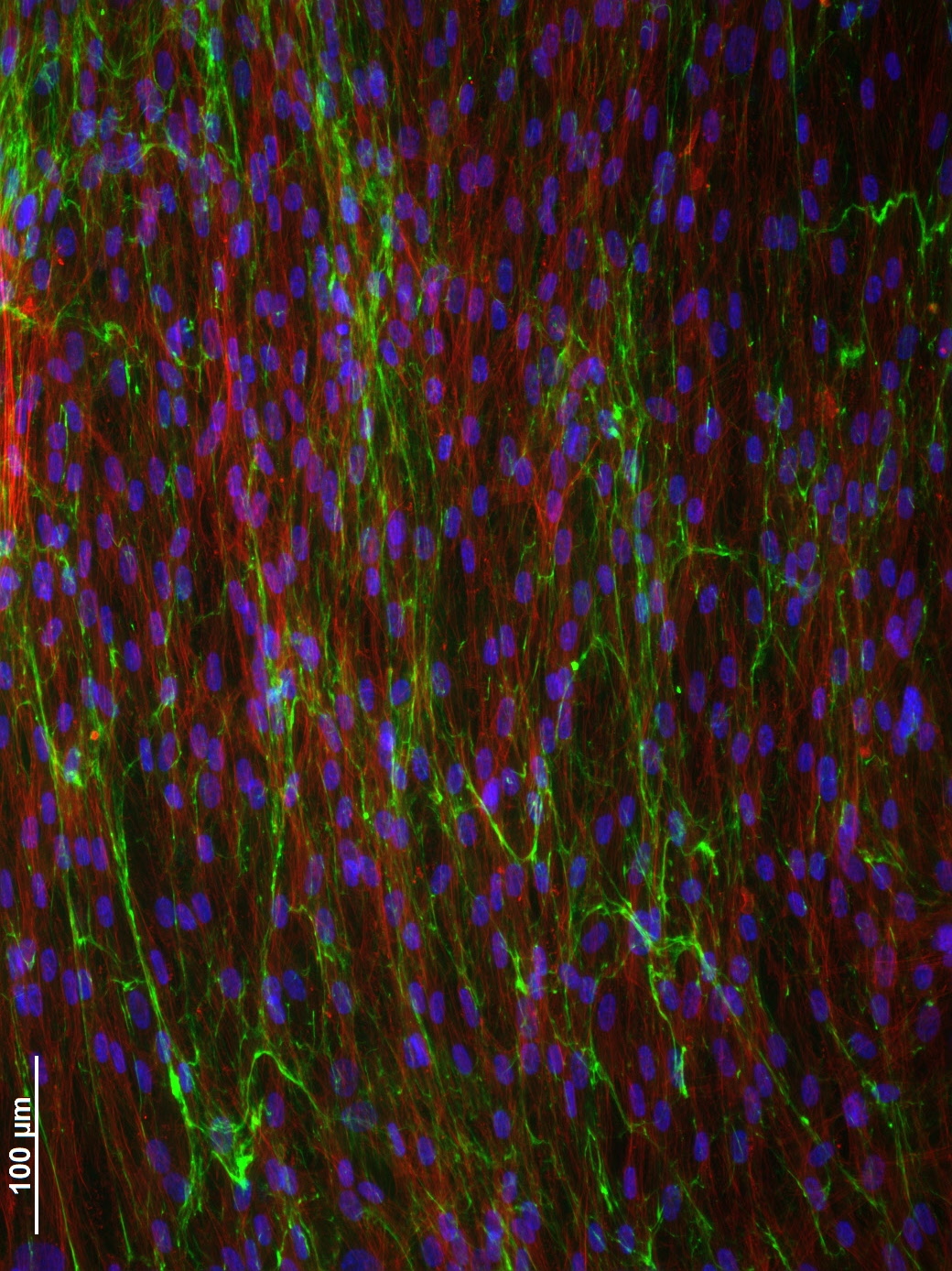

Supplement: S1 File — (ZIP) [file pone.0304645.s001.zip › ASCs_TNC_Fig2/21d_mFGF.JPG]

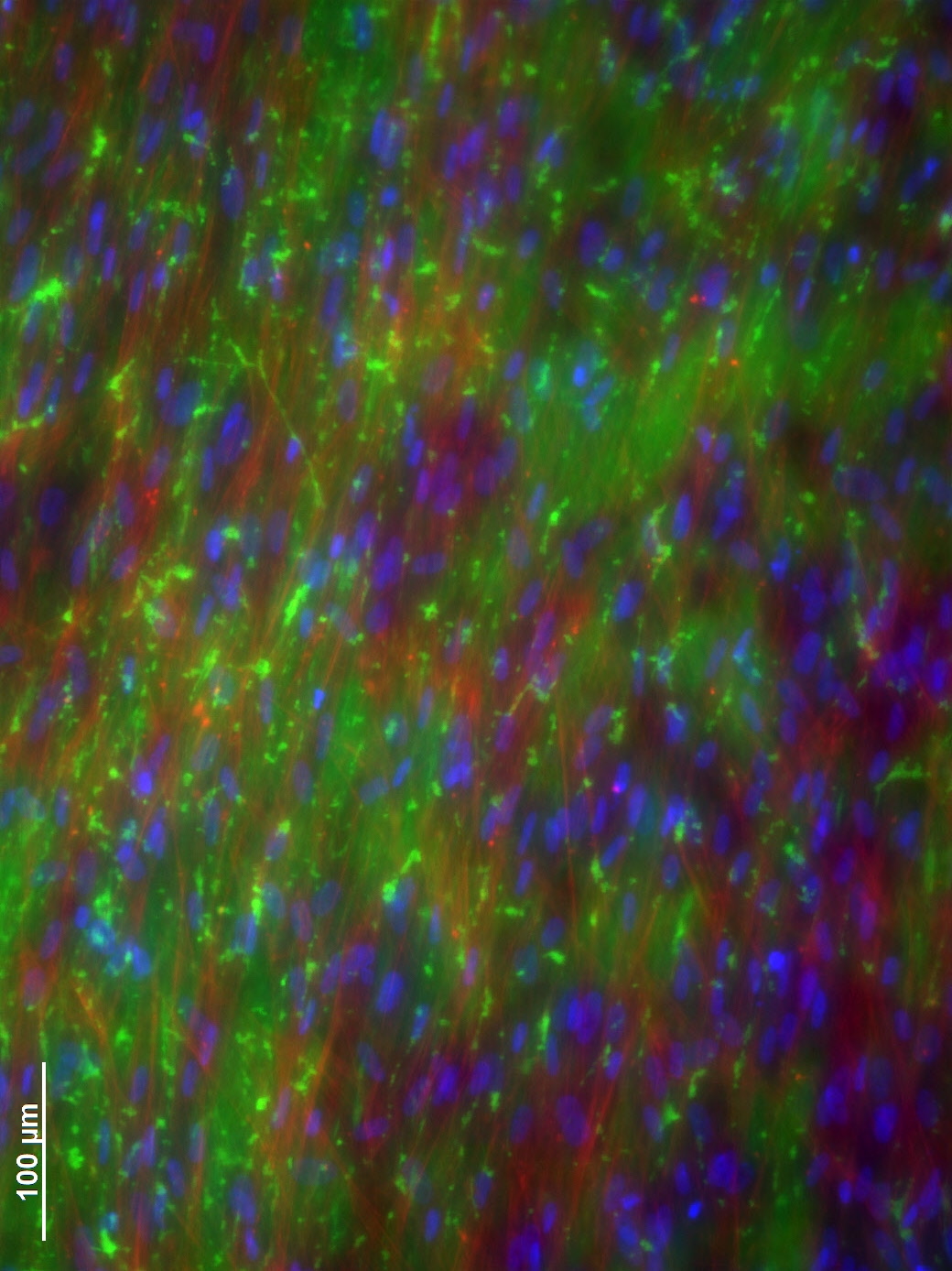

Supplement: S1 File — (ZIP) [file pone.0304645.s001.zip › ASCs_TNC_Fig2/21d_mPDGF-BB.JPG]

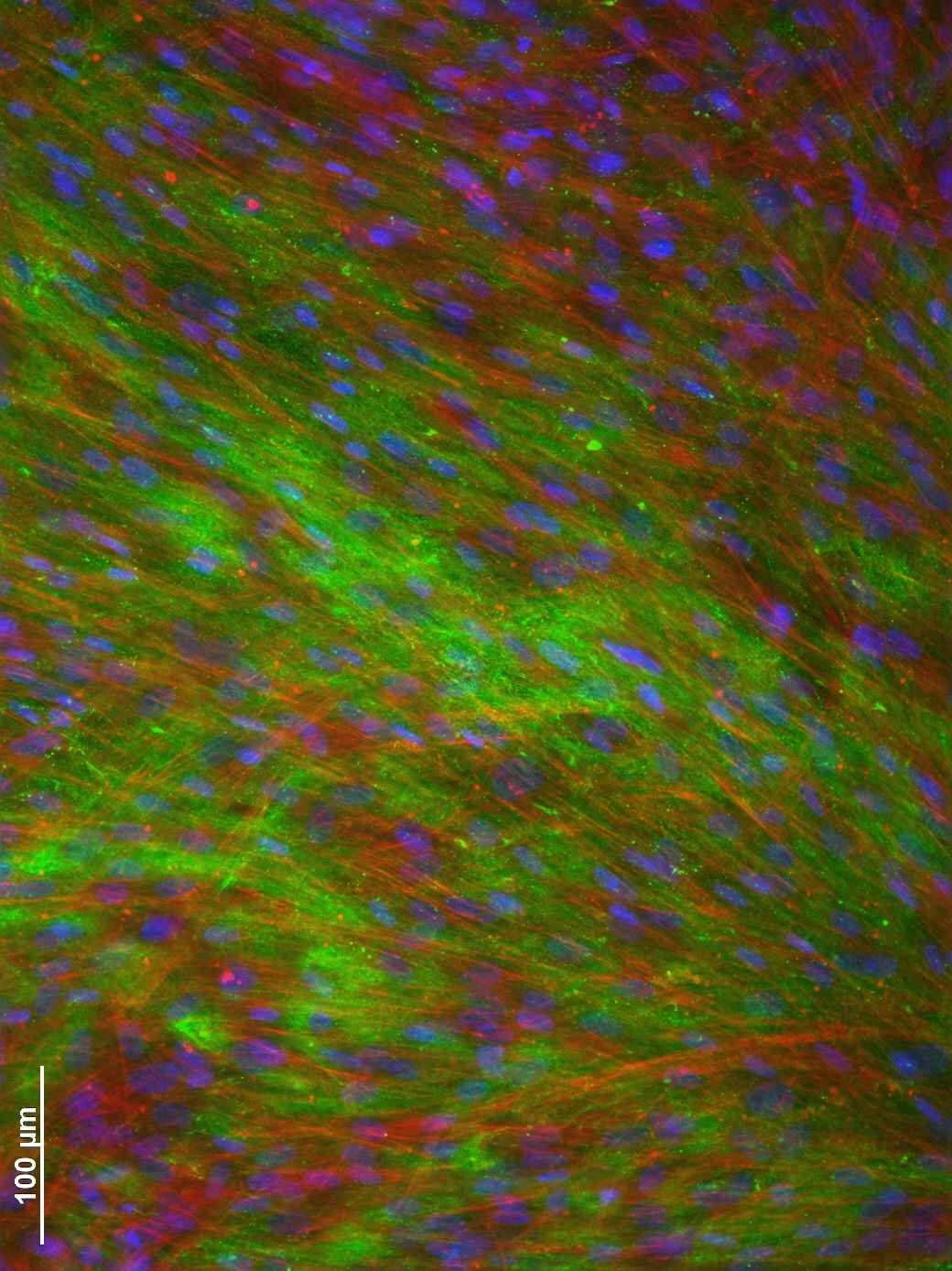

Supplement: S1 File — (ZIP) [file pone.0304645.s001.zip › ASCs_TNC_Fig2/21d_mTGF-B.JPG]

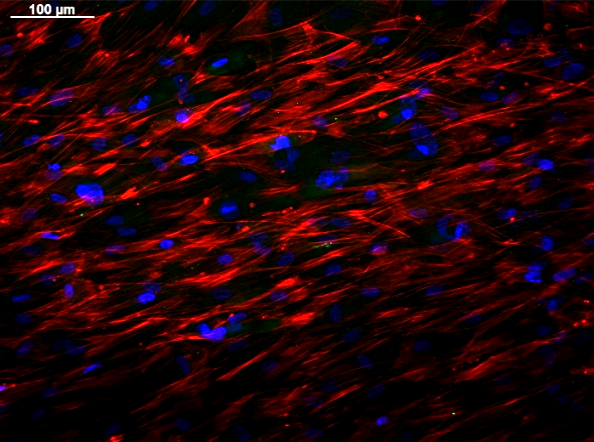

Supplement: S1 File — (ZIP) [file pone.0304645.s001.zip › ASCs_TNC_Fig2/28d_mA.png]

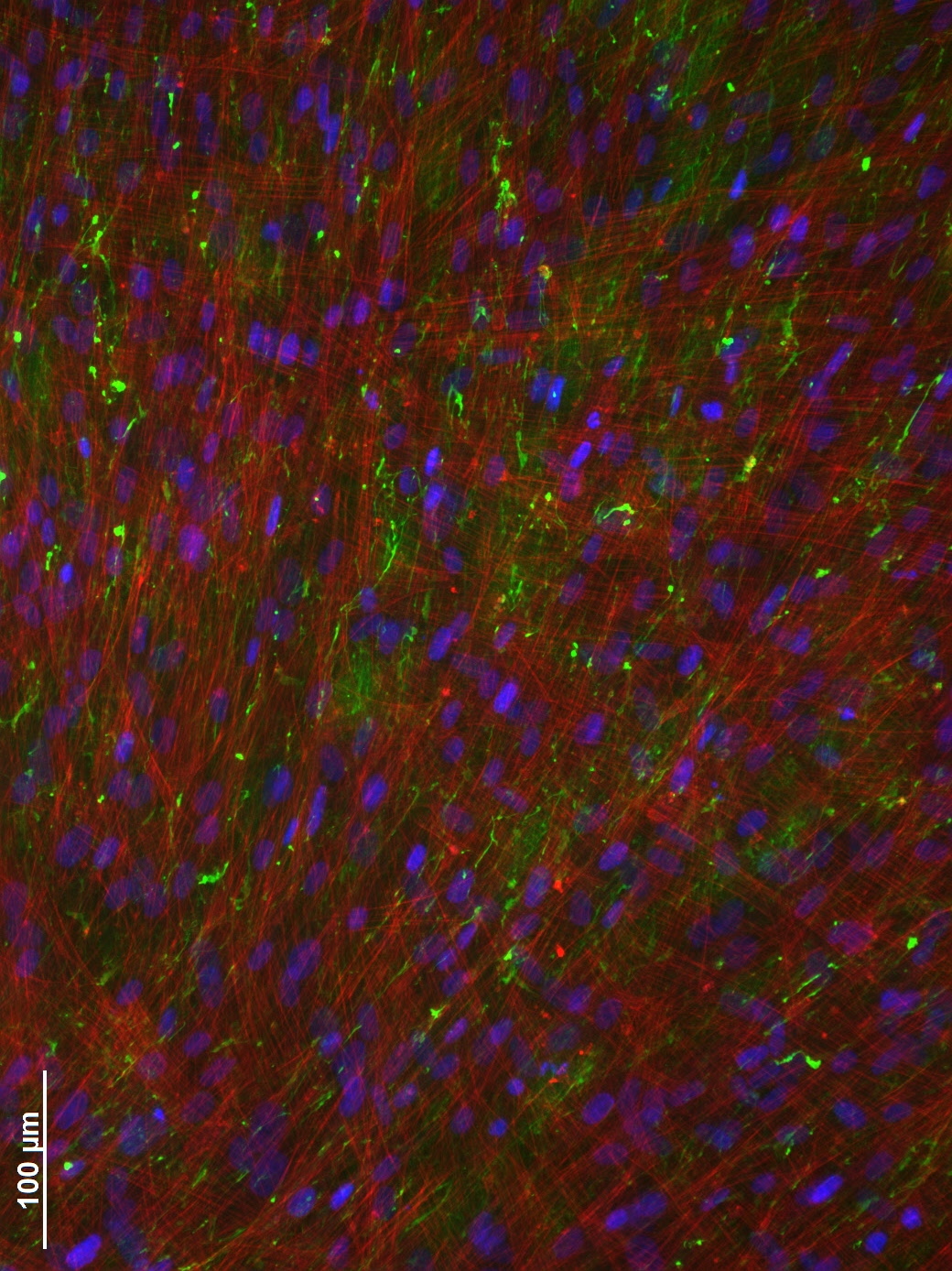

Supplement: S1 File — (ZIP) [file pone.0304645.s001.zip › ASCs_TNC_Fig2/28d_mC.JPG]

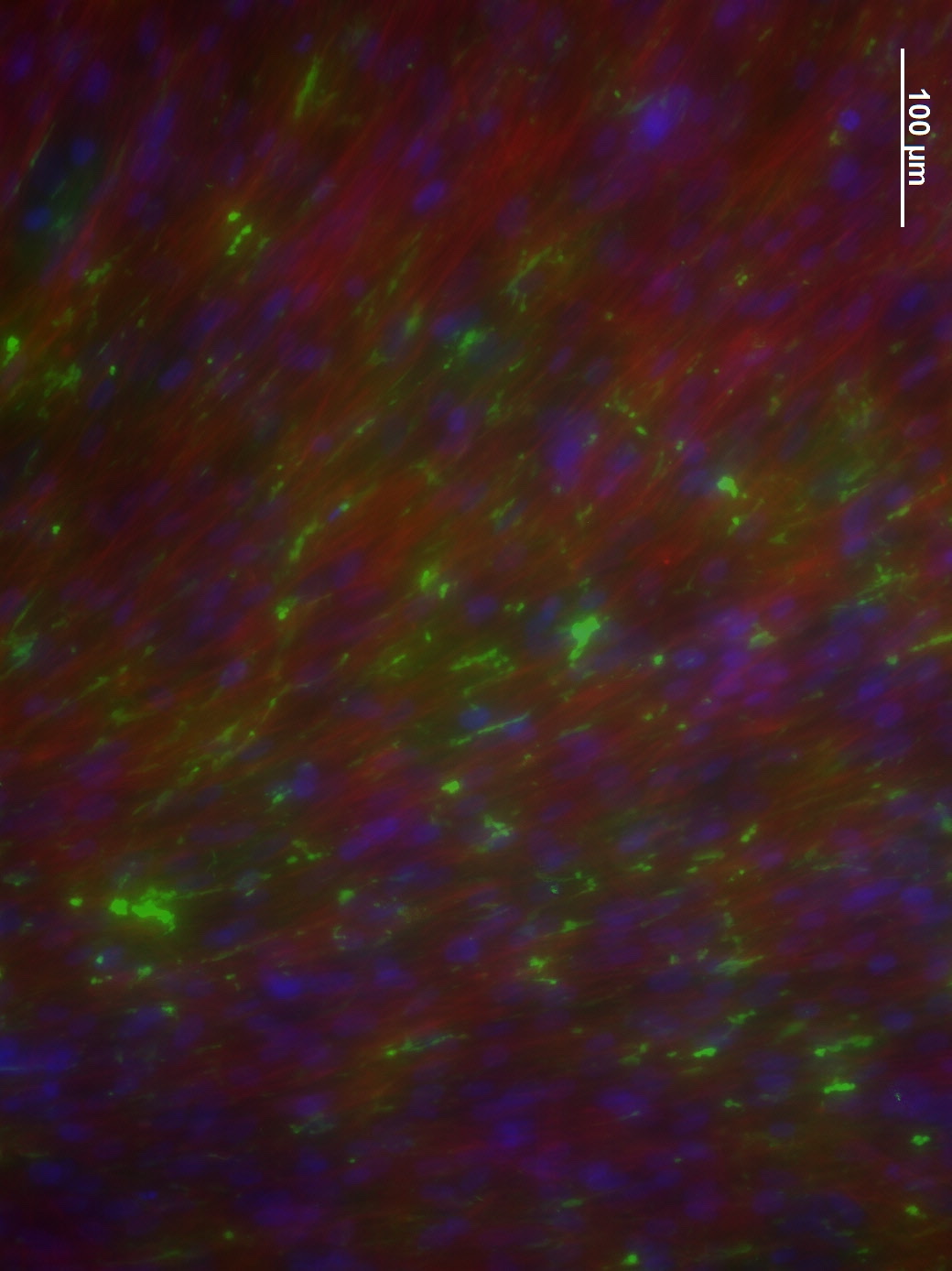

Supplement: S1 File — (ZIP) [file pone.0304645.s001.zip › ASCs_TNC_Fig2/28d_mEGF.JPG]

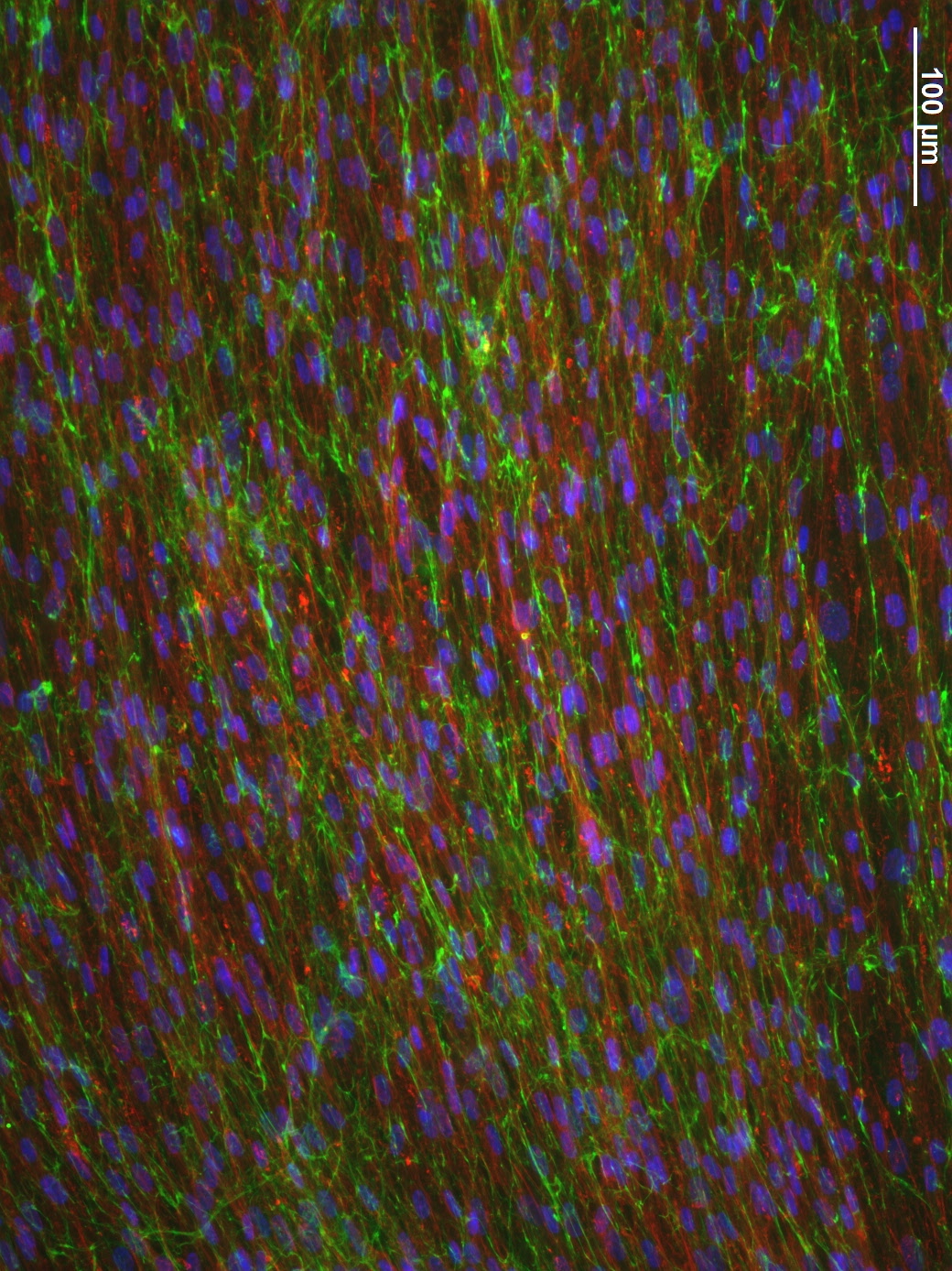

Supplement: S1 File — (ZIP) [file pone.0304645.s001.zip › ASCs_TNC_Fig2/28d_mFGF.JPG]

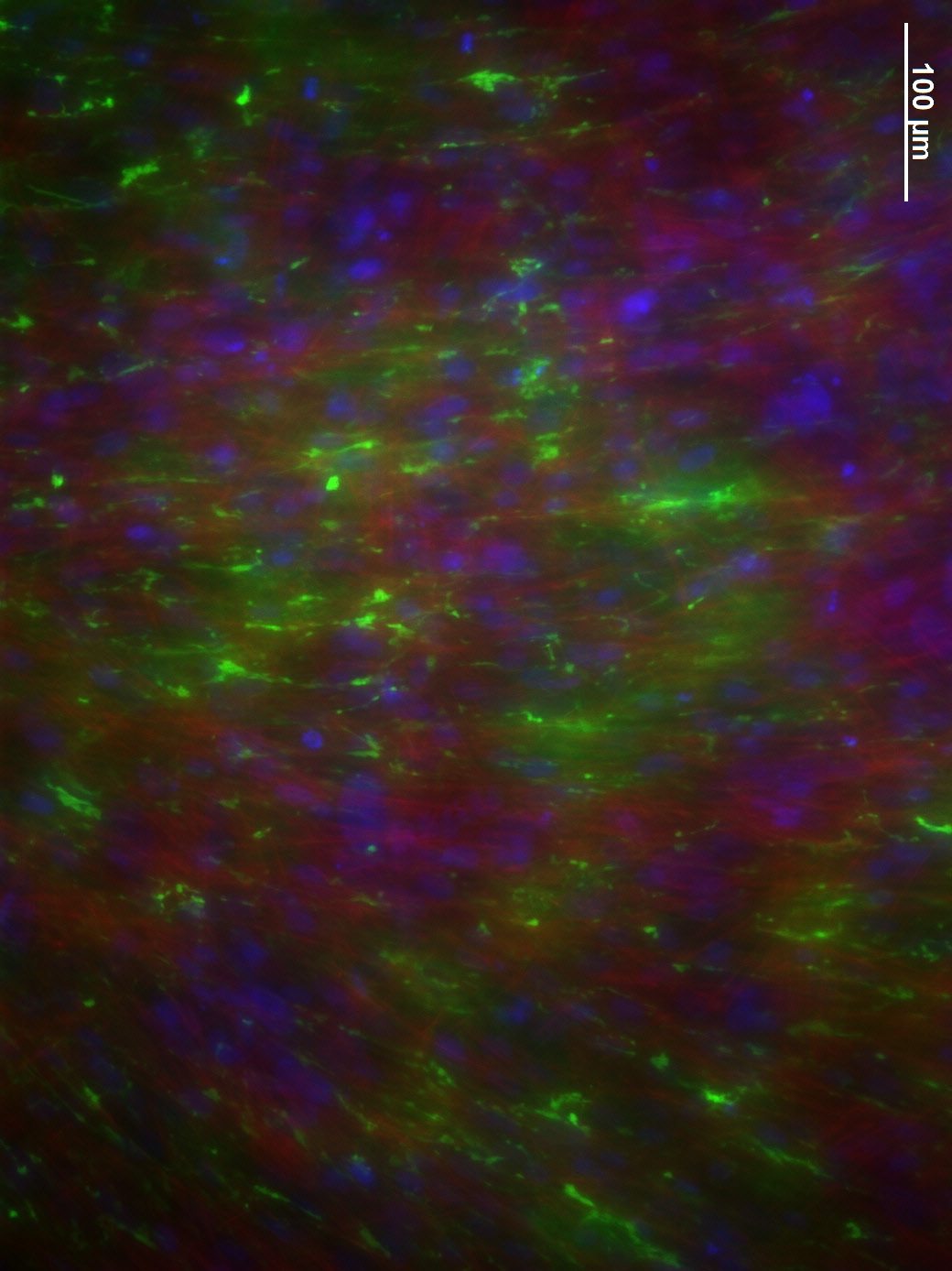

Supplement: S1 File — (ZIP) [file pone.0304645.s001.zip › ASCs_TNC_Fig2/28d_mPDGF-BB.JPG]

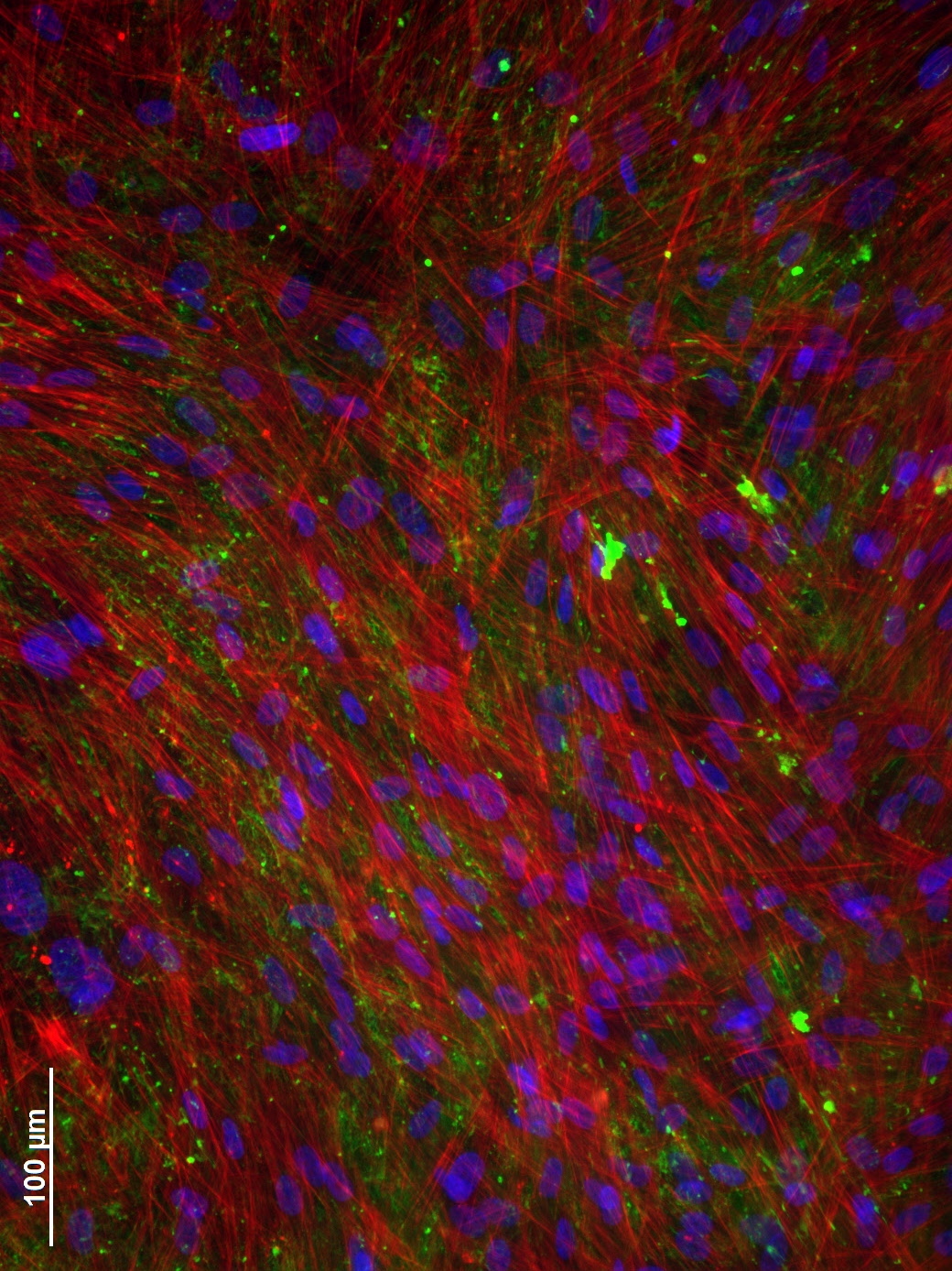

Supplement: S1 File — (ZIP) [file pone.0304645.s001.zip › ASCs_TNC_Fig2/28d_mTGF-B.JPG]

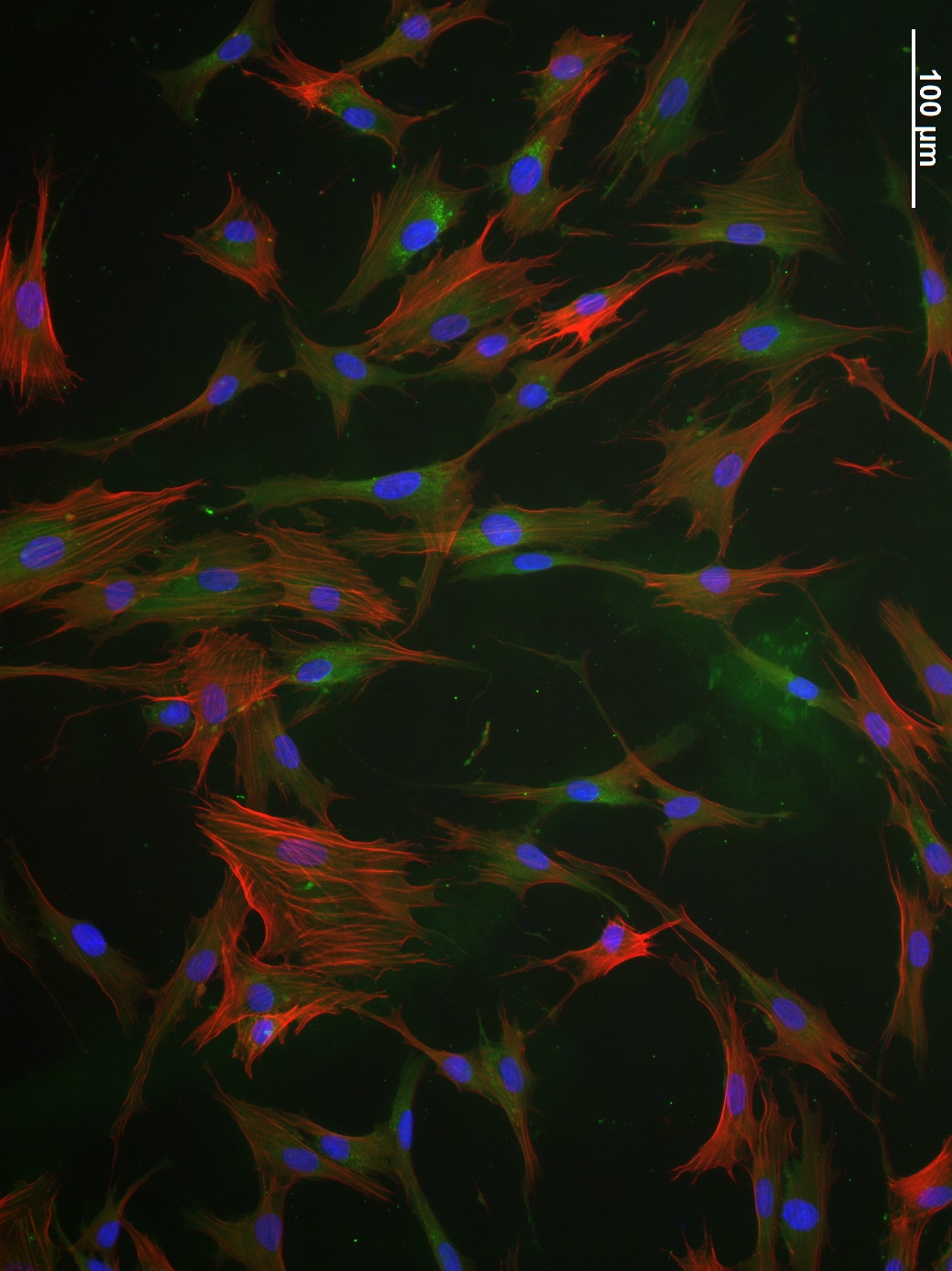

Supplement: S1 File — (ZIP) [file pone.0304645.s001.zip › ASCs_TNC_Fig2/7d_mA.JPG]

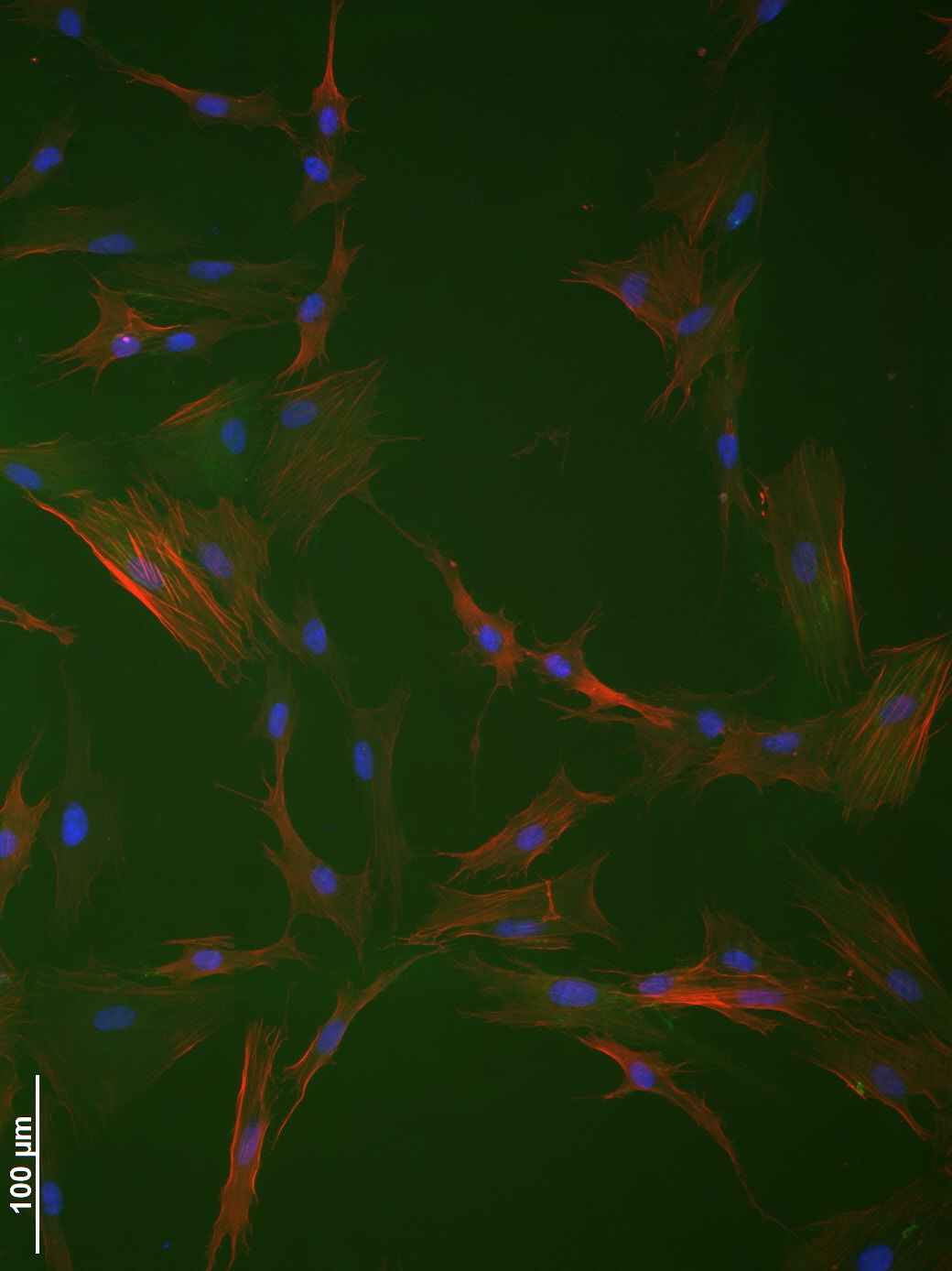

Supplement: S1 File — (ZIP) [file pone.0304645.s001.zip › ASCs_TNC_Fig2/7d_mC.JPG]

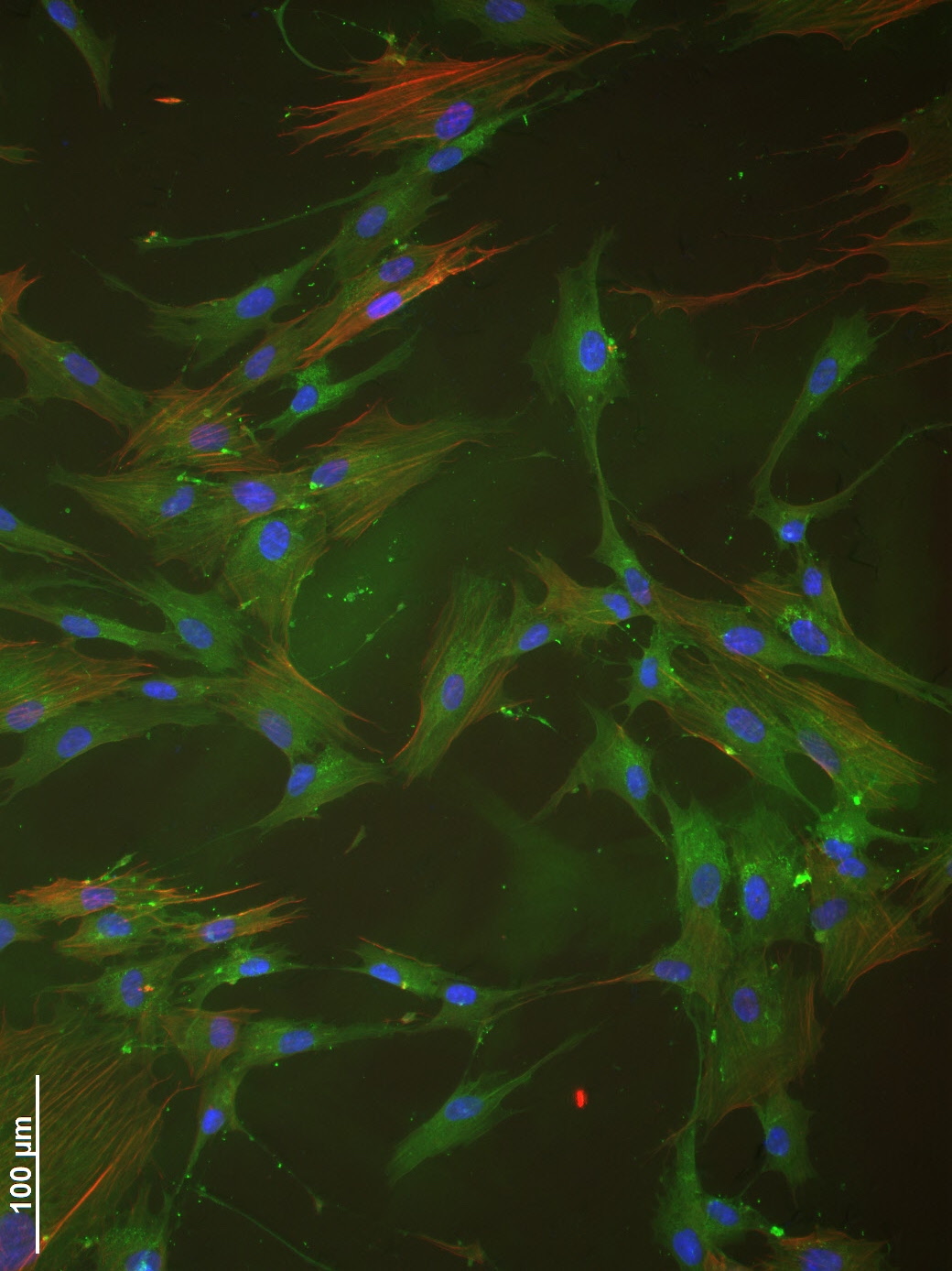

Supplement: S1 File — (ZIP) [file pone.0304645.s001.zip › ASCs_TNC_Fig2/7d_mEGF.JPG]

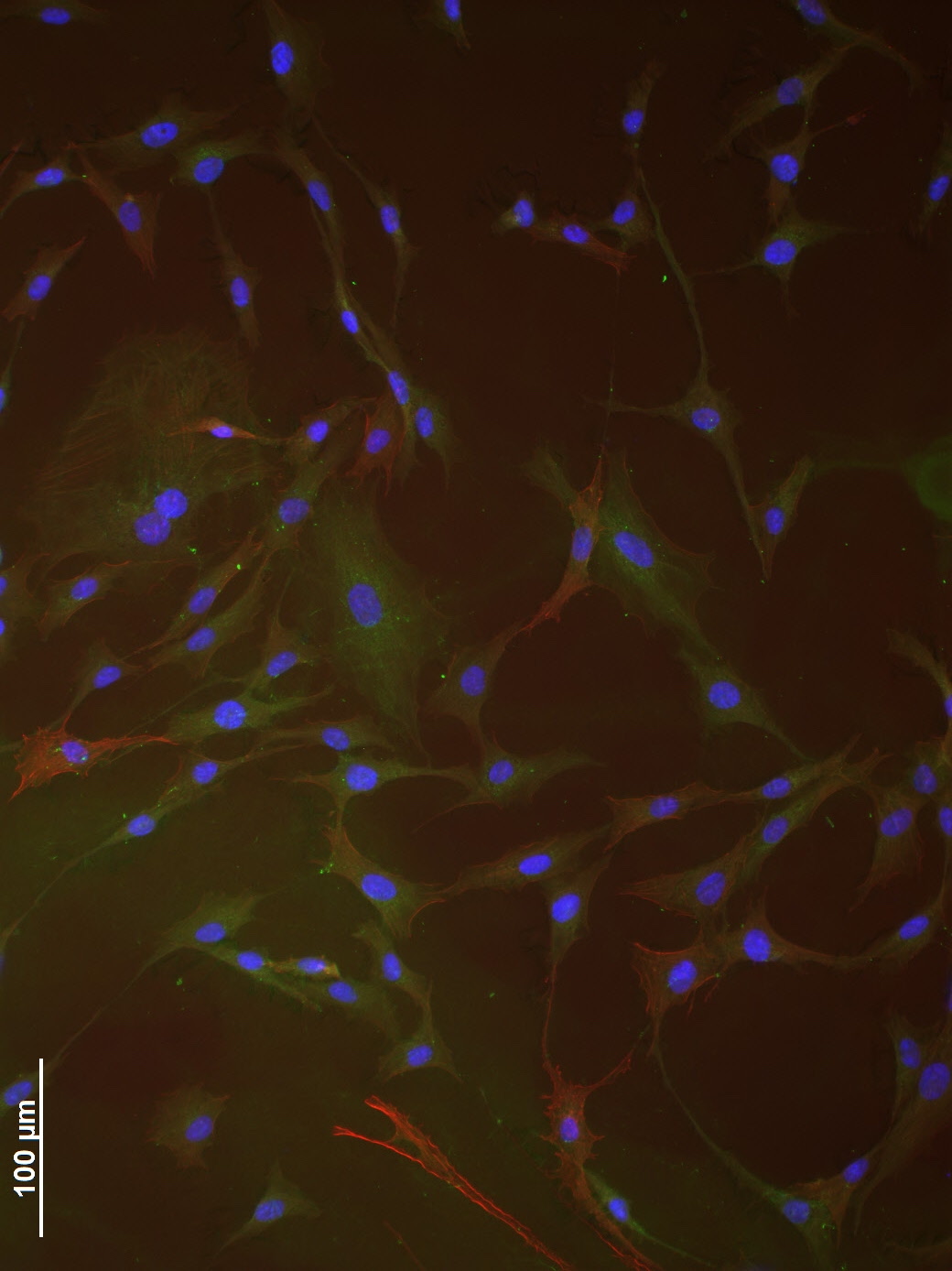

Supplement: S1 File — (ZIP) [file pone.0304645.s001.zip › ASCs_TNC_Fig2/7d_mFGF.JPG]

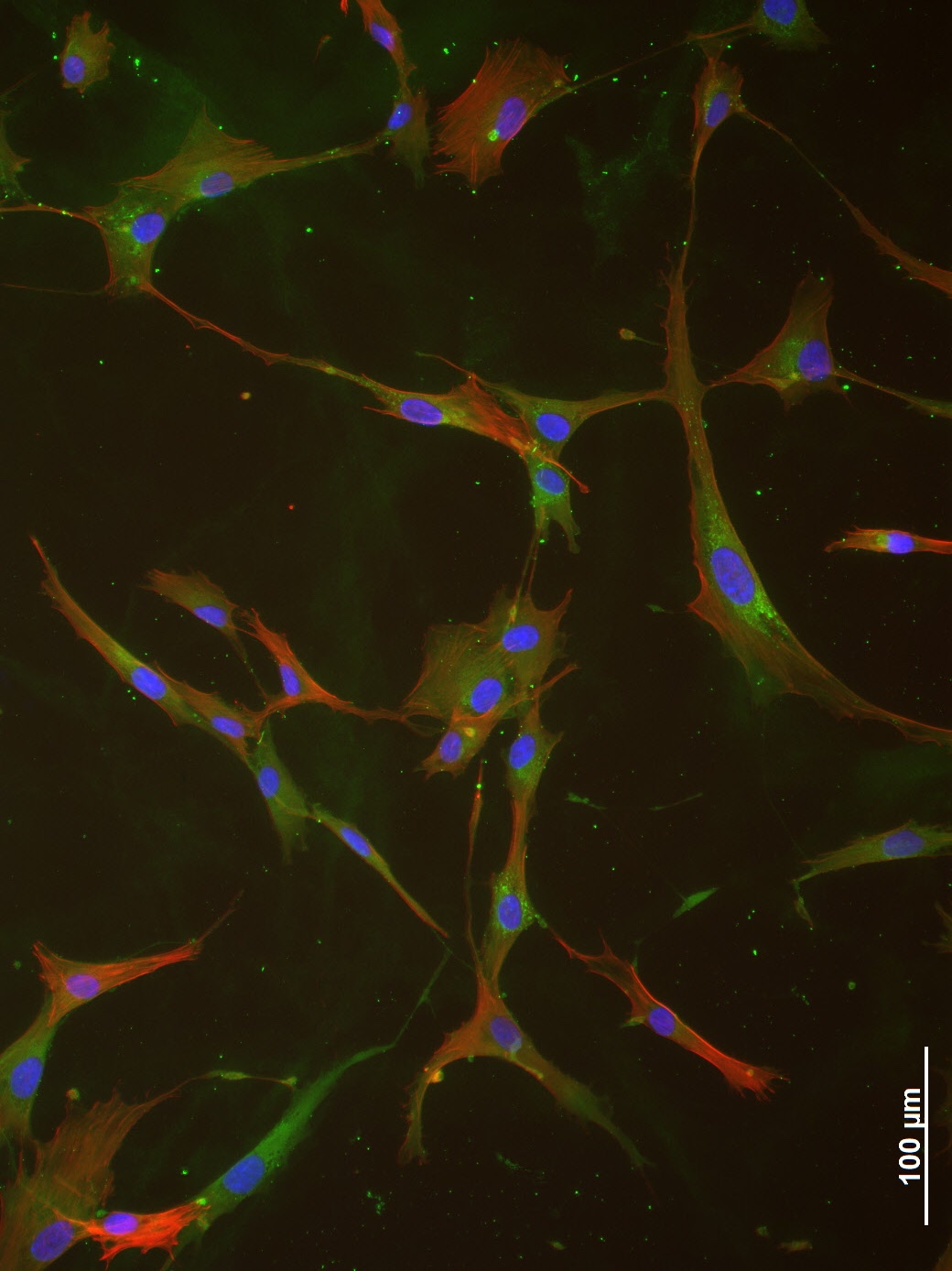

Supplement: S1 File — (ZIP) [file pone.0304645.s001.zip › ASCs_TNC_Fig2/7d_mPDGF-BB.JPG]

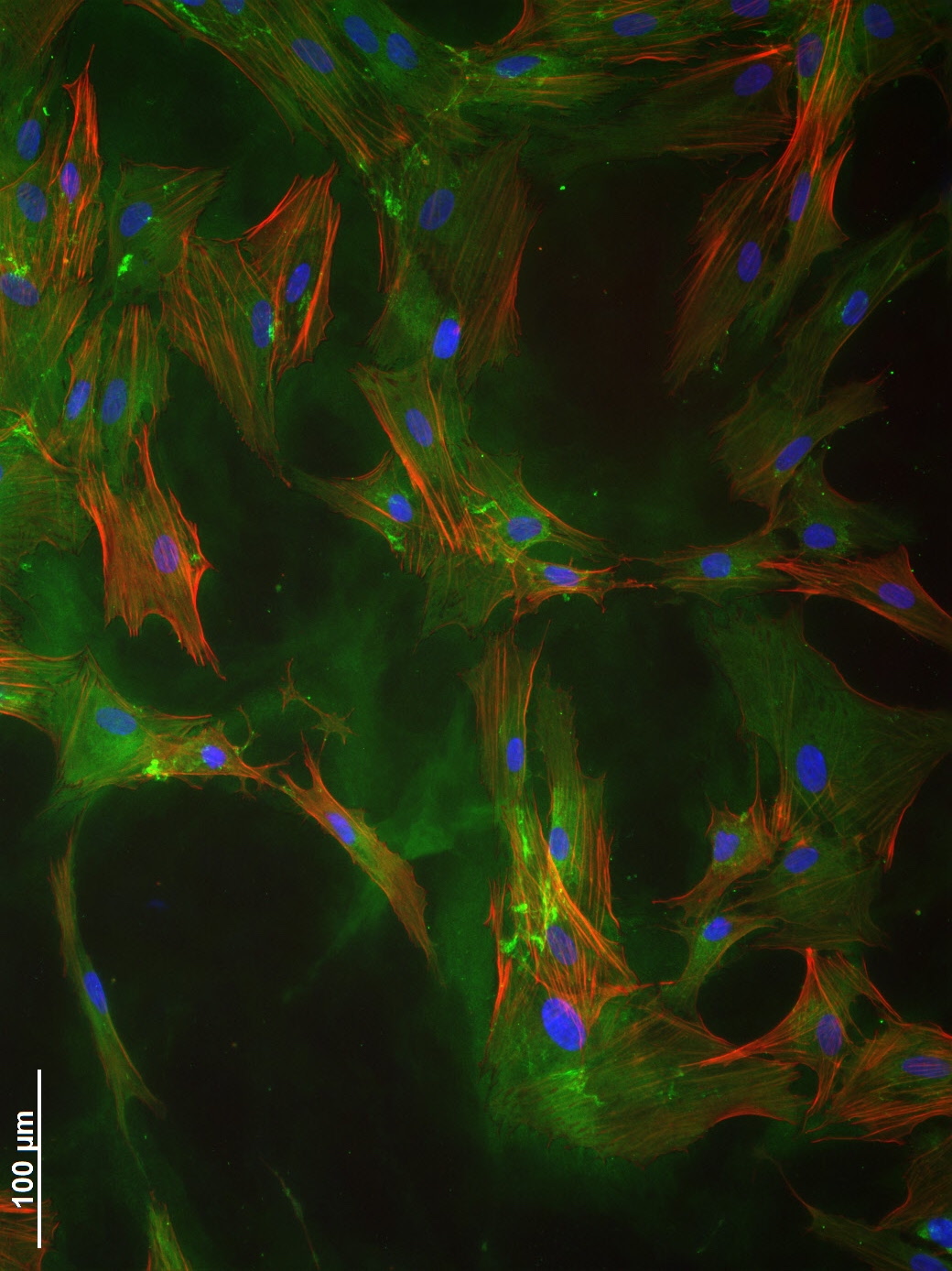

Supplement: S1 File — (ZIP) [file pone.0304645.s001.zip › ASCs_TNC_Fig2/7d_mTGF-B.JPG]
